# Supplementary material for: Single-cell sequencing reveals increased LAMB3-positive basal keratinocytes and ZNF90-positive fibroblasts in autologous cultured epithelium
Source: Commun Biol. 2024 Jan 10;7:79. doi: 10.1038/s42003-023-05747-5 (PMC10781733; doi:10.1038/s42003-023-05747-5)
Supplement: Supplementary file 2 — Supplementary Information [file 42003_2023_5747_MOESM2_ESM.pdf]

Supplementary Information

Single-cell sequencing reveals increased LAMB3-positive basal keratinocytes and ZNF90-positive fibroblasts in autologous cultured epithelium

Authors and affiliations:

Weiling Lian[1, †], Xuanhao Zeng[1, †], Jian Li[1, †], Qing Zang[1, †], Yating Liu[1], Haozhen Lv[1], Shujun Chen[1], Shiyi Huang[1], Jiayi Shen[1], Luyan Tang[1], Yu Xu[1], Fuyue Wu[2], Qi Zhang[1, ※], Jinhua Xu[1, ※]

1. Department of Dermatology, Huashan Hospital, Fudan University, Shanghai Institute of Dermatology, Shanghai, China
2. ReMed Regenerative Medicine Clinical Application Institute, Shanghai, China

†Those authors contribute equally to this article.

※Corresponding author:

Qi Zhang, MD

E-mail: zhangqi@huashan.org.cn

Jinhua Xu, MD

E-mail: jinhuaxu@fudan.edu.cn

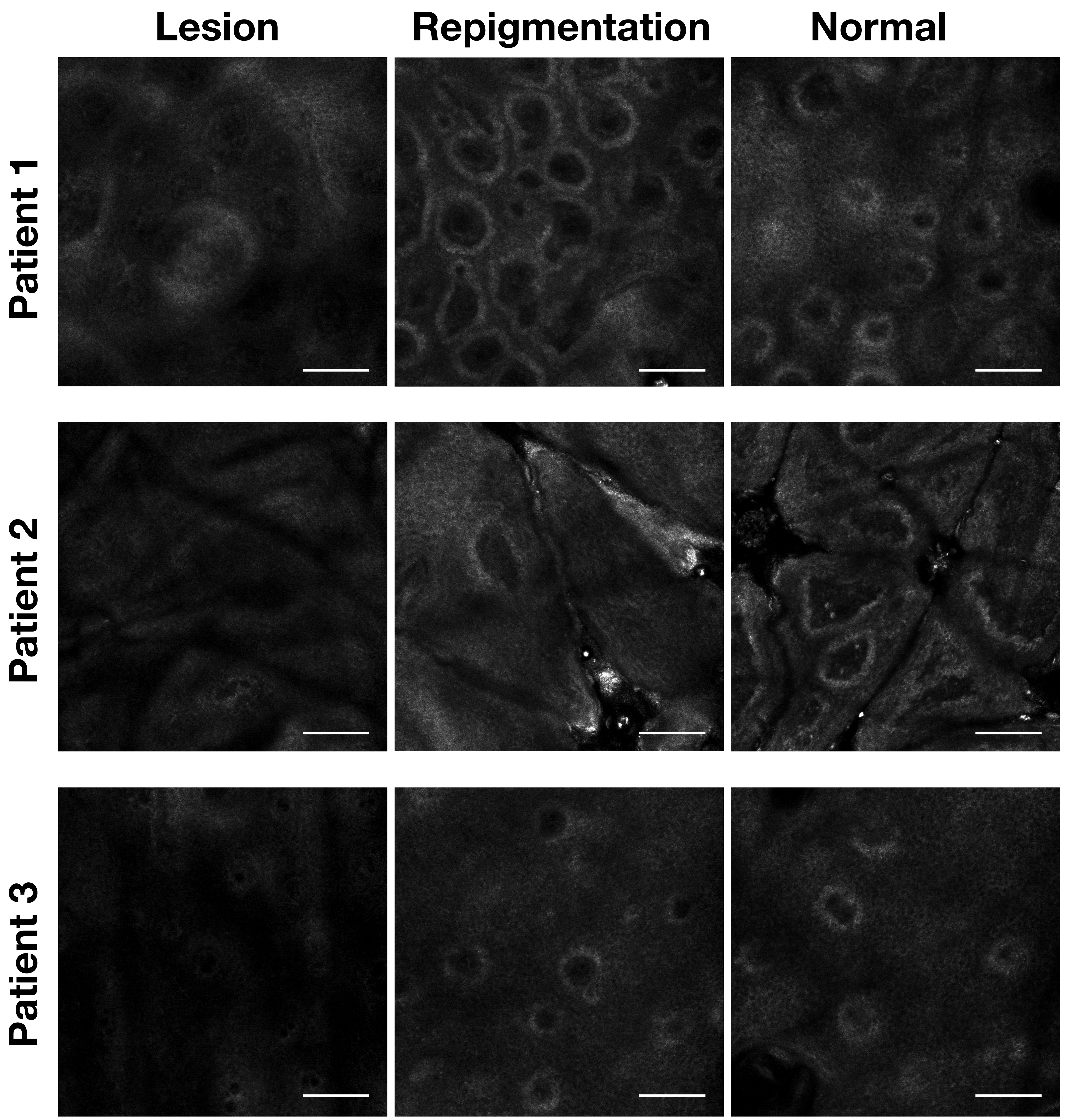

Supplementary Fig. S1 Reflectance confocal microscopy (RCM) imaging of lesional skin, post-operative regimented skin, and normal skin of 3 included patients. RCM images of lesional skin in vitiligo showed unclear border and loss of rings (Column 1), compared to those of normal skin (Column 3), that presented rings, half rings or scalloped border-like rings. RCM images of repigmented skin at 1-year follow-up showed the returning of borders and rings at the epidermal-dermal junction level (Column 2), compared to those of normal skin. Scale bar = 100 μm.

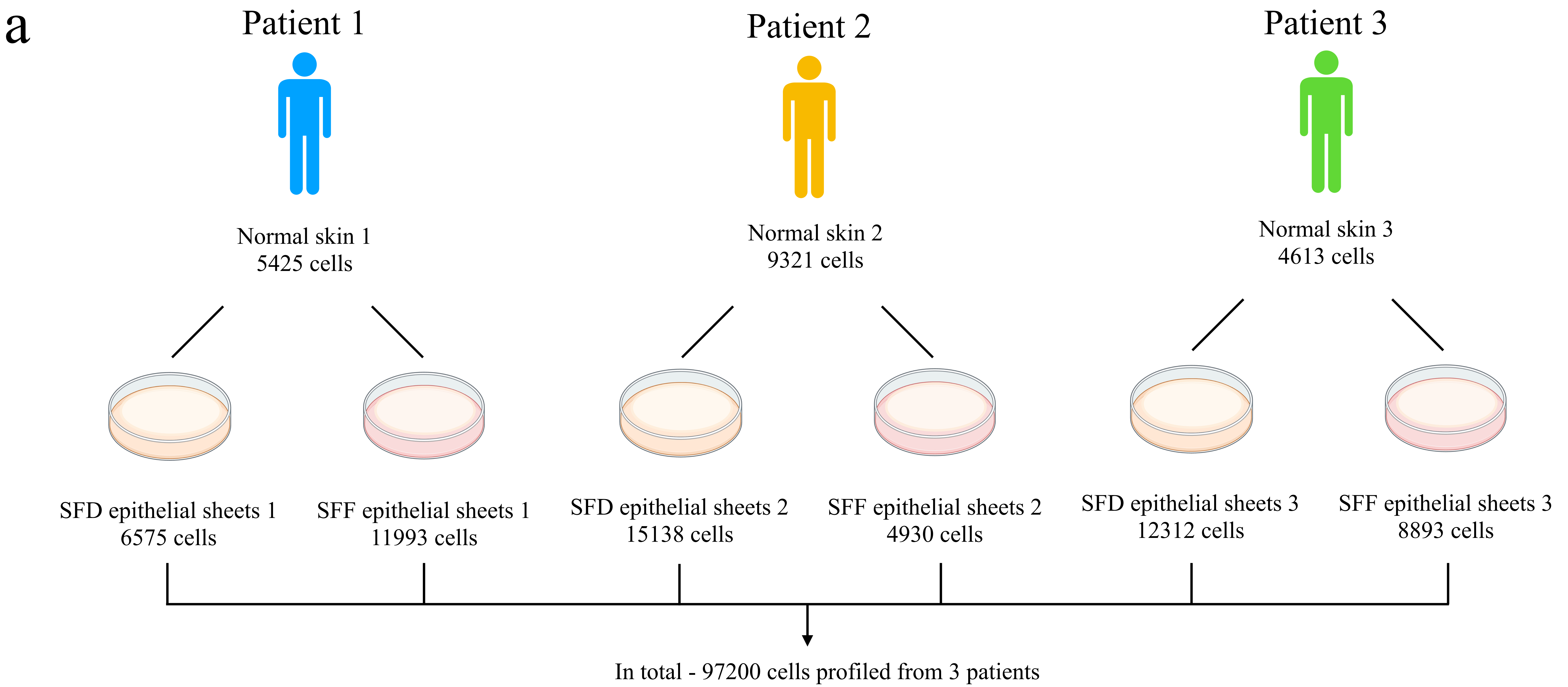
a
Patient 1
Patient 2
Patient 3
Normal skin 1
5425 cells
Normal skin 2
9321 cells
Normal skin 3
4613 cells
SFD epithelial sheets 1
6575 cells
SFF epithelial sheets 1
11993 cells
SFD epithelial sheets 2
15138 cells
SFF epithelial sheets 2
4930 cells
SFD epithelial sheets 3
12312 cells
SFF epithelial sheets 3
8893 cells
In total - 97200 cells profiled from 3 patients

b

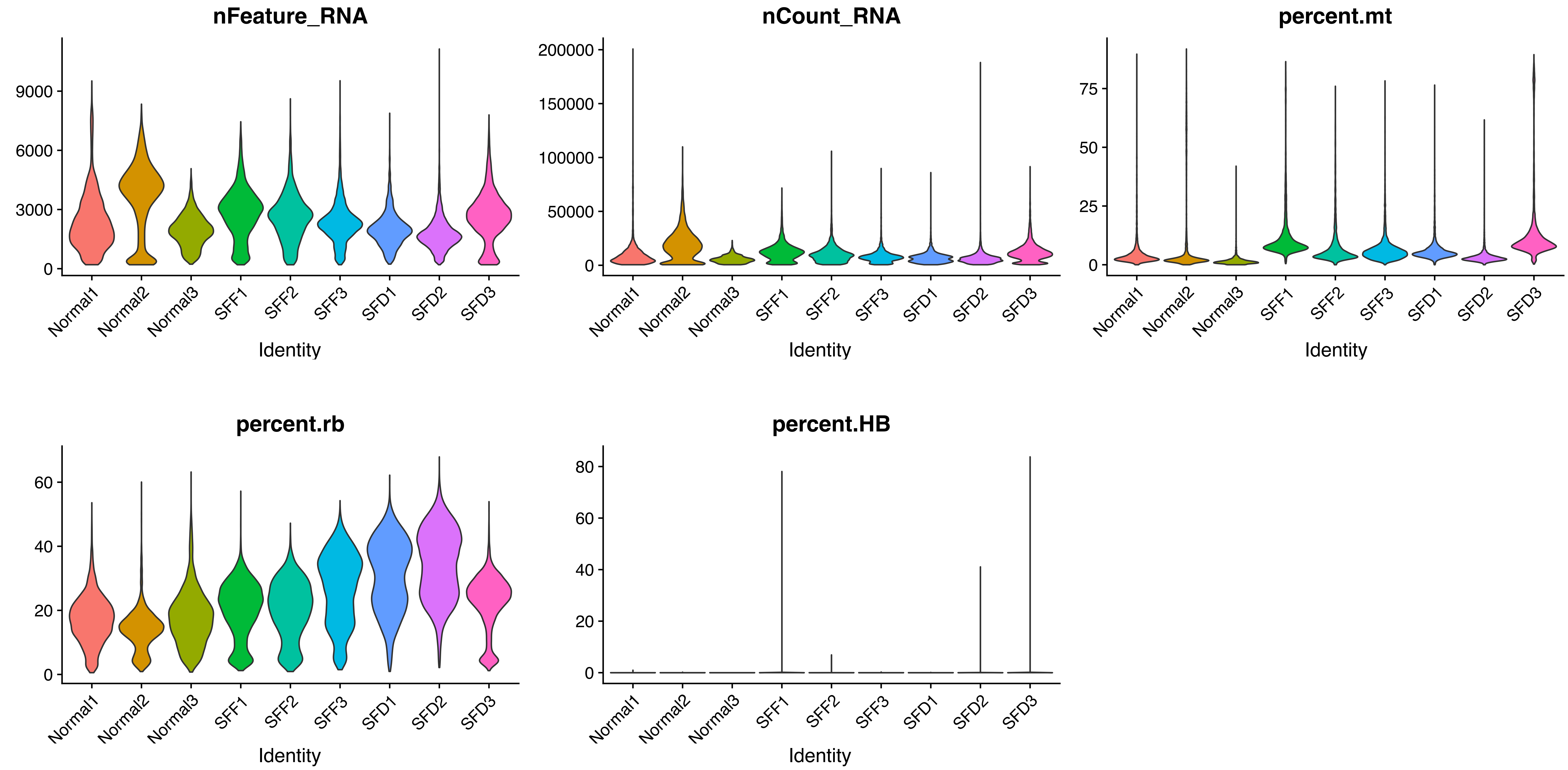

nFeature_RNA
nCount_RNA
percent.mt
percent.rb
percent.HB
Normal1
Normal2
Normal3
SFF1
SFF2
SFF3
SFD1
SFD2
SFD3
Identity

c

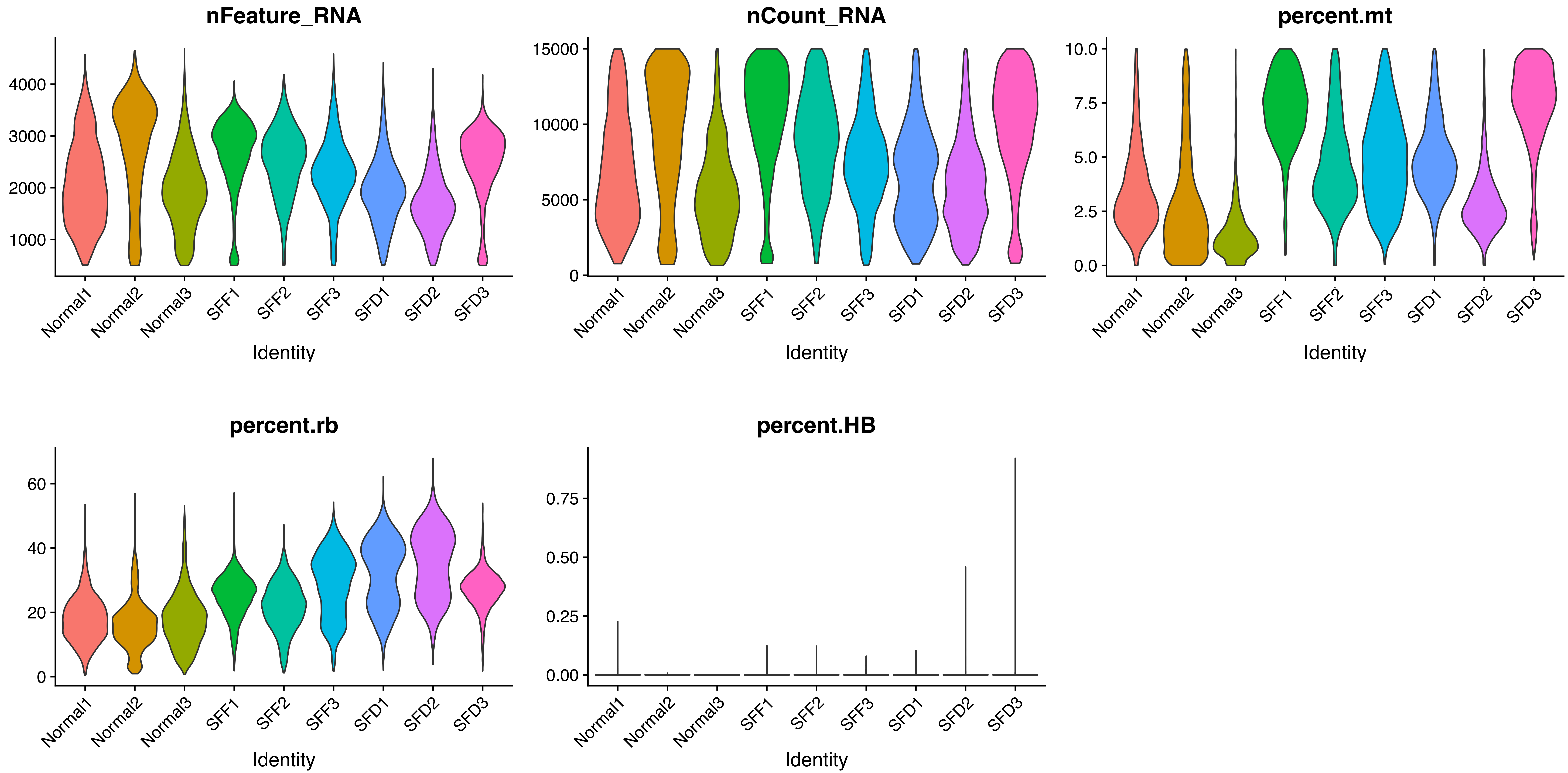

nFeature_RNA
nCount_RNA
percent.mt
percent.rb
percent.HB
4000
3000
2000
1000
15000
10000
5000
0
10.0
7.5
5.0
2.5
0.0
60
40
20
0
0.75
0.50
0.25
0.00
Normal1
Normal2
Normal3
SFF1
SFF2
SFF3
SFD1
SFD2
SFD3
Identity

d

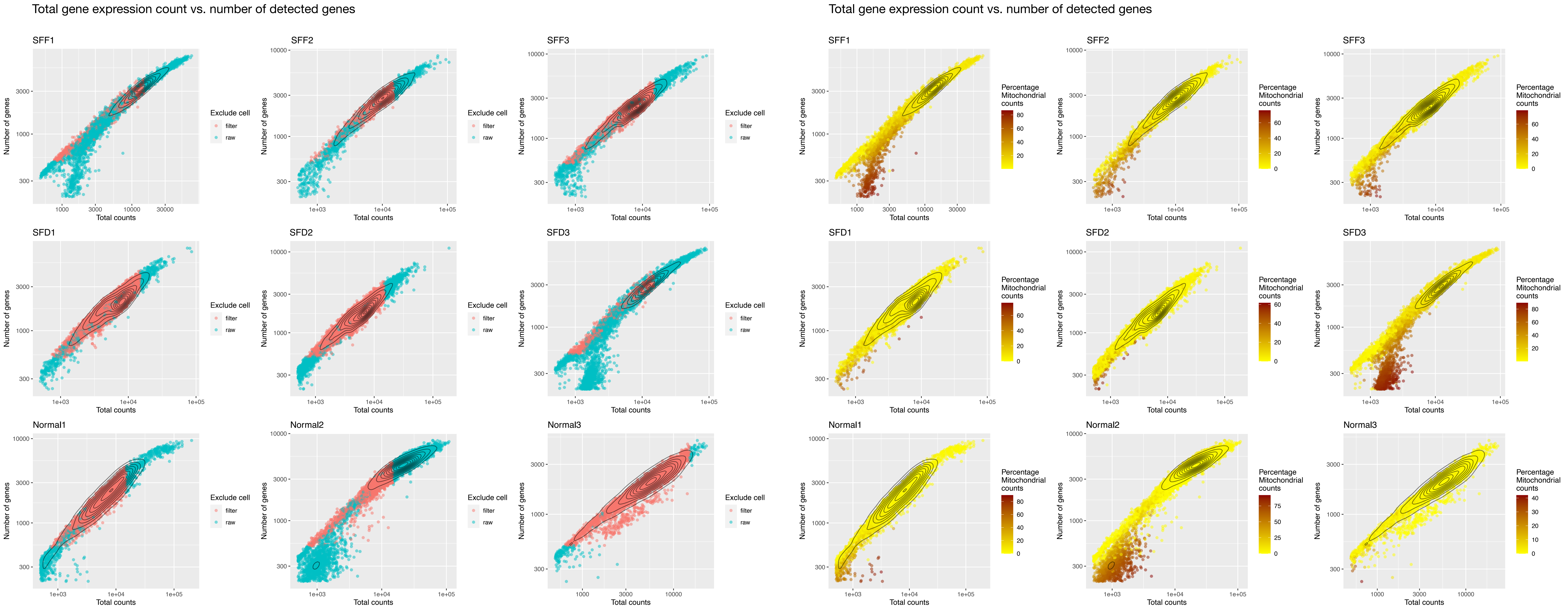

Total gene expression count vs. number of detected genes
SFF1
SFF2
SFF3
SFD1
SFD2
SFD3
Normal1
Normal2
Normal3
Number of genes
Total counts
Exclude cell
filter
raw
Total gene expression count vs. number of detected genes
SFF1
SFF2
SFF3
SFD1
SFD2
SFD3
Normal1
Normal2
Normal3
Number of genes
Total counts
Percentage Mitochondrial counts

e

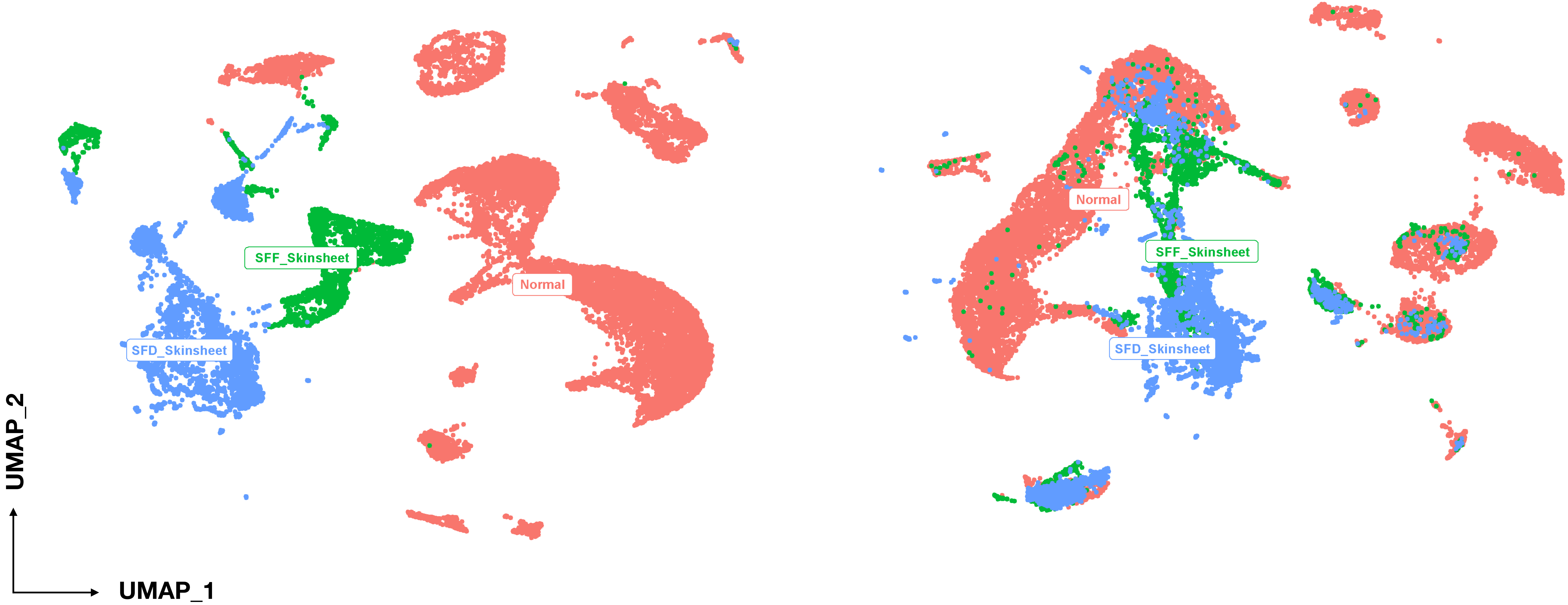
SFF_Skinsheet
SFD_Skinsheet
Normal
UMAP_2
UMAP_1
Normal
SFF_Skinsheet
SFD_Skinsheet

f

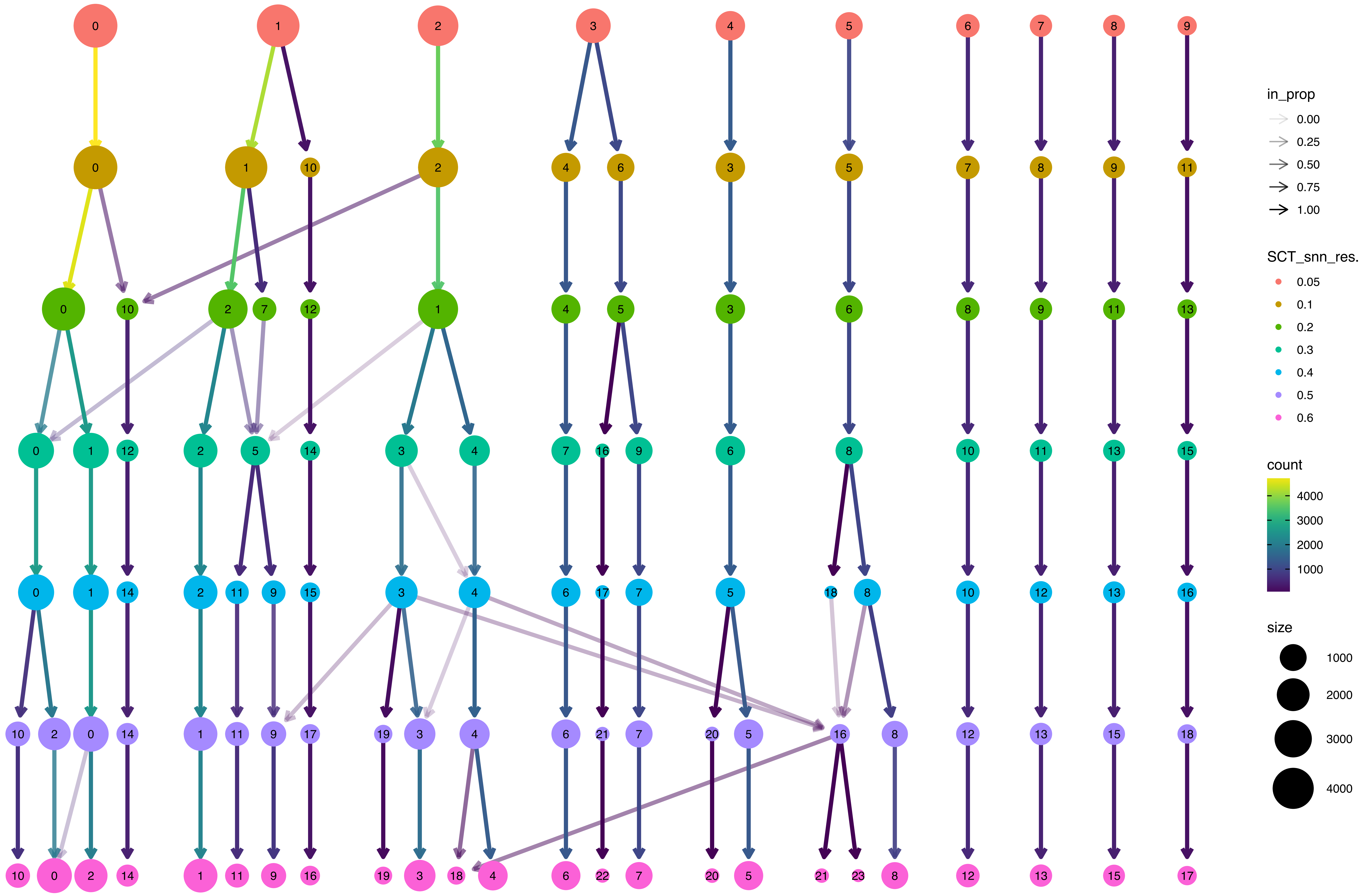
in_prop
0.00
0.25
0.50
0.75
1.00
SCT_snn_res.
0.05
0.1
0.2
0.3
0.4
0.5
0.6
count
4000
3000
2000
1000
size
1000
2000
3000
4000

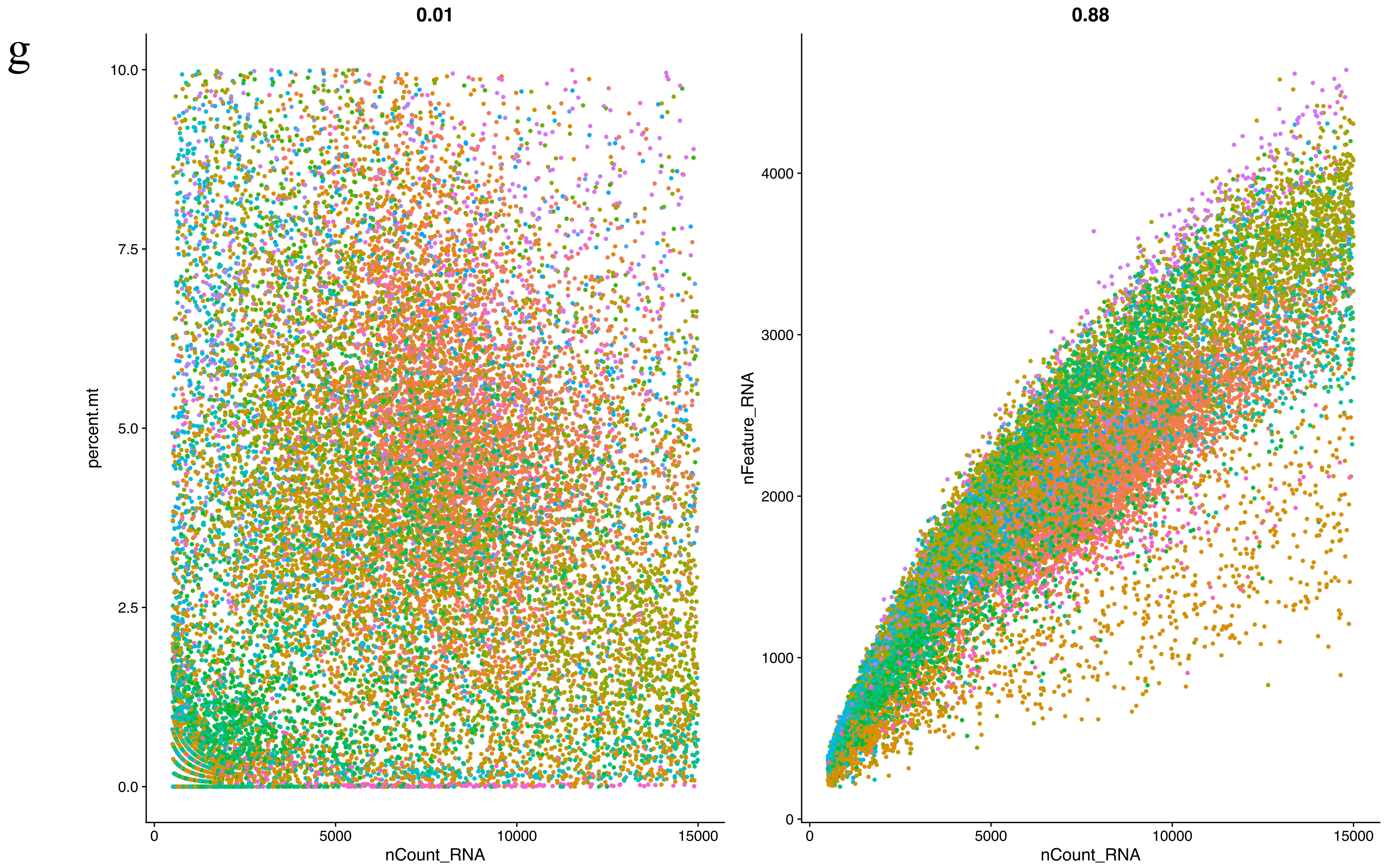
g
0.01
0.88
percent.mt
nFeature_RNA
nCount_RNA
nCount_RNA
10.0
7.5
5.0
2.5
0.0
4000
3000
2000
1000
0
0
5000
10000
15000
0
5000
10000
15000

h

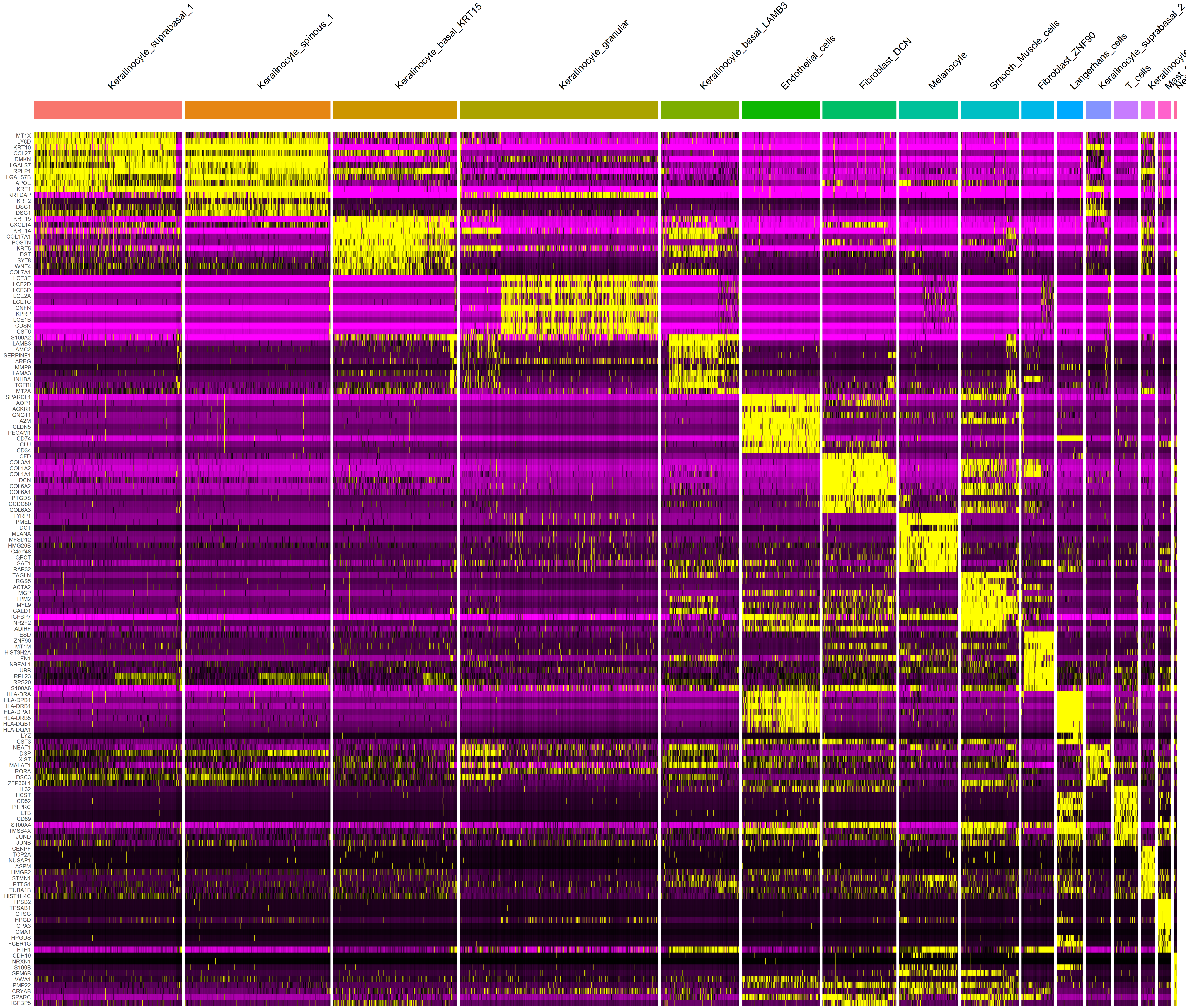
Keratinocyte_suprabasal_1
Keratinocyte_spinous_1
Keratinocyte_basal_KRT15
Keratinocyte_granular
Keratinocyte_basal_LAMB3
Endothelial_cells
Fibroblast_DCN
Melanocyte
Smooth_Muscle_cells
Fibroblast_ZNF90
Langerhans_cells
Keratinocyte_suprabasal_2
T_cells
MT1X
LY6D
KRT10
CCL27
DMKN
LGALS7
RPLP1
LGALS7B
APOE
KRT1
KRTDAP
KRT2
DSC1
DSG1
KRT15
CXCL14
KRT14
COL17A1
POSTN
KRT5
DST
SYT8
WNT4
COL7A1
LCE3E
LCE2D
LCE3D
LCE2A
LCE1C
CNFN
KPRP
LCE1B
CDSN
CST6
S100A2
LAMB3
LAMC2
SERPINE1
AREG
MMP9
LAMA3
INHBA
TGFBI
MT2A
SPARCL1
AQP1
ACKR1
GNG11
A2M
CLDN5
PECAM1
CD74
CLU
CD34
CFD
COL3A1
COL1A2
COL1A1
DCN
COL6A2
COL6A1
PTGDS
CCDC80
COL6A3
TYRP1
PMEL
DCT
MLANA
MFSD12
HMG20B
C4orf48
QPCT
SAT1
RAB32
TAGLN
RGS5
ACTA2
MGP
TPM2
MYL9
CALD1
IGFBP7
NR2F2
ADIRF
ESD
ZNF90
MT1M
HIST3H2A
FN1
NBEAL1
UBB
RPL23
RPS20
S100A6
HLA-DRA
HLA-DPB1
HLA-DRB1
HLA-DPA1
HLA-DRB5
HLA-DQB1
HLA-DQA1
LYZ
CST3
NEAT1
DSP
XIST
MALAT1
RORA
DSC3
ZFP36L1
IL32
HCST
CD52
PTPRC
LTB
CD69
S100A4
TMSB4X
JUND
JUNB
CENPF
TOP2A
NUSAP1
ASPM
HMGB2
STMN1
PTTG1
TUBA1B
HIST1H4C
TPSB2
TPSAB1
CTSG
HPGD
CPA3
CMA1
HPGDS
FCER1G
FTH1
CDH19
NRXN1
S100B
GPM6B
VWA1
PMP22
CRYAB
SPARC
IGFBP5

I

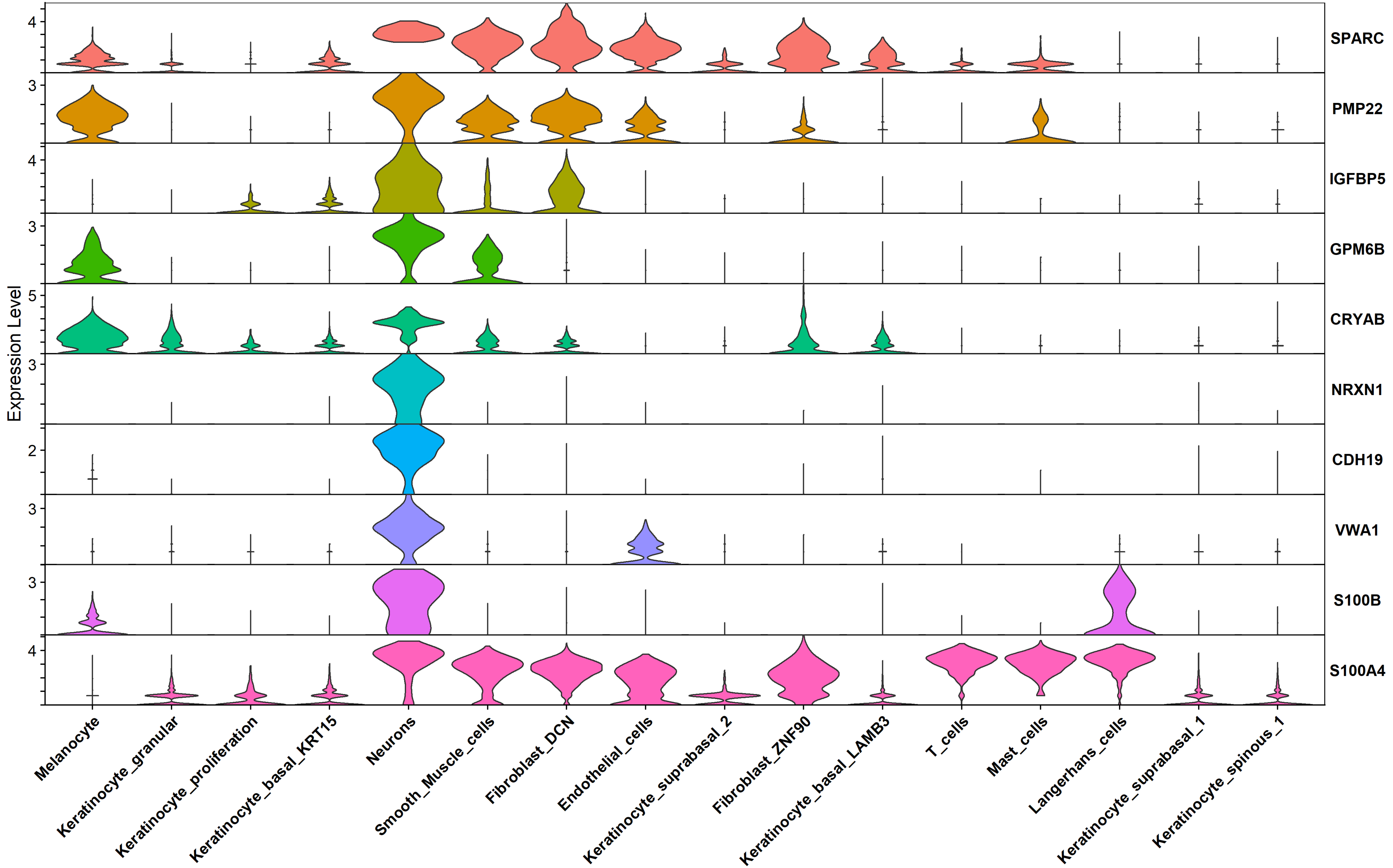

Expression Level
SPARC
PMP22
IGFBP5
GPM6B
CRYAB
NRXN1
CDH19
VWA1
S100B
S100A4
Melanocyte
Keratinocyte_granular
Keratinocyte_proliferation
Keratinocyte_basal_KRT15
Neurons
Smooth_Muscle_cells
Fibroblast_DCN
Endothelial_cells
Keratinocyte_suprabasal_2
Fibroblast_ZNF90
Keratinocyte_basal_LAMB3
T_cells
Mast_cells
Langerhans_cells
Keratinocyte_suprabasal_1
Keratinocyte_spinous_1

j

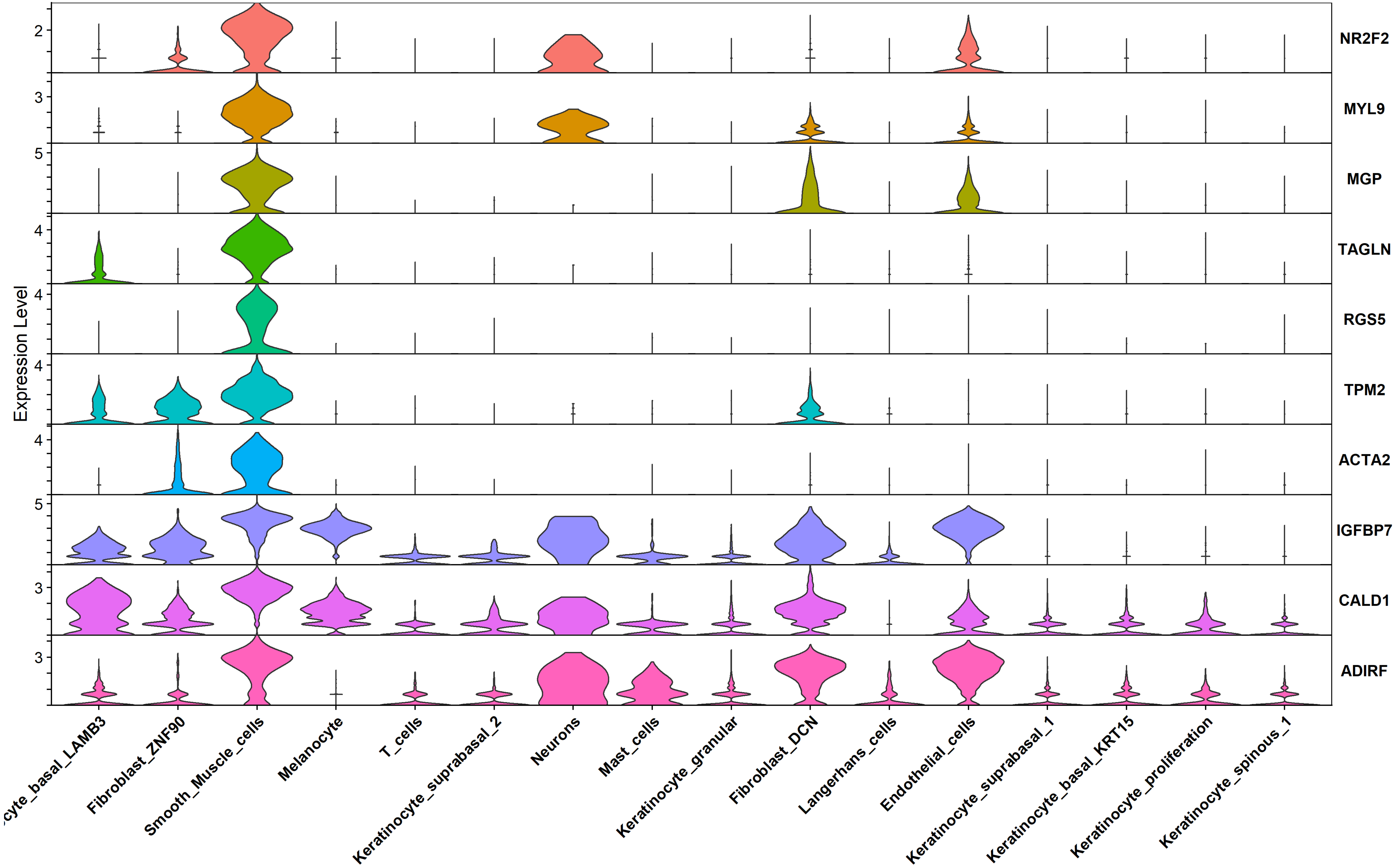
NR2F2
MYL9
MGP
TAGLN
RGS5
TPM2
ACTA2
IGFBP7
CALD1
ADIRF
Expression Level
2
3
5
4
4
4
4
5
3
3
cyte_basal_LAMB3
Fibroblast_ZNF90
Smooth_Muscle_cells
Melanocyte
T_cells
Keratinocyte_suprabasal_2
Neurons
Mast_cells
Keratinocyte_granular
Fibroblast_DCN
Langerhans_cells
Endothelial_cells
Keratinocyte_suprabasal_1
Keratinocyte_basal_KRT15
Keratinocyte_proliferation
Keratinocyte_spinous_1

k

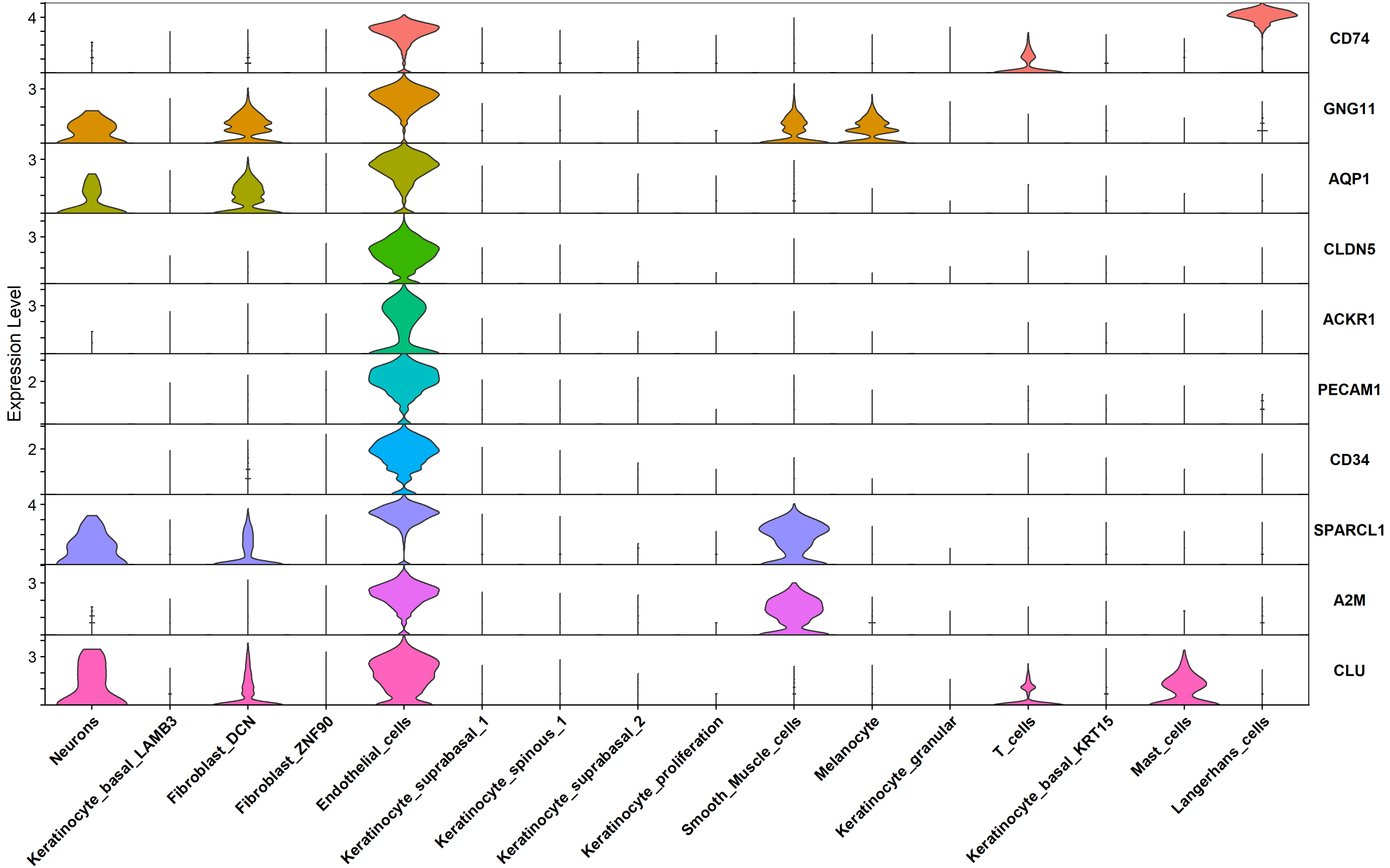
Expression Level
CD74
GNG11
AQP1
CLDN5
ACKR1
PECAM1
CD34
SPARCL1
A2M
CLU
Neurons
Keratinocyte_basal_LAMB3
Fibroblast_DCN
Fibroblast_ZNF90
Endothelial_cells
Keratinocyte_suprabasal_1
Keratinocyte_spinous_1
Keratinocyte_suprabasal_2
Keratinocyte_proliferation
Smooth_Muscle_cells
Melanocyte
Keratinocyte_granular
T_cells
Keratinocyte_basal_KRT15
Mast_cells
Langerhans_cells

1

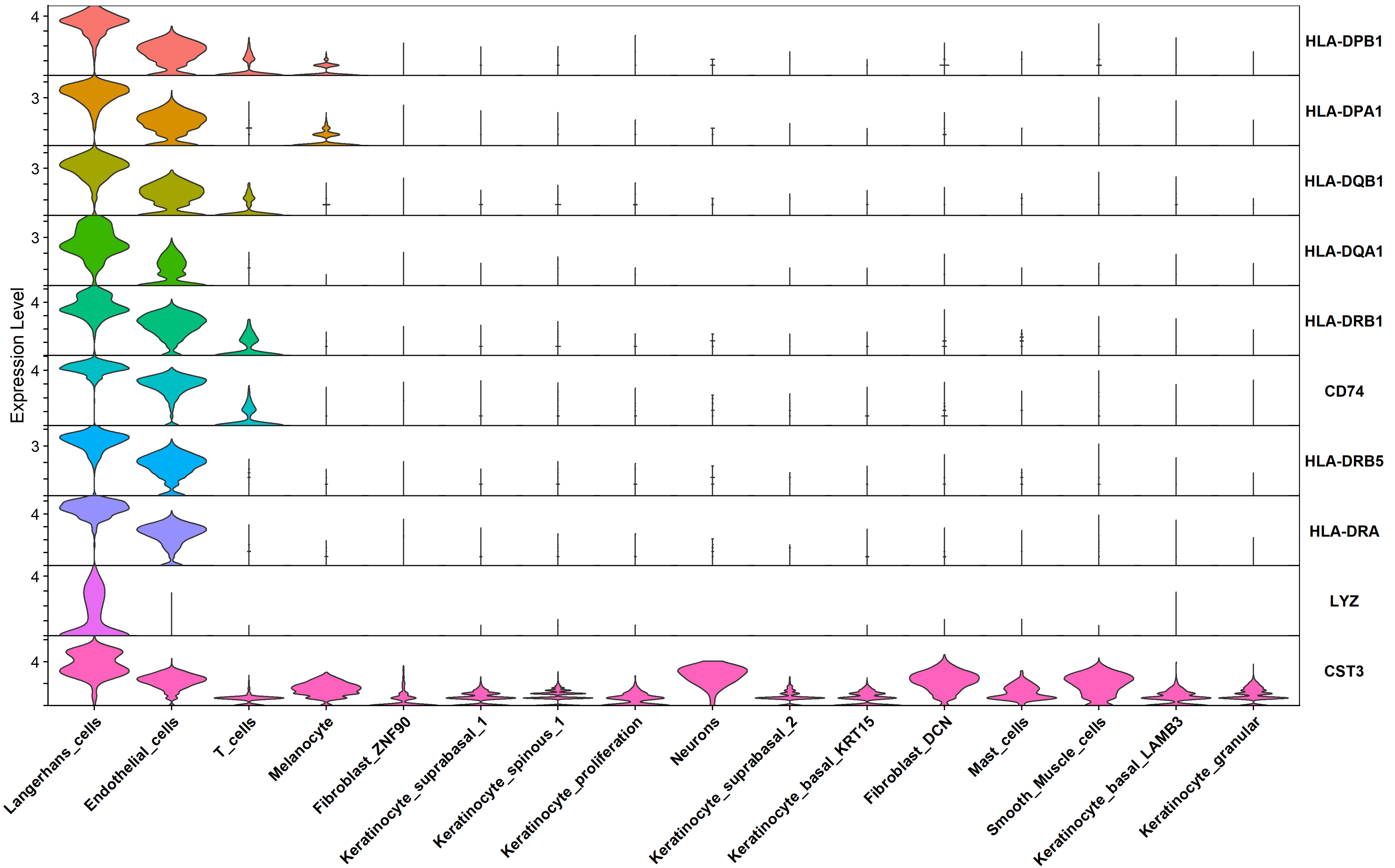
Expression Level
HLA-DPB1
HLA-DPA1
HLA-DQB1
HLA-DQA1
HLA-DRB1
CD74
HLA-DRB5
HLA-DRA
LYZ
CST3
Langerhans_cells
Endothelial_cells
T_cells
Melanocyte
Fibroblast_ZNF90
Keratinocyte_suprabasal_1
Keratinocyte_spinous_1
Keratinocyte_proliferation
Neurons
Keratinocyte_suprabasal_2
Keratinocyte_basal_KRT15
Fibroblast_DCN
Mast_cells
Smooth_Muscle_cells
Keratinocyte_basal_LAMB3
Keratinocyte_granular

m

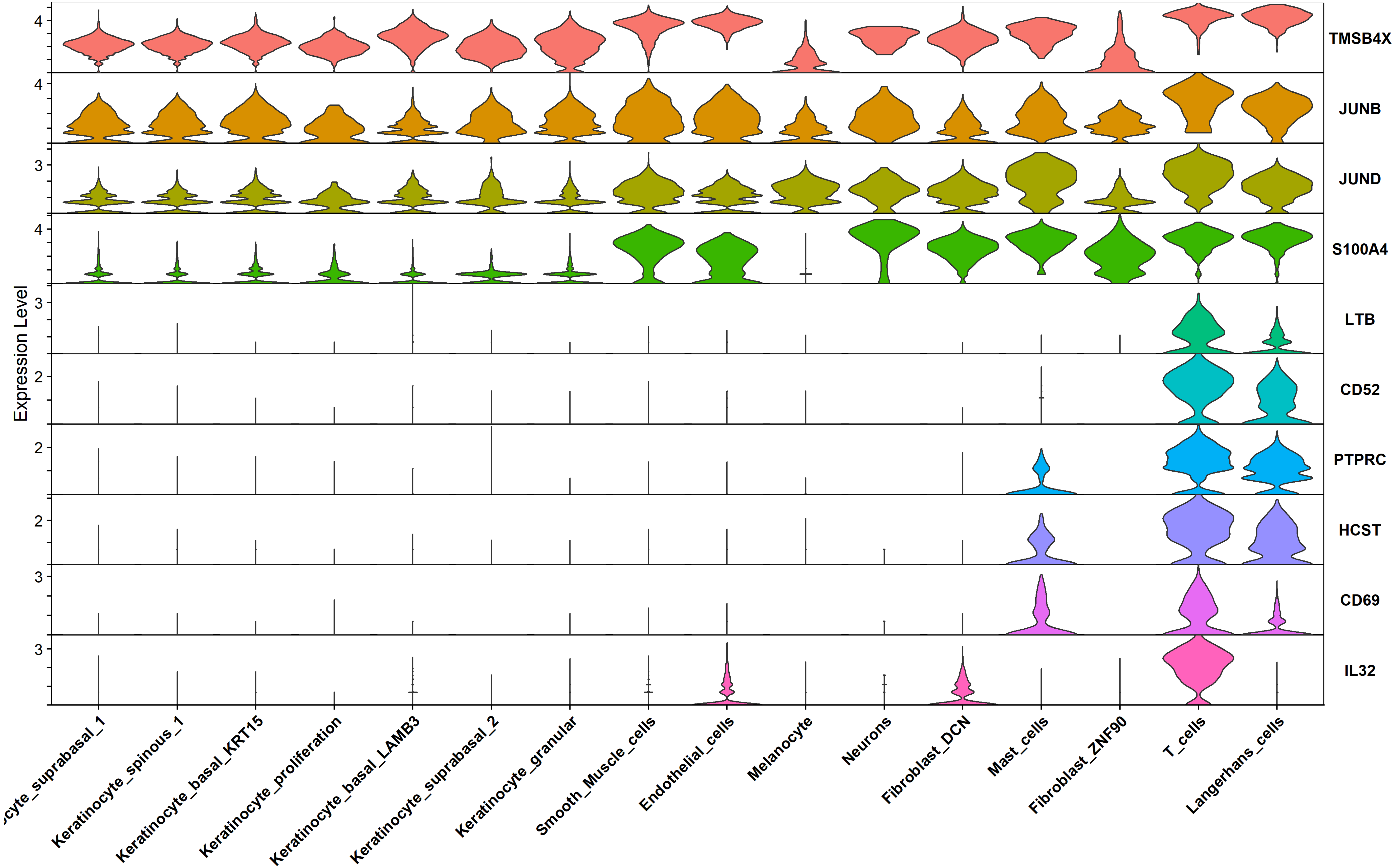

Expression Level
TMSB4X
JUNB
JUND
S100A4
LTB
CD52
PTPRC
HCST
CD69
IL32
4
4
3
4
3
2
2
2
3
3
cyte_suprabasal_1
Keratinocyte_spinous_1
Keratinocyte_basal_KRT15
Keratinocyte_proliferation
Keratinocyte_basal_LAMB3
Keratinocyte_suprabasal_2
Keratinocyte_granular
Smooth_Muscle_cells
Endothelial_cells
Melanocyte
Neurons
Fibroblast_DCN
Mast_cells
Fibroblast_ZNF90
T_cells
Langerhans_cells

n

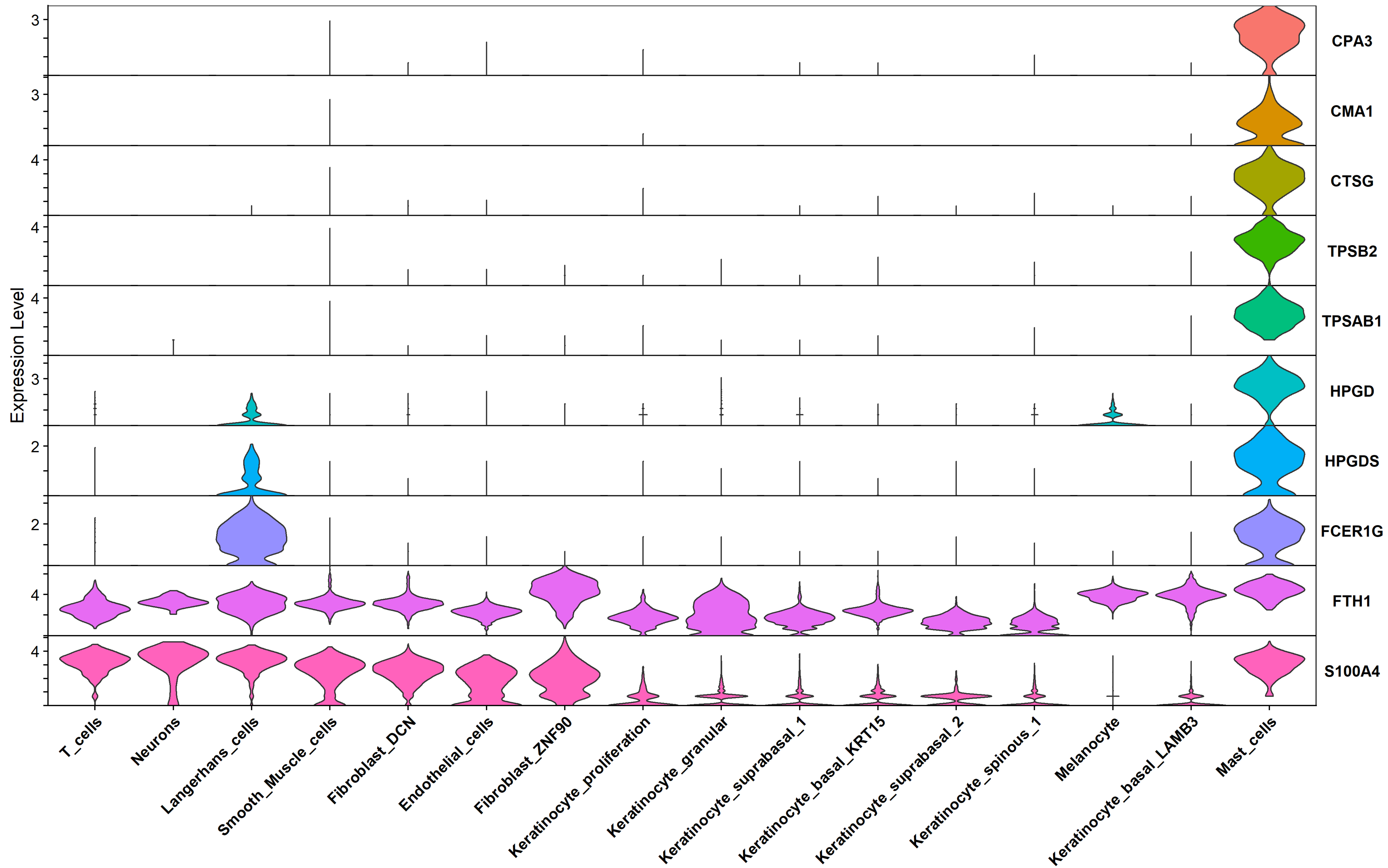

Expression Level
CPA3
CMA1
CTSG
TPSB2
TPSAB1
HPGD
HPGDS
FCER1G
FTH1
S100A4
T_cells
Neurons
Langerhans_cells
Smooth_Muscle_cells
Fibroblast_DCN
Endothelial_cells
Fibroblast_ZNF90
Keratinocyte_proliferation
Keratinocyte_granular
Keratinocyte_suprabasal_1
Keratinocyte_basal_KRT15
Keratinocyte_suprabasal_2
Keratinocyte_spinous_1
Melanocyte
Keratinocyte_basal_LAMB3
Mast_cells

Supplementary Fig. S2 Hierarchical clustering analysis of all cells acquired by scRNA-seq of normal skin of patients with vitiligo and their derived cultured epithelial sheets under SFF and SFD conditions. a Datasets of normal skin, SFD epithelial sheets, and SFD epithelial sheets from three patients with vitiligo sequenced. **b, c** Violin plots showing the count of feature genes, the percentage of mitochondrial transcripts, and the percentage of red blood cells of specific marker genes for before (b) and after (c) the quality control. **d** Quality control of scRNA sequencing analysis of 9 samples. **e** UMAP (uniform manifold approximation and projection) plot of single cells derived from 3 groups of samples before (left) and after (right) the quality control. **f** The Clustree plot for hierarchical clustering. The fourth row illustrates the best clustering, which representing 16 distinct cell clusters. **g** Scatter plots of principal component analysis (PCA) that reduced the dimensionality of the data. **h** Heatmap showing the expression of all genes in the 16 cell clusters. Each column represents a cluster and each row represents a specific gene. Yellow indicates maximum gene expression while purple indicates no expression in scaled log-normalized UMI counts. **i-n** Violin plots showing the expression of specific marker genes for neurons(i), smooth muscle cells (j), endothelial cells (k), Langerhans cells (l), T cells (m), and mast cells (n).

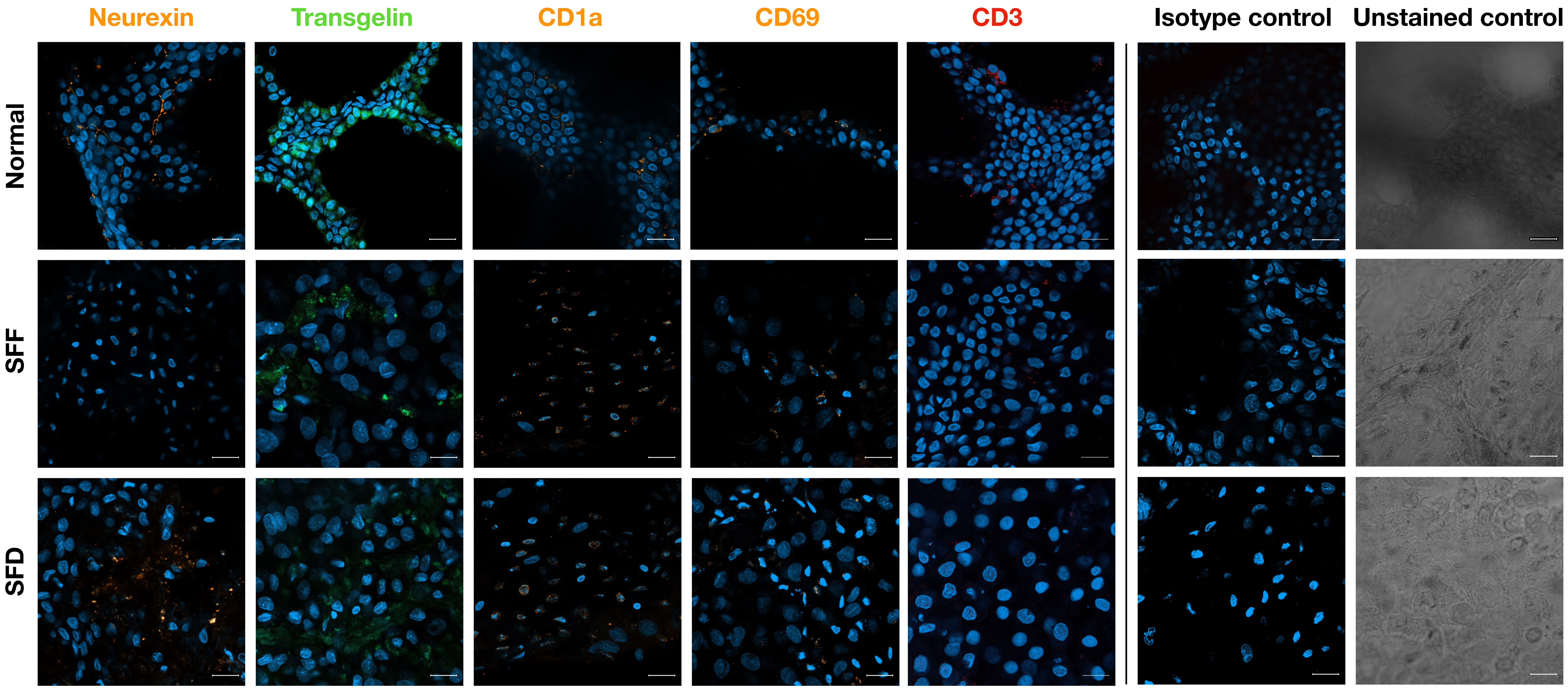

Supplementary Fig. S3 Cell-specific marker genes identifying the cell clusters. Immunofluorescence staining of neurexin (orange) for neurons, transgelin (green) for smooth muscle cells and pericytes, CD1a (orange) for Langerhans cells, CD69 (orange), CD3 (red), IgG isotype control, and unstained control for T cells in SFF epithelium, SFD epithelium and normal skin (HOECHST with light blue for nucleus). Scale bar = 50 μm.

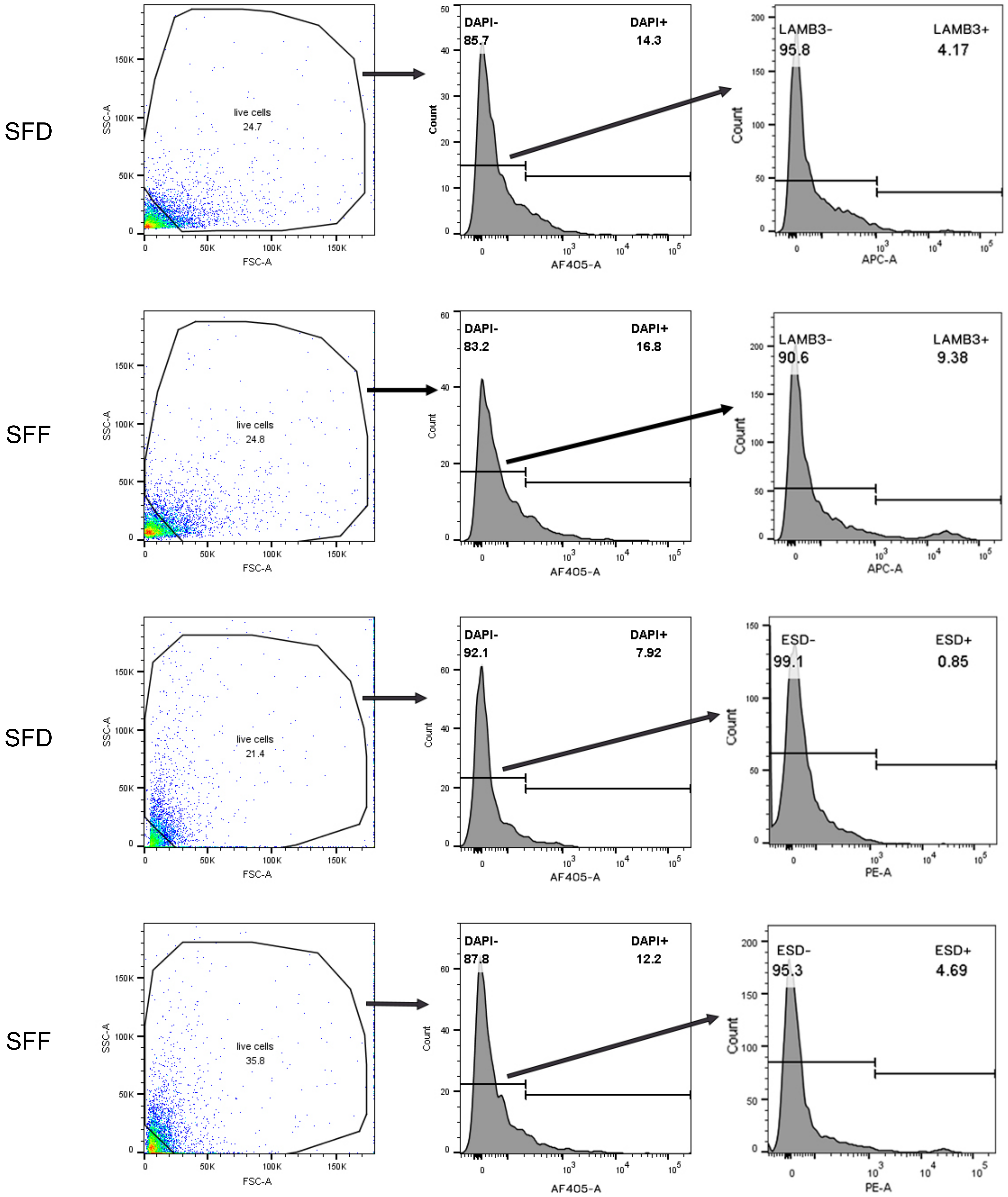

Supplementary Fig. S4 Gating strategy detecting the proportion of LAMB3+ cells and ESD+ cells in cultured epithelial sheet. Subpopulations gated manually, followed by DAPI fluorescence to distinguish between live and dead cells, thereby determining the proportion of target cells .

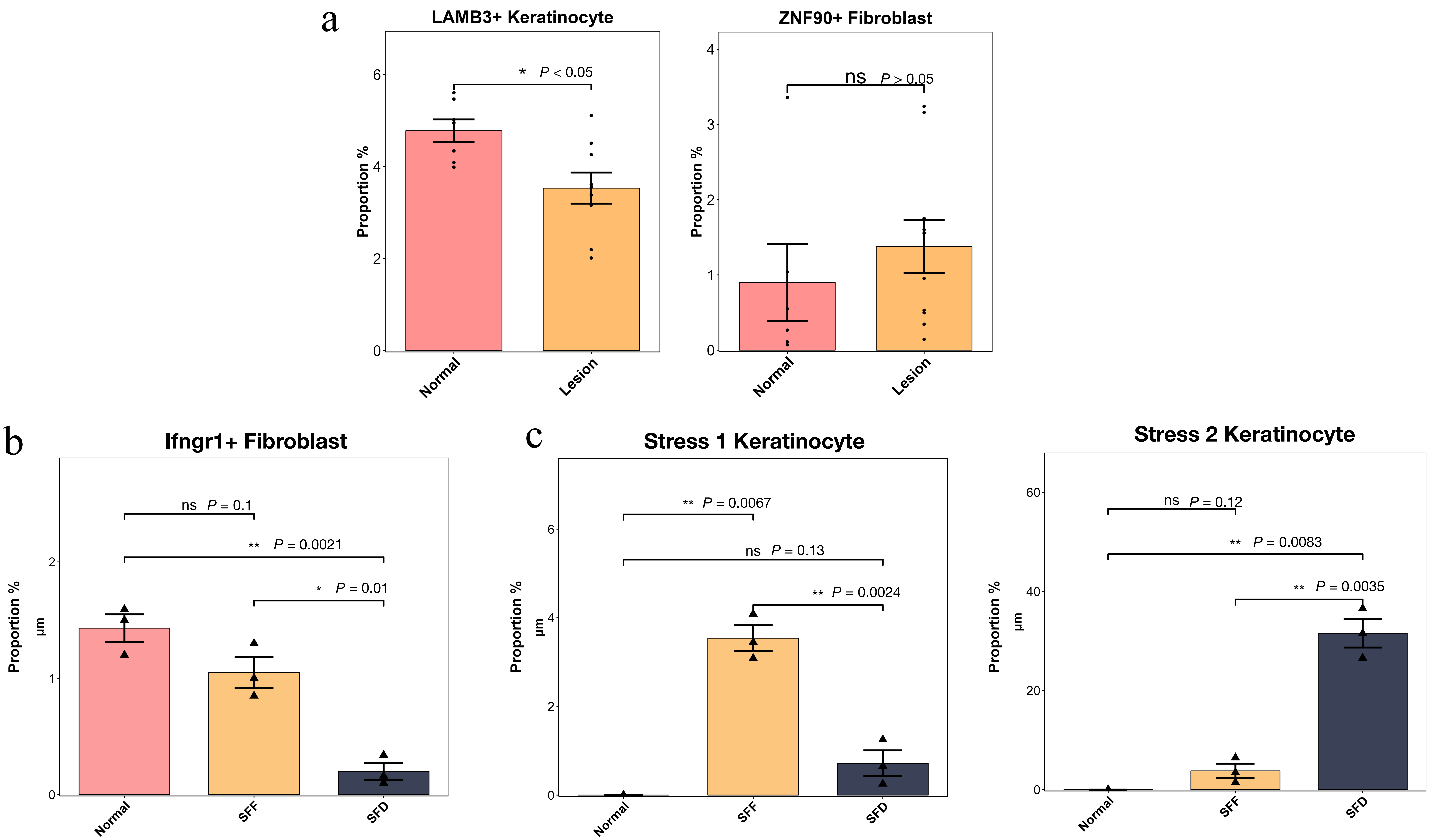
a
LAMB3+ Keratinocyte
Proportion %
* P < 0.05
Normal
Lesion
ZNF90+ Fibroblast
Proportion %
ns P > 0.05
Normal
Lesion
b
Ifngr1+ Fibroblast
Proportion %
μm
ns P = 0.1
** P = 0.0021
* P = 0.01
Normal
SFF
SFD
c
Stress 1 Keratinocyte
Proportion %
μm
** P = 0.0067
ns P = 0.13
** P = 0.0024
Normal
SFF
SFD
Stress 2 Keratinocyte
Proportion %
μm
ns P = 0.12
** P = 0.0083
** P = 0.0035
Normal
SFF
SFD

Supplementary Fig. S5 Integrative analysis of scRNA-seq data from vitiligo skin and healthy skin. a Proportions of LAMB3+ basal keratinocytes and ZNF90+ fibroblasts in the normal skin and vitiligo lesion **(**Data published by Xu et.al). **b** Proportions of Ifngr1+ fibroblasts related to vitiligo immune response in normal skin, SFF, and SFD in this dataset **(**Data published by Xu et.al). **c** Proportions of vitiligo immune response-related keratinocytes in normal skin, SFF, and SFD in this dataset. Stress 1 subpopulation is indicated as KRT6A+high/KRT16+/S100A8/9+ (left), Stress 2 subpopulation is indicated as KRT6A+low/KRT16+/S100A8/9+ (right) **(**Data published by Shiu et.al). Biologically independent samples presented as individual dots. Data presented as the mean ± SD, analyzed with unpaired, nonparametric t-test. (*$P < 0.05$, ***$P < 0.01$)

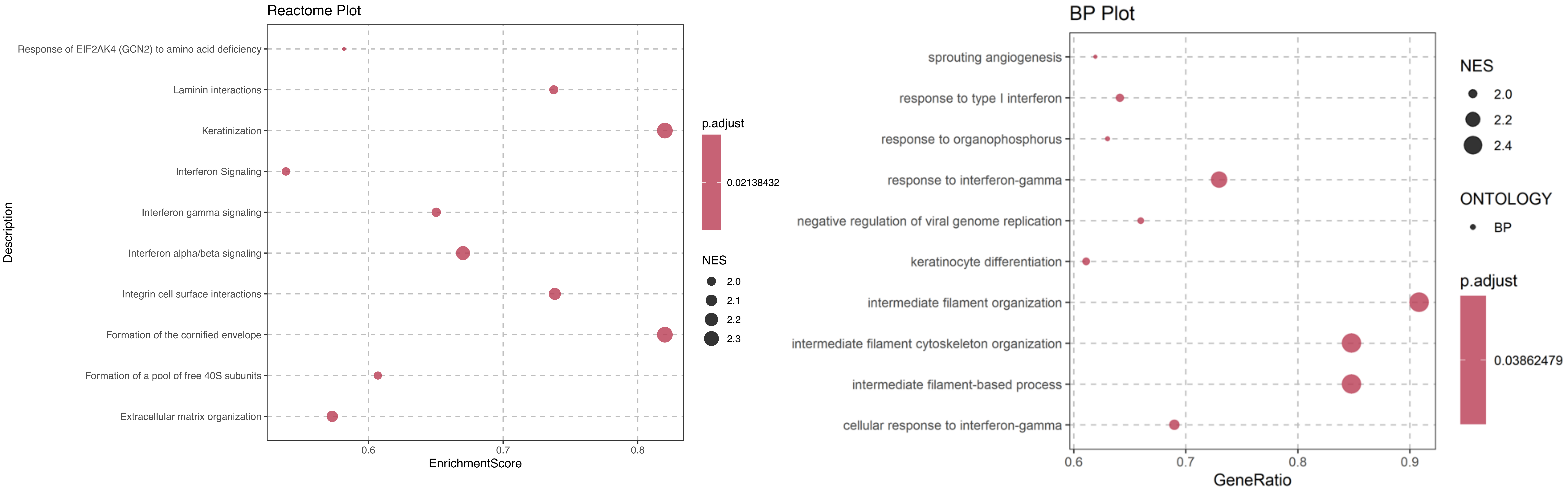

Supplementary Fig. S6 GSVA (gene set variation analysis) of shallow trancriptomes of sequenced melanocytes between SFF and SFD epithelial sheets. Enrichment analysis using Reacome (left) and GO (right) database showed no correlation in melanocyte-related pathways.

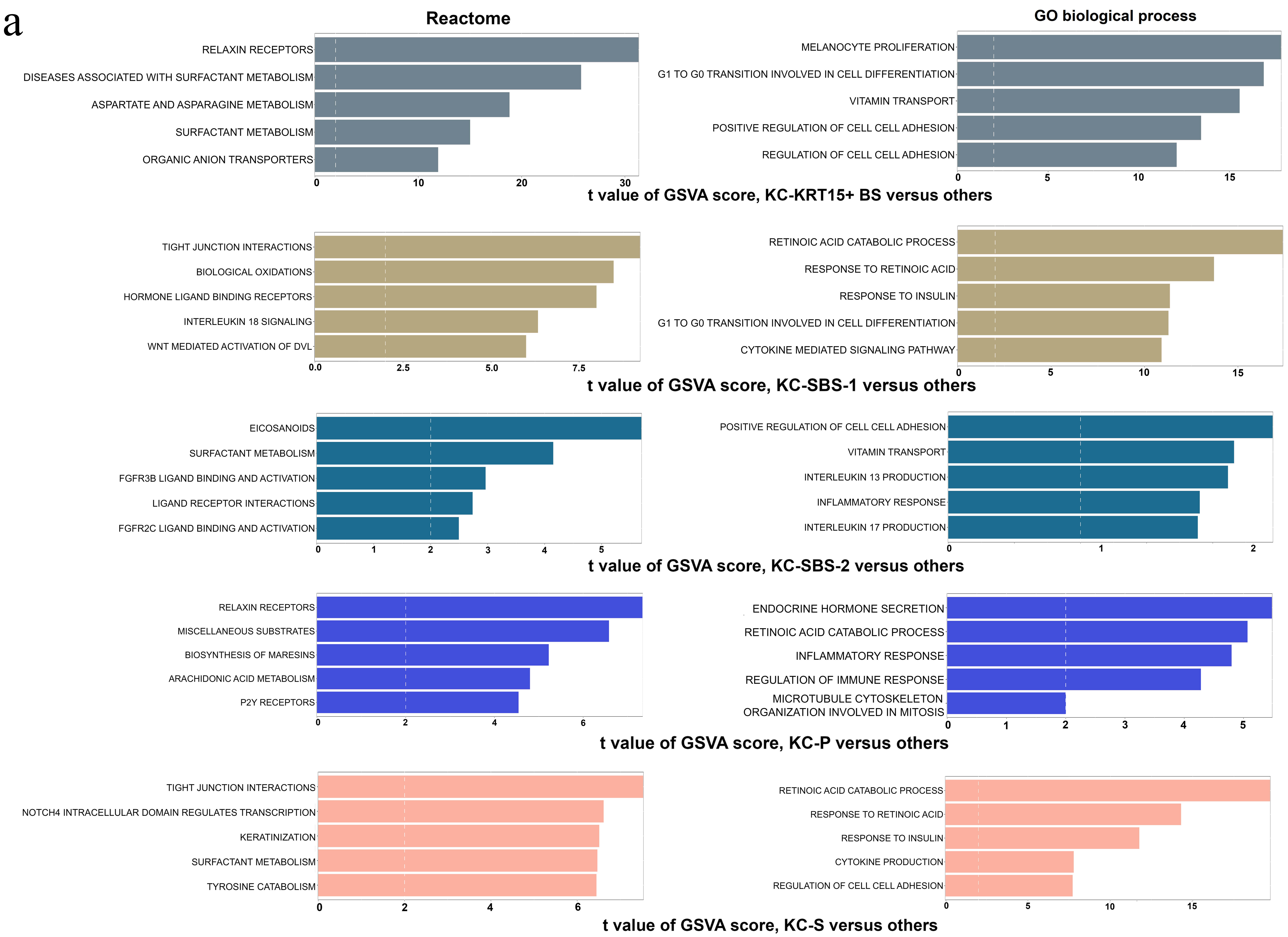
a
Reactome
GO biological process
RELAXIN RECEPTORS
DISEASES ASSOCIATED WITH SURFACTANT METABOLISM
ASPARTATE AND ASPARAGINE METABOLISM
SURFACTANT METABOLISM
ORGANIC ANION TRANSPORTERS
MELANOCYTE PROLIFERATION
G1 TO G0 TRANSITION INVOLVED IN CELL DIFFERENTIATION
VITAMIN TRANSPORT
POSITIVE REGULATION OF CELL CELL ADHESION
REGULATION OF CELL CELL ADHESION
t value of GSVA score, KC-KRT15+ BS versus others
TIGHT JUNCTION INTERACTIONS
BIOLOGICAL OXIDATIONS
HORMONE LIGAND BINDING RECEPTORS
INTERLEUKIN 18 SIGNALING
WNT MEDIATED ACTIVATION OF DVL
RETINOIC ACID CATABOLIC PROCESS
RESPONSE TO RETINOIC ACID
RESPONSE TO INSULIN
G1 TO G0 TRANSITION INVOLVED IN CELL DIFFERENTIATION
CYTOKINE MEDIATED SIGNALING PATHWAY
t value of GSVA score, KC-SBS-1 versus others
EICOSANOIDS
SURFACTANT METABOLISM
FGFR3B LIGAND BINDING AND ACTIVATION
LIGAND RECEPTOR INTERACTIONS
FGFR2C LIGAND BINDING AND ACTIVATION
POSITIVE REGULATION OF CELL CELL ADHESION
VITAMIN TRANSPORT
INTERLEUKIN 13 PRODUCTION
INFLAMMATORY RESPONSE
INTERLEUKIN 17 PRODUCTION
t value of GSVA score, KC-SBS-2 versus others
RELAXIN RECEPTORS
MISCELLANEOUS SUBSTRATES
BIOSYNTHESIS OF MARESINS
ARACHIDONIC ACID METABOLISM
P2Y RECEPTORS
ENDOCRINE HORMONE SECRETION
RETINOIC ACID CATABOLIC PROCESS
INFLAMMATORY RESPONSE
REGULATION OF IMMUNE RESPONSE
MICROTUBULE CYTOSKELETON ORGANIZATION INVOLVED IN MITOSIS
t value of GSVA score, KC-P versus others
TIGHT JUNCTION INTERACTIONS
NOTCH4 INTRACELLULAR DOMAIN REGULATES TRANSCRIPTION
KERATINIZATION
SURFACTANT METABOLISM
TYROSINE CATABOLISM
RETINOIC ACID CATABOLIC PROCESS
RESPONSE TO RETINOIC ACID
RESPONSE TO INSULIN
CYTOKINE PRODUCTION
REGULATION OF CELL CELL ADHESION
t value of GSVA score, KC-S versus others

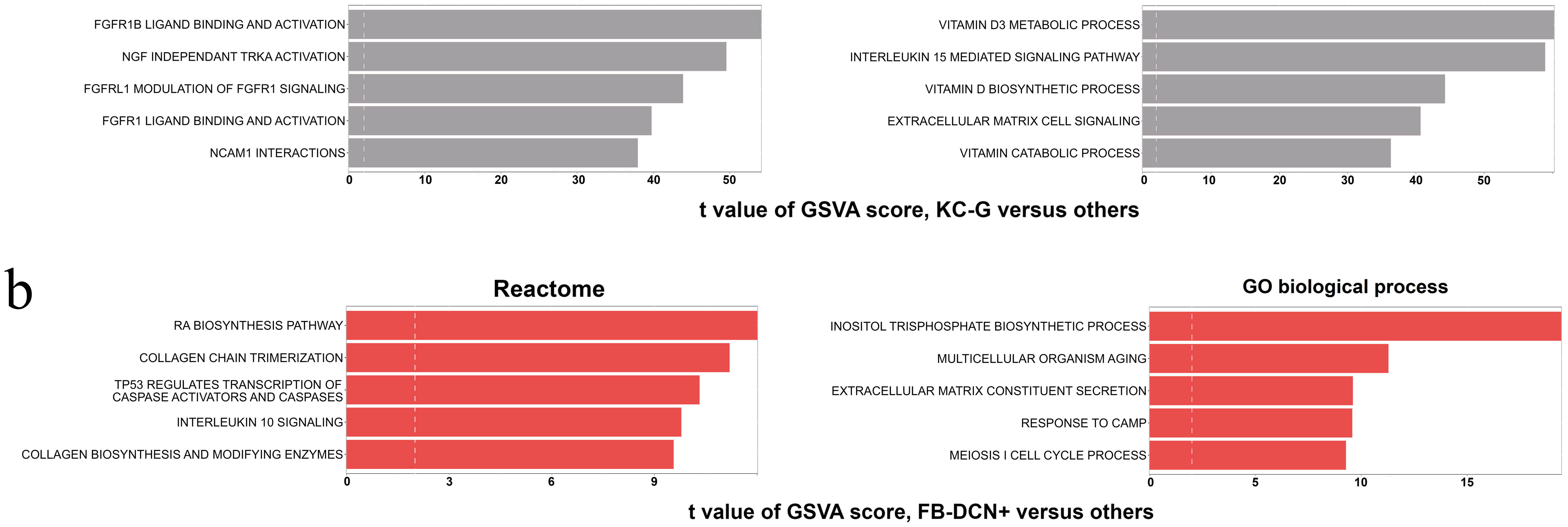

Supplementary Fig. S7 GSVA of other keratinocytes and fibroblasts except for LAMB3+ basal keratinocytes and ZNF90+ fibroblasts. a Bar plot showing the upregulated Reactome (left) and GO (right) pathways between keratinocyte subtypes (KRT15+ basal keratinocytes, suprabasal keratinocytes 1, suprabasal keratinocytes 2, proliferating keratinocytes, spinous keratinocytes, granular keratinocytes) and other keratinocytes. b Bar plot showing the upregulated Reactome (left) and GO (right) pathways between DCN+ fibroblasts and other fibroblasts. Shown are t values from a linear model.

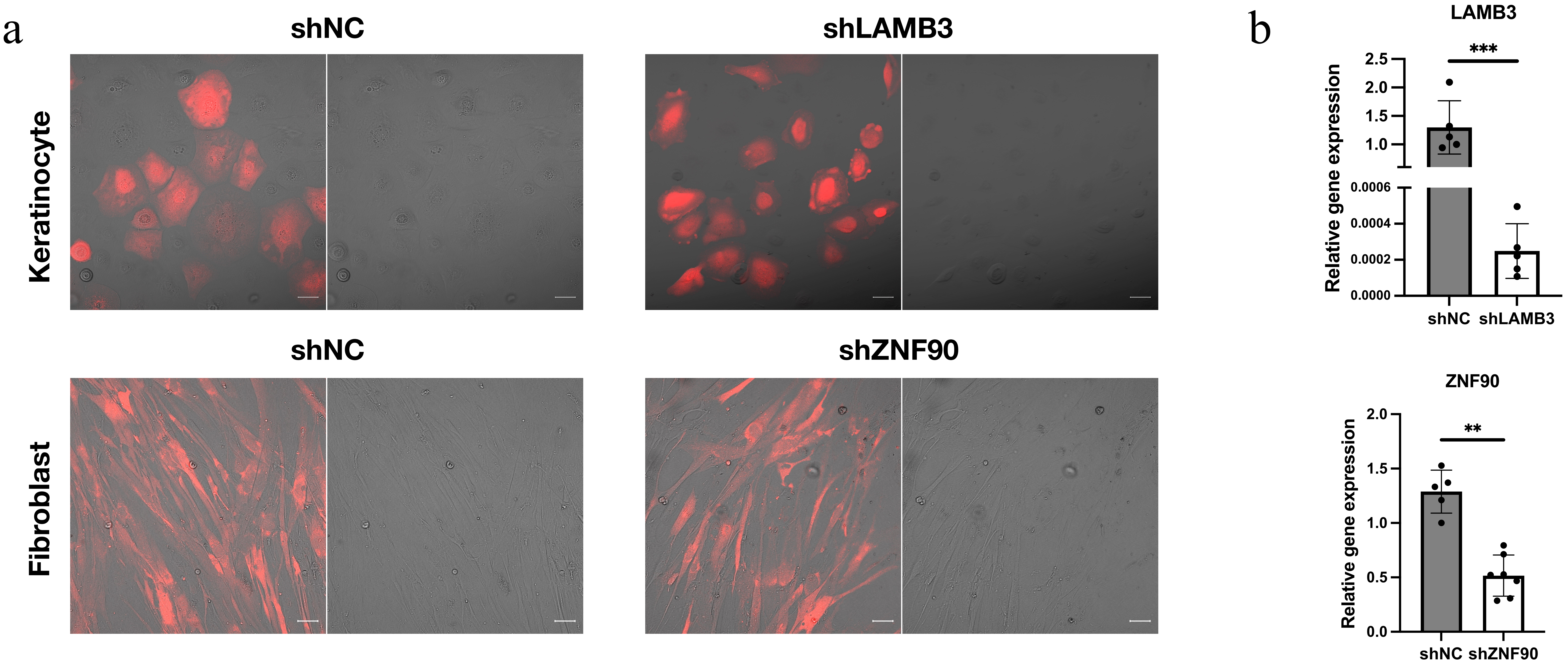

Supplementary Fig. S8 Knockdown of LAMB3 in keratinocytes and ZNF90 in fibroblasts. a Keratinocytes and fibroblasts infected with lentivirus for 72 hours, followed by puromycin selection for one week. The images represent a combination of fluorescence channels and bright-field microscopy. The red color represents mCherry fluorescence. Scale bar = 50 µm. **b** qPCR results display the expression levels of *LAMB3* in keratinocytes for the *LAMB3* knockdown group (shLAMB3) and the control group (ShNC), as well as the expression levels of *ZNF90* in fibroblasts for the *ZNF90* knockdown group (shZNF90) and the control group (ShNC). For qPCR, n = 5-7 independent experiments conducted over two biologically independent patient-derived samples. Data presented as the mean ± SD, analyzed with unpaired, nonparametric t-test. ($^{**}P < 0.01$, $^{***}P < 0.001$)

Supplementary Table. S1. The list of antibodies used for immunofluorescence

Name	Brand	Cat.No	Host
Cytokeratin 5	abcam	ab52635	Rabbit
Cytokeration 16	abcam	ab76416	Rabbit
TYRP1	Abcam	ab170905	Rabbit
Cytokeratin 14 (LL001)	Santa Cruz	sc-53253	Mouse
Cytokeratin 6	Santa Cruz	sc-514520	Mouse
Vimentin	abcam	AB8978	Mouse
c-Kit	Santa Cruz	sc-13508	Mouse
Neurexin Iα (17)	Santa Cruz	sc-136001	Mouse
Transgelin	Santa Cruz	sc-53932 AF488	Mouse
CD69	Santa Cruz	sc-373799	Mouse
CD1a	abcam	ab201337	Mouse
CD3	Proteintech	17617-1-AP	Rabbit
PMEL	Thermo	RH2246132	Goat
MelanA	abcam	ab210546	Rabbit
LAMB3	Santa Cruz	sc-133178	LAMB3
ESD	abcam	ab131211	Rabbit
Anti-Mouse IgG H&L (Alexa Fluor® 555)	abcam	ab150110	Donkey
Anti-Rabbit IgG H&L (Alexa Fluor® 647)	abcam	ab150075	Donkey
Anti-Goat IgG H&L (Alexa Fluor® 488)	abcam	ab150129	Donkey
Hoechst 33342 Staining Dye Solution	abcam	ab228551	-

Supplementary Table. S2. The list of primers used for qPCR

Gene	Primer pair	
GAPDH	Forward	TGTGGGCATCAATGGATTTGG
	Reverse	ACACCATGTATTCCGGGTCAAT
LAMB3	Forward	GACTTCGGTAAGACCTGGCG
	Reverse	CTCTCAAGTTTGTGATCTCCCC
ZNF90	Forward	TGCTTCGTGTCTTCTTCTCCAG
	Reverse	TGGTCCCATTTCTAGGCTTCC
MIF	Forward	AGAACCGCTCCTACAGCAAG
	Reverse	GCGAAGGTGGAGTTGTTCCA
NOTCH1	Forward	GAGGCGTGGCAGACTATGC
	Reverse	CTTGTACTCCGTCAGCGTGA
NOTCH2	Forward	GATCACCCGAATGGCTATGAAT
	Reverse	GGGGTCACAGTTGTCAATGTT
JAG1	Forward	GGGGCAACACCTTCAACCTC
	Reverse	CCAGGCGAAACTGAAAGGC
NRP2	Forward	GCTGGCTATATCACCTCTCCC
	Reverse	TCTCGATTTCAAAGTGAGGGTTG
EGF	Forward	TGTCCACGCAATGTGTCTGAA
	Reverse	CATTATCGGGTGAGGAACAACC
