# Supplementary material for: Single-cell sequencing reveals increased LAMB3-positive basal keratinocytes and ZNF90-positive fibroblasts in autologous cultured epithelium
Source: Commun Biol. 2024 Jan 10;7:79. doi: 10.1038/s42003-023-05747-5 (PMC10781733; doi:10.1038/s42003-023-05747-5)
Supplement: Supplementary file 3 — Description of Additional Supplementary Files [file 42003_2023_5747_MOESM3_ESM.pdf]

Description of Additional Supplementary Files

File name: Supplementary Data 1
Description: GSVA and GSEA results of sequenced melanocytes compared SFF to SFD epithelial sheets.

File name: Supplementary Data 2
Description: GSVA results of LAMB3+ basal keratinocytes and other keratinocytes, and ZNF90+ fibroblasts and other fibroblasts.

File name: Supplementary Data 3
Description: Ligand-receptor interactions of all cell cluster pairings in SFF and SFD epithelial sheets.

File name: Supplementary Data 4
Description: Clinical research design, Ethics Committee approval, and informed consent templates.

File name: Supplementary Data 5
Description: Details of scRNA sequencing method.

File name: Supplementary Data 6
Description: Details of bioinformatic analysis.

File name: Supplementary Data 7
Description: The source data behind the graphs in the paper.
