# Supplementary material for: Single-cell sequencing reveals increased LAMB3-positive basal keratinocytes and ZNF90-positive fibroblasts in autologous cultured epithelium
Source: Commun Biol. 2024 Jan 10;7:79. doi: 10.1038/s42003-023-05747-5 (PMC10781733; doi:10.1038/s42003-023-05747-5)
Supplement: Supplementary file 7 — Supplementary Data 4 [file 42003_2023_5747_MOESM7_ESM.docx]

**Protocol: KY2020-698**

**1 Title: Retrospective study of autologous cultured epithelium grafting in the treatment of vitiligo**

**2 Purpose**

The efficacy and safety of autologous cultured epithelium grafting in the treatment of stable vitiligo were retrospectively summarized according to the existing clinical data;

**3 Study background**:

**3.1 Overview and main treatment methods of vitiligo**

Vitiligo is a common disease in Department of dermatology outpatient clinic. The incidence rate of 0.4-4% in Shanghai (0.54% in the area) has been increasing in recent years. This disease affects the patient's appearance and causes great psychological trauma to the patient. Because of its complex etiology, difficult treatment and long course of treatment, it has brought great psychological and economic burden to patients. With the improvement of people's living standards and medical development, patients with vitiligo have greater and greater expectations for treatment, which requires medical workers to provide better and effective treatment methods.

**3.2 Current domestic surgical methods and advantages of** **autologous cultured epithelium grafting in the treatment of vitiligo**

At present, the most efficient surgical treatment methods in China are suction blister epidermal grafting, thick skin graft, thin skin graft, microporous transplantation, etc., but they have high requirements for the skin donor area of patients, and it is difficult to transplant in a large area. These conventional surgical interventions usually have the disadvantage of uneven pigment after transplantation. In addition, for patients with generalized vitiligo, especially those who are ineffective in other treatment methods such as phototherapy and topical ointment, the treatment is more difficult. The donor area is greatly limited, and it cannot provide sufficient healthy epidermis, which limits the use of currently used surgical methods such as suction blister epidermal grafting.

Epidermal cell culture technology was first established by Rheinwald and Gree in 1975. With the time passing by, cultured epidermis sheet and cell molecular biology have developed rapidly, and the basic research and clinical application of epidermal cell culture and transplantation have entered a new stage. Methods of epidermal cell culture and transplantation: keratinocytes were cultivated on a feeder layer of mice fibroblast and obtained cultured epidermis sheet, and then transplanted into the wound with a complex composed of extracellular matrix that can be degraded and absorbed by the human body, so as to finally achieve the purpose of repairing the wound and improving the appearance.

Autologous cultured epidermal transplantation was first applied to burn patients in the early 1980s, and then applied to pigment deficiency diseases, such as congenital nevus and vitiligo. The utility model has the advantages that a larger area of leukoplakia can be treated by taking a smaller skin piece, and there is no scar formation. There is no rejection problem in autologous cultured epidermal transplantation. Co culture of keratinocytes and melanocytes to form epidermal slices has better curative effect in clinic. The combination of in vitro melanocyte culture technology and autologous epidermal culture transplantation technology establishes an autologous artificial cultured epidermis containing melanocytes, which not only solves the problem of cell attachment during melanocyte suspension injection, but also solves the problem of uneven pigment recovery at the skin lesions due to the small number and slow proliferation of melanocytes in the autologous epidermis, and meets the needs of patients.

**3.3 The significance and value of summarizing the effectiveness of autologous cultured epithelium grafting in the treatment of vitiligo**

Our department began to carry out the clinical technology for the treatment of vitiligo in 2011. In 2013, it was approved by Shanghai Health and Family Planning Commission for trial operation. One year later, it was approved by Shanghai Medical class III new technology in 2015 and officially put into clinical application. Since the development of this technology, nearly 1000 cases of clinical treatment have been completed, good clinical results have been achieved, and it has been recognized by peers at home and abroad. With the increase of the number of operations and follow-up patients, a large number of clinical data have been accumulated, which need to be analyzed retrospectively in order to better guide the clinical work. In the early stage, we have published two articles: preliminary clinical study on transplantation of tissue-engineered epidermal membranes with melanocytes in the treatment of vitiligo (Chinese Journal of Dermatology, 2015) and clinical application of cultured structured epithelial sheets, grow under feeder or feeder free conditions for stable vitiligo (dermatologic surgery, 2019), However, the data summarized by a large sample remains to be published. Since our department has become the unit with the largest number of treatment in this field and the longest follow-up patients have been more than 5 years, it is necessary to conduct retrospective statistical analysis on the completed patient data, summarize and analyze the safety and effectiveness of tissue engineering transplantation in the treatment of vitiligo, and analyze gender, age, clinical analysis, skin lesion location The relationship between the content of cultured epidermal melanocytes and surgical effect can fill the gap in this field at home and even abroad, and provide valuable clinical reference for peers.

**4 Type of study design:**

This clinical trial is a single center retrospective cohort study.

**5 Inclusion criteria and exclusion criteria of subjects**

**5.1 Selection criteria of subjects**

Patients with vitiligo, aged 14-85 years, male or female, who completed autologous cultured epithelium grafting and were followed up for at least 6 months;

**5.2 Exclusion criteria**

Patients with incomplete preoperative and postoperative data

**6 Study process and method**

**6.1 Clinical data collection**

Participants: 800 patients with vitiligo treated by autologous cultured epithelium grafting in the dermatology department of Huashan Hospital affiliated to Fudan University.

Collection time: autologous cultured epithelium grafting was completed from November 1, 2015 to June 30, 2019, and the last follow-up time was December 31, 2019.

Clinical data: according to the questionnaire, fill in item by item. The basic data and personal history of the patient are required to be complete, preoperative treatment methods, curative effects, postoperative adverse reactions and complications.

**Postoperative follow-up questionnaire for patients with autologous cultured epithelium grafting**

**Name□□□□ Gender male□ female□ Age □□ Education□□**

**Enrollment number □□□□ Inpatient number □□□□□□□□□□**

**Skin removal date Day Month Year**

**Operation date Day Month Year**

**Course of disease** ------------- **Stable period**-------------

**Diagnosis**-------------

**Classification** Focal vitiligo □ Segmental vitiligo □ Generalized vitiligo □

Vitiligo Universalis □ Mixed vitiligo □

**Complications** alopecia areata□ hyperthyroidism□ connective tissue disease □

others□

**Initial position** --------------------- **Inducement** -------------------------

**Previous treatment** -----------------------------

**Have you received surgical treatment?** No□ Yes□  **which method-**-----------------------

**Surgical site**-------------

**Affected area Cultured epidermal (**MC:KC) -------------

**The rate of repigmentation** -------------

**Postoperative adverse reactions and complications**

**Investigator** **Date**

**6.2 Clinical efficacy evaluation**

Sort out the postoperative follow-up photos of the patients, and the two dermatologists independently evaluated them. If they disagree, the third doctor is asked to evaluate, record the repigmentation rates and record the adverse reactions according to the follow-up history.

**7 Criteria for discontinuing clinical trials and provisions for ending clinical trials**

**7.1 Criteria for discontinuation of clinical trials**

Subjects asked to withdraw informed consent. If the investigator judges that it is unfavorable to the subject, the clinical trial can be suspended.

**7.2 Provisions on ending clinical trials**

The clinical trial will be terminated in the following cases: protocol design error; the study was found worthless during the study; the study sponsor stopped the study for reasonable reasons.

**8 Efficacy evaluation criteria**

**8.1** **Effective rates of vitiligo at operation site**

At the end of 6 months after the operation, the patients were evaluated the repigmentation rates according to the follow-up photos of the patients, and the repigmentation rates was independently evaluated by two dermatologists. Finally, the total effective rate was counted. The evaluation criteria were based on the clinical classification and curative effect criteria of vitiligo of pigmentation group of Dermatology and venereal diseases Professional Committee of Chinese society of integrated traditional and Western Medicine (revised in 2003):

Exellent improvement is all leukoplakia subsided and returned to normal skin color; Good improvement is that the leukoplakia partially subsides or shrinks, and the area of restoring normal skin color accounts for ≥ 50% of the lesion area; Fair improvement is that the leukoplakia partially subsides or shrinks; Poor improvement is that leukoplakia has no pigment regeneration or range expansion.

**8.2 Statistical relationship between gender and efficiency**

After input the patient's information, evaluate the relationship between patient gender and treatment efficiency, and search for the potential impact of gender on disease and treatment effect.

**8.3 Statistical Relationship between age and effective rate**

The influence of patients' age on the treatment effect is analyzed. The patients are divided into less than 14-year-old group and more than 14-year-old group. The effective rates of the two groups are counted respectively, and whether there is significant difference between the two groups is analyzed.

**8.4 Effective rate of different lesions**

Input the patient information and make statistics according to the patient's operation site. The operation site is divided into scalp, face and neck, trunk, upper limbs, lower limbs, back of hand, fingers and back of foot. Count the effective rate of each site in follow-up after operation, and analyze whether there are differences in the effective rate of surgical treatment between different skin lesions.

| Location | Number | Effective rate |
| --- | --- | --- |
| Scalp | 0 |  |
| Face | 1 |  |
| Neck | 2 |  |
| Trunk | 3 |  |
| upper limbs | 4 |  |
| lower limbs | 5 |  |
| back of hand | 6 |  |
| Finger | 7 |  |
| back of foot | 8 |  |

**8.5 Relationship between course of disease, stable period time and effective rate**

The influence of the course of disease and stable time on the effect of operation is evaluated to provide the basis for the best operation time in clinic.

**8.6 Patient's repigmentation type**

There are diffuse uniform repigmentation, perifollicular repigmentation and reticular repigmentation. The most common types of repigmentation are analyzed.

**8.7 Effect of melanocyte content in cultured tissue engineering skin on clinical efficiency**

Record the proportion of melanocytes and keratinocytes in each patient's epidermal sheet cultured in laboratory, and evaluate the relationship between this proportion and clinical efficiency, so as to find out the impact of laboratory cultured skin on clinical treatment.

**9. Statistical analysis methods**

In this analysis, all patients who completed autologous cultured epithelium grafting from November 1, 2015 to June 30, 2019 are included. All patients were followed up for 6 months. 800 patients were included in the analysis, and all effective cases in line with the scheme are statistically analyzed. Spss17 statistical software is used for statistical analysis. Descriptive analysis is used for counting data; Chi-square test or nonparametric test are used to analysis the changes between different groups before and after treatment.

**10. Provisions on data management and data traceability**

**10.1 Construction of research record form**

The data manager constructs the case report form according to the research protocol and the original clinical data.

**10.2 Data entry and modification**

The data entry and modification are entered by the researcher. The data source shall be consistent with the original data record sheet and laboratory checklist. Confirm that the original data is correctly and completely entered into the database: check the missing data, find and delete the repeatedly entered data, and check the uniqueness of some specific values (such as subject ID). The data administrator is responsible for the management and review of the entered data. When there are questions about the data, the data administrator raises questions to the researcher. The researcher shall reply in time. If not, the data administrator can question again.

**10.3 Study participants (subjects) information confidentiality plan**

The information of all study participants (subjects) must be strictly confidential, and the personal data participating in the study and in the study belong to the scope of confidentiality. Researchers must ensure that the privacy of clinical trial subjects is maintained. In all documents, the identity of the clinical trial subject can only be determined by the trial patient number and initials, the full name of the subject cannot be indicated. The investigator must properly keep the names and addresses of relevant clinical trial subjects and the corresponding enrollment forms. These enrollment forms shall be kept strictly confidential by the investigator and kept in a locked filing cabinet for researchers' reference only. When necessary, members of government administrative departments or ethics committees can access the personal data of researchers (subjects) in the research unit according to regulations. This result will not disclose any personal information of the study participants (subjects) when the research results are published.

**10.4 Research data confidentiality plan**

Research data belong to the scope of confidentiality, including investigator's manual, protocol, case report form, subject's personal data, informed consent, and any research data, records or other information generated in this clinical trial, collectively referred to as confidential information. The confidential information shall remain the confidential and proprietary property of the sponsor. The research data shall not be transferred to other units without the permission of the hospital. It is not allowed to transfer the research data involving human genetic information to foreign units or domestic units with foreign capital without the approval of the national human genetics office, except for the publication of research results that meet the requirements of laws and regulations under normal circumstances.

**11. Quality control and quality assurance of clinical trials**

The main researchers shall organize relevant training to ensure that the research is conducted in a standardized manner, and the filling of case report form or research record form and other reports follow the GCP principles and study protocol. The clinical trial quality control and quality assurance system ensures that the trial complies with the research protocol and management regulations, ensures the rights and interests of subjects in the clinical trial, ensures that the informed consent of all subjects is obtained at the beginning of the study, and ensures that the test records and reported data are accurate, complete and reliable. Clinical trial quality control is mainly through the formulation of clinical trial standard operating procedures. Any violation or deviation from the study protocol should be reported to the Ethics Committee immediately. When necessary, researchers should adopt standard operating procedures to ensure the quality control of clinical trials and the implementation of quality assurance system.

**12. Ethics related to the test**

**12.1 Ethics committee**

The clinical trial must comply with the declaration of Helsinki. Before the trial, the protocol can be implemented only after being approved by the hospital ethics committee. If there are problems in the implementation of this protocol, it is necessary to revise this protocol. After the main researchers organize a research group to discuss, the protocol shall be revised and reported to the ethics committee in writing form for approval and implementation.

**12.2 Informed consent**

This trial is a retrospective clinical study, involving three cases of informed consent. Subjects have the right to know more detailed information about this trial.

When the subjects come to the hospital for follow-up, they can sign the informed consent on site. If the subject cannot come to the hospital, the witness shall be present and the subject shall be informed of the test content by telephone. The basic information and clinical data of the subject shall be used to obtain the subject's informed consent. If the subject cannot come to the hospital for follow-up or contact, apply for exemption from informed consent and promise to keep the patient's information strictly confidential, which does not involve commercial interests.

The informed consent form (together with the trial protocol) must be reviewed and approved by the ethics committee. If necessary, the investigator is responsible for orally explaining the contents of the informed consent form to the subjects in a manner and wording understandable to the subjects. The subjects and their representatives should have enough time to read the informed consent form before formally signing it.

Informed consent must be signed and dated by the subject. Each signed informed consent form shall be properly kept and filed by the researcher.

**12.3 Confidentiality of subjects**

The researcher is responsible for maintaining the anonymity of the subjects. In the case report form or other documents, only capital letters, numbers and / or codes can be used to identify the subject, not the subject's name. The investigator must keep a subject inclusion form recording the subject code, name and home address. The researcher must keep strictly confidential the documents that can show the identity of the subjects.

**13. Subject recruitment method and process of obtaining informed consent**

The subjects were all from the dermatology department of Huashan Hospital who received autologous cultured epithelium grafting. There are three situations in the process of informed consent: when the subjects come to the hospital for follow-up, they can sign the informed consent form on site. If the subject cannot come to the hospital, the witness shall be present and the subject shall be informed of the test content by telephone. The basic information and clinical data of the subject shall be used to obtain the subject's informed consent. If the subject cannot come to the hospital for follow-up or contact, apply for exemption from informed consent and promise to keep the patient's information strictly confidential, which does not involve commercial interests.

The informed consent process of this trial complies with the rights and obligations of the subjects stipulated in the declaration of Helsinki, so that the subjects can fully understand and express their consent before conducting the clinical trial. At the same time, the subjects are informed that they have the right to withdraw from the test at any time. Before being selected, each subject must be given a written informed consent. The investigator is responsible for obtaining informed consent and signing informed consent before each subject enters the trial.

**14. Expected progress and completion date of clinical trial**

From May to June 2020: Complete clinical data statistics and patient evaluation

From July to August 2020: Writing articles

**15. References**

1. O’Connor NE, Mulliken JB, Banks-Schlegel S, et al. Grafting of burns with cultured epithelium prepared from autologous epidermal cells. Lancet 1981;1:75- 8.
2. Westerhof W, Lontz W, Vanscheidt W, Braathen L. Vitiligo: news in surgical treatment. J Eur Acad Dermatol Venereol 2001;15:510- 1.
3. Lerner AB, Halaban R, Klaus SN, Moellmann GE. Transplantation of human melanocytes. J Invest Dermatol 1987;89:219- 24.
4. Eisinger M, Marko O. Selective proliferation of normal human melanocytes in vitro in the presence of phorbol ester and cholera toxin. Proc Natl Acad Sci U S A 1982;79:2018- 22
5. Olsson MJ, Juhlin L. Repigmentation of vitiligo by transplantation of cultured autologous melanocytes. Acta Derm Venereol 1993;73: 49- 51.
6. Chen YF, Yang PY, Hung CM, Hu DN. Transplantation of autologous cultured melanocytes for treatment of large segmental vitiligo. J Am Acad Dermatol 2001;44:543-5.
7. Olsson MJ, Juhlin L. Long-term follow-up of leucoderma patients treated with transplants of autologous cultured melanocytes, ultrathin epidermal sheets and basal cell layer suspension. Br J Dermatol 2002;147:893- 904.
8. Guerra L, Primavera G, Raskovic D, et al. Erbium:YAG laser and cultured epidermis in the surgical therapy of stable vitiligo. Arch Dermatol 2003;139:1303- 10.
9. Issa CM, Rehder J, Taube MB. Melanocyte transplantation for the treatment of vitiligo: effects of different surgical techniques. Eur Dermatol 2003;13:34 - 9.
10. Andreassi L, Casini L, Trabucchi E, et al. Human keratinocytes cultured on membranes composed of benzyl ester of hyaluronic acid suitable for grafting. Wounds 1991;3:116- 26.
11. Hollander D, Stein M, Bernd A, et al. Autologous keratinocytes cultured on benzylester hyaluronic acid membranes in the treatment of chronic full-thickness ulcers. J Wound Care 1999;8:351- 5.
12. Elisa Pianigiani, Andrea Andreassi, Lucio Andreassi, Autografts and cultured epidermis in the treatment of vitiligo. Clinics in Dermatology (2005) 23, 424– 429
13. Pigmentation group of Dermatology and venereal diseases Professional Committee of Chinese society of integrated traditional Chinese and Western medicine, diagnosis and treatment standards of chloasma and vitiligo (2010 Edition), [J] Chinese Journal of dermatology 2010，43（6）：373
14. Andreassi L，Pianigiani E．Andreassi A。et a1．A new model of epidermal culture for the surgical treatment of vitiligo．Int J Dermatol 1998；37(8)：595-598
15. Lijian; Chen, Shujun; Uyama, Taro; Wu, Wenyu; Xu, Jinhua，Clinical Application of Cultured Stratified Epithelial Sheets Grown Under Feeder or Feeder-Free Conditions for Stable Vitiligo Dermatologic Surgery 2019 45(4)    : 497-505
16. 李剑 ，吴文育，陈淑君，傅雯雯，徐金华，Taro Uyama，徐昱，项蕾红，带黑素细胞的组织工程化表皮膜片移植治疗白癜风的初步临床研究 ，中华皮肤科杂志，2015.03.05，48（3）：162~165

**Protocol: KY2020-1137**

**1 Title:** **The mechanism of the repigmentation of tissue engineering autologous cultured epithelium grafting in the treatment of vitiligo**

**2 Purpose**

The primary aim of this investigation is to examine whether the reconstruction of melanocytes in the skin lesions post-grafting plays a pivotal role in the repigmentation process, shedding light on one of the critical mechanisms underlying skin graft-based repigmentation.

**3 Study background**:

Vitiligo, a common acquired disorder characterized by depigmentation, affects approximately 1-8% of the global population, with more than half of the cases emerging before the age of 20. It can manifest on various body regions, with a predilection for exposed areas such as the dorsal hands, wrists, forearms, face, neck, and genitalia. Vitiligo imposes a significant cosmetic burden on patients, and its treatment is often challenging. Even with long-term therapeutic interventions involving medications and phototherapy, up to 40% of patients experience relapse. Reduced or absent melanocyte populations and compromised melanocyte function at the lesion site are major contributing factors to the challenges of repigmentation in vitiligo patients. Therefore, current vitiligo treatments often incorporate surgical approaches, such as skin blister grafting.

In 2012, Huashan Hospital's Department of Dermatology pioneered the tissue-engineered autologous cultured epithelium grafting (ACEG) therapy for vitiligo. To date, this treatment has been applied to more than a thousand cases, with an overall effectiveness rate of approximately 90%. Impressively, over 58% of treated patients achieve near-complete repigmentation, demonstrating excellent clinical outcomes. Compared to other vitiligo treatment modalities, this approach offers advantages in terms of the treated area's size and repigmentation efficacy.

Although the prevailing view suggests that the reconstruction of melanocyte populations and functionality in the affected skin following surgical treatment is the primary mechanism responsible for therapeutic efficacy, no in-depth studies have been conducted by scholars to investigate this matter further. Based on relevant literature and clinical observations, we hypothesize that some other skin cells, present within the grafts may play a pivotal role in reconstructing melanocyte populations and their functionality at the lesion site.

Hence, our study aims to assess whether graft-based repigmentation therapy can effectively restore the quantity and functionality of melanocytes in the skin lesions by comparing their levels before and after treatment. Moreover, by analyzing the number of melanocytes in patients' donor skin, tissue-engineered grafts, and repigmented skin post-treatment, we seek to identify the optimal ratio of melanoblasts for reconstructing melanocyte functionality within the grafts. Additionally, we plan to employ single-cell transcriptome sequencing technology to compare the differences in cell composition between patients' normal donor skin and tissue-engineered grafts, as well as to analyze variations in gene expression at the cellular level in normal skin and grafts. Ultimately, this research aims to elucidate the mechanisms underlying skin repigmentation post-tissue-engineered graft transplantation for vitiligo, optimize the cellular composition of tissue-engineered skin, and provide a crucial theoretical foundation for enhancing post-transplantation effectiveness.

**4 Type of study design:**

This clinical trial is a single center prospective study.

**5 Inclusion criteria and exclusion criteria of subjects**

**5.1 Selection criteria of subjects**

(1) Patients aged 14-85 years, male or female, stable vitiligo (defined as no progression of existing lesions, no appearance of new lesions, and no Koebner phenomenon for at least 12 months) at more than one site;

(2) Patients with inadequate response to a variety of medical treatments for vitiligo;

(3) Patients with willingness and ability to undergo treatment with ACEG;

(4) Patients with informed consent.

**5.2 Exclusion criteria**

(1) Patients who decide not to receive transplantation of ACEG for the treatment of stable vitiligo;

(2) Patients with unwillingness to collect the sample for scientific research, private area lesions, extensive depigmented areas (defined as an area covering more than 1% of body surface area);

(3) Patients with serious systemic diseases, keloid diathesis, or infections.

**6 Study process and method**

**6.1 Subject Questionnaires:**

(1) Subjects completed questionnaires, providing essential information such as names, ages, disease diagnoses, and informed consent signatures. Those meeting the inclusion criteria were included, while those not meeting the exclusion criteria were considered. (Refer to the questionnaire for details)

(2) Assessment of hidden illnesses in healthy volunteers.

(3) Comprehensive inquiries were conducted among vitiligo patients regarding their staging, subtypes, comorbidities, treatment history, and treatment outcomes.

**Subject Questionnaires**

Name: ________ Gender: Male □ Female □ Age: ____

Diagnosis: ____________________________________

Comorbidities:

None □

Yes □ (Specify disease(s): Hypertension □, Diabetes □, Connective Tissue Disease □, Thyroid Disease □, Tumor □)

For Vitiligo Subjects (To be filled by vitiligo subjects):

Family History: _____________________________

Disease Duration: ___________________________

Classification:

Non-Segmental □ (Localized □, Disseminated □, Universal □, Acrofacial □)

Segmental □

Mixed □

Disease Stage: Active □ Stable □

Previous Treatment(s): ______________________

Investigator：

Date：

**6.2 Sample collection**

For five patients with extensive stable-phase vitiligo requiring multiple surgeries specimens were obtained and reserved as follows:

a) Normal skin control: Used for preparing normal skin samples for graft production. Small skin samples (0.5cm x 0.5cm) were collected during routine skin removal procedures. One-quarter of the sample was used for immunofluorescence testing, and three-quarters were used for cell flow cytometry.

b) Tissue-engineered cultured epithelium: The remaining cultured tissue-engineered epidermal grafts typically used for melanocyte detection, immunofluorescence testing, and flow cytometry, measuring approximately 3cm2. These grafts do not affect skin quality assessment or treatment in patients.

**6.3. Immunofluorescence:**

Using the same parameters, cell immunofluorescence staining was performed on the samples from all groups. The related cells were recorded using the Tile function, with ten fields photographed and then averaged.

**6.4. Flow Cytometry:**

Employing the same parameters, cell flow cytometry was conducted on the aforementioned samples. Cell counts for related cells were measured in triplicate, with 10,000 cells analyzed each time. Data analysis was performed using FlowJo.

**6.5. Comparison of Cellular Composition in Grafts and Normal Skin through Single-Cell Sequencing:**

Normal skin and cultured tissue-engineered epidermal grafts were digested to prepare single-cell suspensions, followed by sequencing using the following methods:

a) Sequencing Strategy: Single-cell library sample preparation was performed using the 10X Genomics Chromium Controller instrument, according to the 10X Genomics Chromium Single Cell 3' Reagent Kits instructions. The Illumina Nova 6000 sequencing platform was used, with a PE150 library construction strategy, capturing approximately 5000-10000 cells per sample.

b) GEM and Barcode Preparation: The diluted cell suspension was mixed with Master Mix, and the mixture, as well as Gel Beads, was accurately added to their respective positions on the Next GEM Chip G flat panel. Partitioning Oil was then added. The Next GEM Chip G was placed in the 10X Chromium Controller for GEM preparation, followed by reverse transcription and cDNA amplification.

c) Establishment of Gene Expression Libraries: Ideal-sized cDNA amplicons were obtained using Enzymatic fragmentation and size selection. TruSeq Read 1 sequence was added during GEM incubation. The GEM received P5, P7, sequencing labels, and TruSeq Read 2 sequence during end repair, A-tailing, and adaptor ligation. The cell library with P5 and P7 primers was generated. After library construction, initial quantification was conducted using Qubit 2.0, and insert size was assessed with Agilent 2100. Effective library concentration was determined through Q-PCR to ensure library quality.

d) On-machine Sequencing: Prepared cDNA libraries were sequenced using the Illumina NovaSeq 6000 sequencer. Sequencing depth was 300M Reads, with PE150 sequencing type, and sample concentration during sequencing was 200pM.

e) Quality Assessment of Sequencing Results: CASAVA base recognition analysis converted the Illumina NovaSeq 6000 raw image data files into raw sequencing sequences. Sequencing errors were determined based on Phred scores (Formula 1: Qphred = -10log10(e)). Basic statistics on data quality were performed using FastQC.

f) Raw Sequencing Filtering: Trimmomatic software was used to remove low-quality reads and adapters, calculate sequencing error rates, and assess Q20, Q30, and GC content.

g) CleanData Data Quality Assessment: The same methods were used as for raw data assessment.

h) Data Statistics and Analysis: Data statistics and analysis were conducted using Cell Ranger software.

i) Reference Gene Alignment: The star plugin in Cell Ranger was used for reference gene alignment, with the reference genome being the standard Han Chinese whole genome. Reads mapped to the reference genome were corrected using GTF annotation files and differentiated into exonic regions, intronic regions, and intergenic regions.

j) Enumeration of Captured Cell Numbers: Cell Ranger identified barcodes to separate reads from each cell and then filtered and processed the samples to count the number of cells, the number of reads per cell, and the number of genes detected in each sample.

k) Clustering and Differential Expression Analysis:

1. Dimensionality Reduction Analysis: Principal component analysis was used to reduce multidimensional data and extract the primary structure of the data. Clustering Analysis: Two methods provided by Cell Ranger were used to cluster cells with similar expression.

2. Graph-based Clustering Algorithm: PCA-reduced data was used to construct a sparse matrix of k-nearest neighbors among cells, grouping each cell with its nearest k cells based on Euclidean distance. The Louvain algorithm was then applied for module optimization. K-means Algorithm: A suitable number of cluster centroids (k, representing the assumed number of cell subgroups) were randomly chosen in PCA-reduced space. Each cell was assigned to its corresponding cluster, and the cluster centroid was recalculated for each cluster until convergence. Differential Analysis: Cell Ranger used the sSeq method to find differentially expressed genes between various cell subgroups. Marker Gene Analysis: High-expressing differentially expressed genes within each cluster, as analyzed by Cell Ranger, were presented in three forms: violin plots, t-SNE plots, and heatmaps.

l) Functional Enrichment Analysis of Differential Genes: Enrichment analysis of highly expressed differentially expressed genes within each cluster was performed to identify significant biological functions or pathways. The clusterProfiler software was used for enrichment analysis of highly expressed differentially expressed genes in each cluster.

m) GO Functional Enrichment Analysis: GO (Gene Ontology, http://geneontology.org/) is a comprehensive database describing gene functions. GOseq software was used to perform enrichment with significance set at padj (p-values after multiple corrections) less than 0.05.

n) KEGG Pathway Enrichment Analysis: KEGG (Kyoto Encyclopedia of Genes and Genomes, https://www.kegg.jp/) is a comprehensive database that integrates genome, chemical, and system function information. KOBAS (2.0) software was used for enrichment analysis with significance set at padj less than 0.05.

o) Pseudo-Temporal Trajectory Analysis: Based on clustering results, the DDRTree method was used for dimensionality reduction and minimum spanning tree construction. Single-cell data was then fitted to the best cell development or differentiation pseudo-temporal trajectory curve through high-dimensional and low-dimensional space searching.

**7 Criteria for discontinuing clinical trials and provisions for ending clinical trials**

**7.1 Criteria for discontinuation of clinical trials**

Subjects asked to withdraw informed consent. If the investigator judges that it is unfavorable to the subject, the clinical trial can be suspended.

**7.2 Provisions on ending clinical trials**

The clinical trial will be terminated in the following cases: protocol design error; the study was found worthless during the study; the study sponsor stopped the study for reasonable reasons.

**8 Adverse events records**

**8.1 Adverse Events**

Adverse events are defined as any unfavorable medical occurrences experienced by patients or clinical trial subjects following the administration of a medical product. These events need not necessarily have a causal relationship with the treatment. They encompass any untoward signs, symptoms, or diseases that occur in temporal association with the use of the medical product, regardless of whether an association with the medical product is suspected.

The relationship of all adverse events to the surgical transplantation study is evaluated on a five-tier scale: positive relationship, very likely related, possibly related, possibly unrelated, and definitely unrelated. The first three categories are considered related to this study.

**8.2 Serious Adverse Events or Reactions**

Serious adverse events or reactions refer to any unfavorable medical events that occur at any dose, including those that lead to death, pose a threat to life, necessitate hospitalization or prolong the duration of hospitalization, result in significant or persistent disability or loss of work capacity, cause congenital anomalies, or represent other clinically significant medical events or reactions.

**8.3 Measures for Adverse Reactions and Adverse Events**

1. Pain or prolonged bleeding after blood withdrawal will be addressed by applying pressure for hemostasis and monitoring until symptoms resolve.

2. Adverse reactions related to pathological biopsies and surgical site issues will be managed following standard surgical procedures. In the case of infections, appropriate anti-infective treatments will be administered. For prominent scarring, treatment options may include topical steroid ointments, silicone gel, and heparinoid ointments, among others.

**9. Statistical analysis**

This experiment is prospective and exploratory. Based on relevant vitiligo literature and differences in cytokine expression, the study is divided into three groups: active vitiligo, stable vitiligo, and healthy volunteers, with 30 participants in each group. Ten tissue specimens are included in each group. Statistical analysis is performed using SPSS version 17. Categorical data is assessed using chi-square tests, while quantitative data is presented as means ± standard deviations. Cytokine expression is represented as means. Group comparisons are conducted using between-group t-tests, with the significance level set at α = 0.05. A p-value (P) greater than 0.05 indicates no statistical difference, while a P-value less than 0.05 indicates a statistically significant difference.

**10. Provisions on data management and data traceability**

**10.1 Construction of research record form**

The data manager constructs the case report form according to the research protocol and the original clinical data.

**10.2 Data entry and modification**

The data entry and modification are entered by the researcher. The data source shall be consistent with the original data record sheet and laboratory checklist. Confirm that the original data is correctly and completely entered into the database: check the missing data, find and delete the repeatedly entered data, and check the uniqueness of some specific values (such as subject ID). The data administrator is responsible for the management and review of the entered data. When there are questions about the data, the data administrator raises questions to the researcher. The researcher shall reply in time. If not, the data administrator can question again.

**10.3 Study participants (subjects) information confidentiality plan**

The information of all study participants (subjects) must be strictly confidential, and the personal data participating in the study and in the study belong to the scope of confidentiality. Researchers must ensure that the privacy of clinical trial subjects is maintained. In all documents, the identity of the clinical trial subject can only be determined by the trial patient number and initials, the full name of the subject cannot be indicated. The investigator must properly keep the names and addresses of relevant clinical trial subjects and the corresponding enrollment forms. These enrollment forms shall be kept strictly confidential by the investigator and kept in a locked filing cabinet for researchers' reference only. When necessary, members of government administrative departments or ethics committees can access the personal data of researchers (subjects) in the research unit according to regulations. This result will not disclose any personal information of the study participants (subjects) when the research results are published.

**10.4 Research data confidentiality plan**

Research data belong to the scope of confidentiality, including investigator's manual, protocol, case report form, subject's personal data, informed consent, and any research data, records or other information generated in this clinical trial, collectively referred to as confidential information. The confidential information shall remain the confidential and proprietary property of the sponsor. The research data shall not be transferred to other units without the permission of the hospital. It is not allowed to transfer the research data involving human genetic information to foreign units or domestic units with foreign capital without the approval of the national human genetics office, except for the publication of research results that meet the requirements of laws and regulations under normal circumstances.

**11. Quality control and quality assurance of clinical trials**

The main researchers shall organize relevant training to ensure that the research is conducted in a standardized manner, and the filling of case report form or research record form and other reports follow the GCP principles and study protocol. The clinical trial quality control and quality assurance system ensures that the trial complies with the research protocol and management regulations, ensures the rights and interests of subjects in the clinical trial, ensures that the informed consent of all subjects is obtained at the beginning of the study, and ensures that the test records and reported data are accurate, complete and reliable. Clinical trial quality control is mainly through the formulation of clinical trial standard operating procedures. Any violation or deviation from the study protocol should be reported to the Ethics Committee immediately. When necessary, researchers should adopt standard operating procedures to ensure the quality control of clinical trials and the implementation of quality assurance system.

**12. Ethics related to the test**

**12.1 Ethics committee**

The clinical trial must comply with the declaration of Helsinki. Before the trial, the protocol can be implemented only after being approved by the hospital ethics committee. If there are problems in the implementation of this protocol, it is necessary to revise this protocol. After the main researchers organize a research group to discuss, the protocol shall be revised and reported to the ethics committee in writing form for approval and implementation.

**12.2 Informed consent**

This trial is a retrospective clinical study, involving three cases of informed consent. Subjects have the right to know more detailed information about this trial.

When the subjects come to the hospital for follow-up, they can sign the informed consent on site. If the subject cannot come to the hospital, the witness shall be present and the subject shall be informed of the test content by telephone. The basic information and clinical data of the subject shall be used to obtain the subject's informed consent. If the subject cannot come to the hospital for follow-up or contact, apply for exemption from informed consent and promise to keep the patient's information strictly confidential, which does not involve commercial interests.

The informed consent form (together with the trial protocol) must be reviewed and approved by the ethics committee. If necessary, the investigator is responsible for orally explaining the contents of the informed consent form to the subjects in a manner and wording understandable to the subjects. The subjects and their representatives should have enough time to read the informed consent form before formally signing it.

Informed consent must be signed and dated by the subject. Each signed informed consent form shall be properly kept and filed by the researcher.

**12.3 Confidentiality of subjects**

The researcher is responsible for maintaining the anonymity of the subjects. In the case report form or other documents, only capital letters, numbers and / or codes can be used to identify the subject, not the subject's name. The investigator must keep a subject inclusion form recording the subject code, name and home address. The researcher must keep strictly confidential the documents that can show the identity of the subjects.

**13. Subject recruitment method and process of obtaining informed consent**

The subjects were all from the dermatology department of Huashan Hospital who received autologous cultured epithelium grafting. There are three situations in the process of informed consent: when the subjects come to the hospital for follow-up, they can sign the informed consent form on site. If the subject cannot come to the hospital, the witness shall be present and the subject shall be informed of the test content by telephone. The basic information and clinical data of the subject shall be used to obtain the subject's informed consent. If the subject cannot come to the hospital for follow-up or contact, apply for exemption from informed consent and promise to keep the patient's information strictly confidential, which does not involve commercial interests.

The informed consent process of this trial complies with the rights and obligations of the subjects stipulated in the declaration of Helsinki, so that the subjects can fully understand and express their consent before conducting the clinical trial. At the same time, the subjects are informed that they have the right to withdraw from the test at any time. Before being selected, each subject must be given a written informed consent. The investigator is responsible for obtaining informed consent and signing informed consent before each subject enters the trial.

**14. Expected progress and completion date of clinical trial**

From October to December 2020: Complete clinical data statistics and patients’ sample collection

From January to March 2021: Laboratory experiments

From April to June 2021: Data analysis and article preparation

**15. References**

1. Grosshans, E., D. Sengel, and E. Heid, *[White lentiginosis].* Ann Dermatol Venereol, 1994. **121**(1): p. 7-10.

2. Manga, P., N. Elbuluk, and S.J. Orlow, *Recent advances in understanding vitiligo.* F1000Res, 2016. **5**.

3. Pillaiyar, T., M. Manickam, and S.H. Jung, *Inhibitors of melanogenesis: a patent review (2009 - 2014).* Expert Opin Ther Pat, 2015. **25**(7): p. 775-88.

4. Hombach, S. and M. Kretz, *The non-coding skin: exploring the roles of long non-coding RNAs in epidermal homeostasis and disease.* Bioessays, 2013. **35**(12): p. 1093-100.

5. Kretz, M., et al., *Suppression of progenitor differentiation requires the long noncoding RNA ANCR.* Genes Dev, 2012. **26**(4): p. 338-43.

6. Zhu, L. and P.C. Xu, *Downregulated LncRNA-ANCR promotes osteoblast differentiation by targeting EZH2 and regulating Runx2 expression.* Biochem Biophys Res Commun, 2013. **432**(4): p. 612-7.

7. Kretz, M., et al., *Control of somatic tissue differentiation by the long non-coding RNA TINCR.* Nature, 2013. **493**(7431): p. 231-5.

8. Zeng, Q., et al., *Analysis of lncRNAs expression in UVB-induced stress responses of melanocytes.* J Dermatol Sci, 2016. **81**(1): p. 53-60.

9. Cai, B., et al., *Long noncoding RNA H19 mediates melatonin inhibition of premature senescence of c-kit(+) cardiac progenitor cells by promoting miR-675.* J Pineal Res, 2016. **61**(1): p. 82-95.

10. Ortonne, Jean-Paul & Mosher, David & Fitzpatrick, Thomas. (1983). Vitiligo and Other Hypomelanoses of Hair and Skin. 10.1007/978-1-4615-9272-3.

**Protocol: KY2020-698**


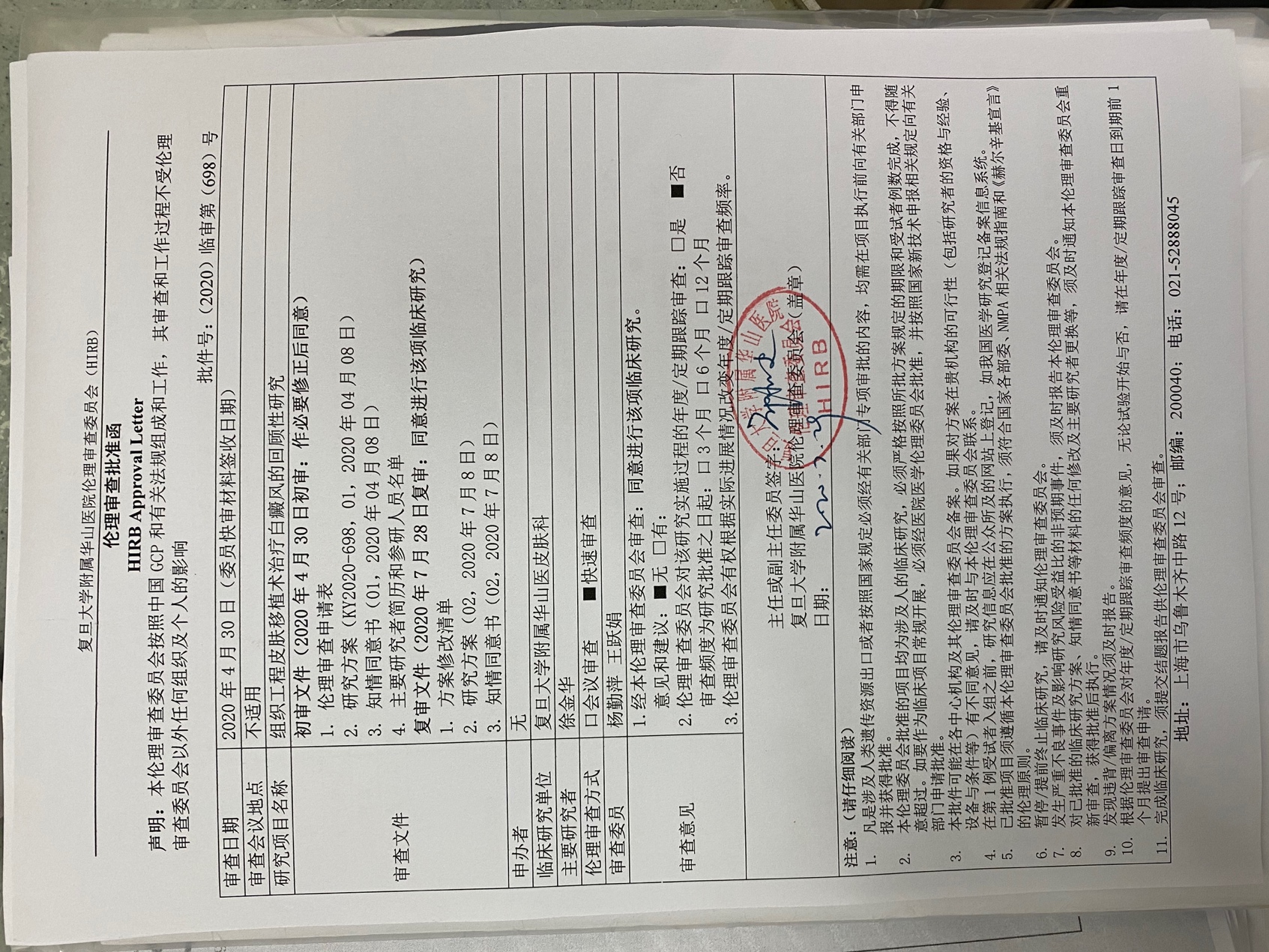


**Protocol: KY2020-1137**


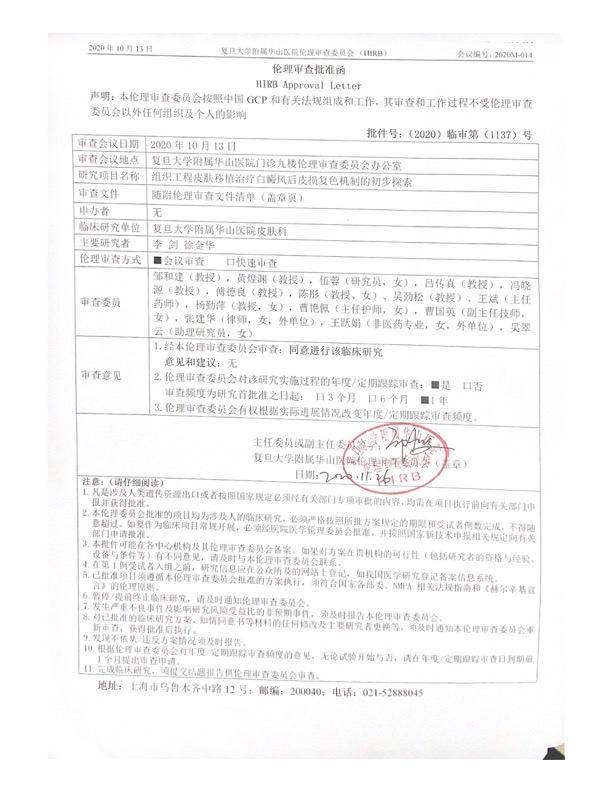


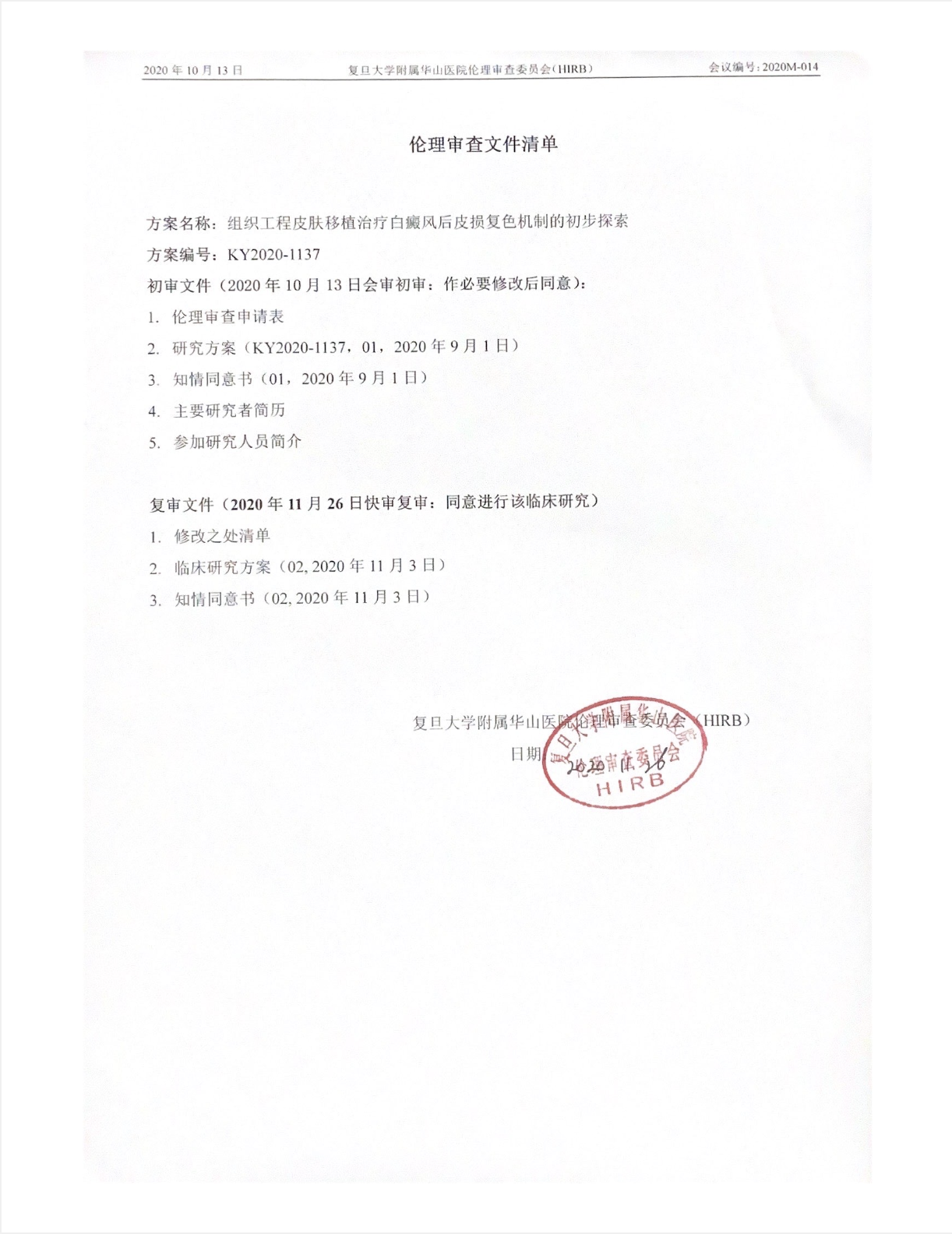


**ICF Template**

**Name of protocol：retrospective study of autologous cultured epithelium grafting in the treatment of vitiligo**

You will be invited to participate in a retrospective study of tissue-engineered skin transplantation in the treatment of vitiligo. These instructions provide you with some information to help you decide whether to participate in this clinical study. Please read it carefully. If you have any questions, please ask the researcher in charge of the study.

Your participation in this study is voluntary. This study has been reviewed by the ethics review committee of this research institution.

I. Research background and purpose

Vitiligo is a common disease in dermatology clinic. Due to its complex etiology, difficult treatment and long course of treatment, it has brought great psychological and economic burden to patients. Autologous cultured epithelium grafting for the treatment of vitiligo is a new technology explored by the dermatology department of Huashan Hospital since 2012. This clinical technology obtained three new medical technologies in Shanghai in 2013 and was officially launched in 2015. The technology aims to treat vitiligo patients with large area and difficult to repigment, and fill the gap in this field in China. Since its development, our department has completed nearly 1000 cases of clinical treatment, achieved good clinical results, and has been recognized by peers at home and abroad. With the increase of the number of operations and follow-up patients, our department has become the unit with the largest number of treatment in this field, and the longest follow-up patients have been more than 5 years. Therefore, it is necessary to conduct retrospective statistical analysis on the completed patient data, summarize and analyze the safety and effectiveness of autologous cultured epithelium grafting in the treatment of vitiligo, and analyze gender, age, clinical analysis and skin lesion location. The results will provide valuable clinical reference for peers, so that the technology can better serve the clinic.

II. Test process

We will collect and sort out the clinical data of subjects who completed autologous cultured epithelium grafting from November 1, 2015 to June 30, 2022. Collect personal data such as age and gender, medical history, surgical records and clinical follow-up photos, evaluate the efficiency of patients, analyze the efficiency and safety of the technology, clinical pictures and sort out the documents for publication (might be made of the published case report, including as applicable publication in print or online and whether freely available or by subscription, in audio or video recordings and presentations, webinars, etc). To provide guidance for further clinical application.

If you agree to participate in this study, we will number you and establish medical records.

Risks and discomfort: the analysis may use your clinical photos, but do not expose facial features and physical features.

Potential benefit: it has no direct benefit to you and is helpful to the diagnosis and treatment of diseases.

Compensation: you will not be paid for participating in this experiment.

Alternative therapy other than this trial: continue outpatient follow-up treatment

Privacy: if you decide to participate in this study, your personal data in the study are confidential. Your blood / urine sample will be identified by the study number instead of your name. Information that identifies you will not be disclosed to members outside the research team unless you obtain your permission. All research members and research sponsors are required to keep your identity confidential. Your files will be kept in a locked filing cabinet for researchers only. In order to ensure that the research is carried out in accordance with the regulations, if necessary, members of the government management department or the ethics review committee can access your personal data in the research unit in accordance with the regulations. Your personal information will not be disclosed when the results of this study are published.

If you are injured due to your participation in this study: in case of damage related to this clinical study, you can receive free treatment and / or corresponding compensation.

You can choose not to participate in this study, or notify the researcher to withdraw from the study at any time. Your data will not be included in the research results, and any medical treatment and rights will not be affected.

If you need other treatment, or you do not comply with the study plan, or there is a study related injury, or for any other reason, the study physician may terminate your continued participation in the study.

You can keep abreast of the information and research progress related to this study at any time. If there is any new safety information related to this study, we will inform you in time. If you have questions related to this study, or you have any discomfort and injury during the study, or have questions about the rights and interests of participants in this study, you can contact Li Jian at 13817637673

If you have any questions or demands on the rights and health of participating in this study, you can contact the ethics committee of our institution at 52888045; Contact: Wu Cuiyun.

**Informed consent signature page**

I have read this informed consent form.

I have had the opportunity to ask questions and all the questions have been answered.

I understand that participation in this study is voluntary.

I can choose not to participate in this study, or quit after notifying the researcher at any time without discrimination or retaliation, and my medical treatment and rights will not be affected.

If I need other treatment, or I do not comply with the study plan, or there is a study related injury, or for any other reason, the study physician may terminate my continued participation in the study.

I will receive a signed copy of the informed consent form.

Subject name:________________________

Subject signature： ___________________

Date：_____________________________

I have accurately informed the subject of this document. He / she has accurately read this informed consent form and has the opportunity to ask questions.

Name of investigator:________________________

Investigator signature:_______________________

Date:____________________________________

(Note: if the subject is illiterate, the signature of the witness is required; if the subject is incapacitated, the signature of the agent is required)

**Informed consent signature page**

I have been orally informed of this informed consent form and cannot come to the hospital for on-site signature.

I have had the opportunity to ask questions and all the questions have been answered.

I understand that participation in this study is voluntary.

I can choose not to participate in this study, or quit after notifying the researcher at any time without discrimination or retaliation, and my medical treatment and rights will not be affected.

If I need other treatment, or I do not comply with the study plan, or there is a study related injury, or for any other reason, the study physician may terminate my continued participation in the study.

Subject name:________________________

Subject signature： ___________________

Date：_____________________________

I have accurately informed the subject of this document. He / she has accurately read this informed consent form and has the opportunity to ask questions.

Name of investigator:________________________

Investigator signature:________________________

Date：____________________________________

(Note: if the subject is illiterate, the signature of the witness is required. If the subject has no behavior

Name of witness:________________________

Signature of witness:_____________________

Date：_____________________________

**Informed consent signature page**

For the subject of "retrospective study on the treatment of vitiligo with tissue-engineered skin transplantation", I plan to retrospectively collect the diagnosis and treatment data of vitiligo subjects who completed tissue-engineered skin transplantation between November 1, 2015 and June 30, 2019, and will try my best to contact the subjects for informed consent, but apply for exemption from informed consent for subjects who can not be found, We hereby promise to keep the patient information strictly confidential and do not involve commercial interests. We hereby request the approval of the ethics committee.

Name of investigator: ________________________

Investigator signature: ________________________

Date：_____________________________________

**Sample Informed Consent Form Template**

**Protocol Name: The mechanism of the repigmentation of tissue engineering autologous cultured epithelium grafting in the treatment of vitiligo**

Purpose of the research: Vitiligo is a common skin depigmentation disorder that damages the appearance. In response to the difficulty of treatment of some patients and the difficulty of repigmentation, we have carried out tissue engineering autologous cultured epithelium grafting (ACEG) to treat vitiligo since the end of 2011. So far, nearly 1,000 operations have been completed and have achieved good clinical results. The purpose of the study is to explore the mechanism of ACEG and better promote this new technology.

Research process: We will select 5 patients who plans to receive ACEG for vitiligo, and collect small pieces (about 1 cm x 1cm) of skin sample from the donor sites of patients, typically the groin, on the day of donor collection surgery. In this study, there are two groups of samples will be used:

A) Normal skin sample: normal skin samples are used to prepare cultured epithelium. On the day of donor collection surgery, cut more 1cm x1cm of skin samples when obtaining the normal skin. It is used for immunofluorescence, flow cytometry, and single-cell RNA sequencing.

B) Autologous cultured epithelium: The tissue engineering autologous cultured epithelium is cultivated from normal skin samples. Autologous cultured epithelium is used for melanocyte detection and grafting. The remaining epithelium is used for immunofluorescence, flow cytometry, and single-cell RNA sequencing. It does not affect the epithelium quality detection and subsequent treatment.

The subjects only need to come to the hospital based on regular treatment and do not need extra time to come to the hospital. The obtained skin samples are tested for molecular biology experiments, and the results are only used for scientific research. If you agree to participate in this study, we will number you and establish a medical record file.

Risk and discomfort: For you, all information will be confidential. Your operation will be operated by professionals such as surgeons, and we only collect some samples for scientific research.

Benefit: Studying your samples will help you understand the repigmentation mechanism of tissue engineering autologous cultured epithelium transplantation, which is not directly beneficial to you personally.

Cost: You don't need to bear any additional costs.

Compensation: You will receive a transportation subsidy of 200 RMB.

As a study subject, you have the following duties: to provide the real situation of your medical history and current physical condition; tell the research doctor about any discomfort you have experienced during this study; and tell the research doctor whether you have participated in other studies recently, or are currently participating in other studies.

Privacy: If you decide to participate in this study, your personal data in the study and in the research are confidential. Your tissue samples will be identified by the research number instead of your name. Information that can identify you will not be disclosed to members outside the research team unless your permission is obtained. Your files will be stored in a locked filing cabinet for researchers only. In order to ensure that the research is carried out in accordance with the regulations, members of the government administration or ethics review committee can access your personal data at the research unit if necessary. When the results of this study are published, no personal information will be disclosed. In case of damage related to clinical research, you can get free treatment and/or corresponding compensation. You can choose not to participate in this study, or notify the researcher to withdraw from the study at any time. Your data will not be included in the research results, and any of your medical treatment and rights will not be affected. After the study, samples are destroyed. If you need other treatment, or if you do not comply with the research plan, or if you have suffered a damage related to the research or for any other reason, the researcher can terminate your continued participation in this study. You can keep abreast of the information and research progress related to this research. If new privacy information related to this research occurs, we will also inform you in time.

If you have any questions related to this study, or if you have any discomfort or damage during the research, or if you have any questions about the rights and interests of the participants in this study, you can contact Zeng Xuanhao at 13122566675.

If you have any questions or demands about the rights of participating in this study, you can contact the ethics committee of this institution at 52888045; contact person: Wu Cuiyun.

**Informed consent signature page**

I have read this informed consent form.

I have had the opportunity to ask questions and all the questions have been answered.

I understand that participation in this study is voluntary.

I can choose not to participate in this study, or quit after notifying the researcher at any time

without discrimination or retaliation, and my medical treatment and rights will not be affected.

If I need other treatment, or I do not comply with the study plan, or there is a study related

injury, or for any other reason, the study physician may terminate my continued participation in

the study.

I will receive a signed copy of the informed consent form.

Subject name: ______________________________

Subject signature: ___________________________

Date: _____________________________________

I have accurately informed the subject of this document. He / she has accurately read this

informed consent form and has the opportunity to ask questions.

Name of investigator: ________________________

Investigator signature: ________________________

Date: _____________________________________

(Note: if the subject is illiterate, the signature of the witness is required; if the subject is

incapacitated, the signature of the agent is required)
