# Supplementary material for: Single-cell sequencing reveals increased LAMB3-positive basal keratinocytes and ZNF90-positive fibroblasts in autologous cultured epithelium
Source: Commun Biol. 2024 Jan 10;7:79. doi: 10.1038/s42003-023-05747-5 (PMC10781733; doi:10.1038/s42003-023-05747-5)
Supplement: Supplementary file 8 — Supplementary Data 5 [file 42003_2023_5747_MOESM8_ESM.docx]

**Supplement file. S4 sc-RNA seq Methods**

**Methods**

**In the cell preparation section:** For the quality check and counting of single cell suspension, the cell survival rate is generally above 80%. The cells that have passed the test are washed and resuspended to prepare a suitable cell concentration of 700~1200 cells/ul for 10x Genomics Chromium^TM^. For each sample, 6000-10000 cells were aimed to captured. The system is operated on the machine.

**Steps in GEM Creation & Thermal cycling:** The single cell cDNA library was created by Chromium Next GEM Single Cell 3' Reagent Kits v3.1 and 10X Genomic Chromium followed the manufacture. GEMs (Gel Bead in Emulsion) were constructed for single cell separation according to the number of cells to be harvested. After GEMs were normally formed, GEMs were collected for reverse transcription in a PCR machine for labeling.

**Post Cycling Cleanup & cDNA Amplication:** The GEMs were oil-treated, and the amplified cDNA was purified by magnetic beads, and then subjected to cDNA amplification and quality inspection.

**Library Preparation & Quantification:** The 3ʹ Gene Expression Library was constructed with the quality-qualified cDNA. After fragmentation, adaptor ligation, sample index PCR, etc., the library is finally quantitatively examined.

**Sequencing:** The final library pool was sequenced on the Illumina Novaseq 6000 instrument using 150-base-pair paired-end reads. For each sample 300M/cells data were collected.

| **Data base** | **website** |
| --- | --- |
| **Genome** | <ftp://ftp.ensembl.org/pub/release-*/fasta/> |
| **GTF** | <ftp://ftp.ensembl.org/pub/release-*/gtf/> |
| **GeneOntology** | http://geneontology.org/ |
| **Reactome** | https://reactome.org/ |
| **GSEA** | https://www.gsea-msigdb.org/gsea/index.jsp |
| **CellPhoneDb** | [https://www.cellphonedb.org/](https://www.cellphonedb.org/%20) |
| **CellMarker** | [http://biocc.hrbmu.edu.cn/CellMarker/](http://biocc.hrbmu.edu.cn/CellMarker/  ) |

| Software | website |
| --- | --- |
| R 4.1.3 | https://cran.r-project.org/src/base/R-4/ |
| Python 3.6.13 | https://www.python.org/ |
| Cell Ranger | https://support.10xgenomics.com/single-cell-gene-expression/software/downloads/latest |
| Loupe Browser | https://support.10xgenomics.com/single-cell-gene-expression/software/downloads/latest#loupe |
| Seurat4.2.0 | https://satijalab.org/seurat/index.html |
| clustertree | https://github.com/peekxc/clustertree |
| Monocle3 | https://cole-trapnell-lab.github.io/monocle3/ |
| GSEA 1.60.0 | https://bioconductor.org/packages/release/bioc/html/GSEABase.html |
| GSVA 1.46.0 | https://bioconductor.org/packages/release/bioc/html/GSVA.html |
| Limma 3.54.0 | https://bioconductor.org/packages/release/bioc/html/limma.html |
| CellphoneDb v4 | https://github.com/ventolab/CellphoneDB |
| ggplot2 3.4.0 | https://ggplot2.tidyverse.org/ |
| Harmony 0.1.1  ClusterProfiler 4.6.2 | <https://github.com/immunogenomics/harmony>  <https://bioconductor.org/packages/release/bioc/html/clusterProfiler.html> |
