# Supplementary material for: Single-cell sequencing reveals increased LAMB3-positive basal keratinocytes and ZNF90-positive fibroblasts in autologous cultured epithelium
Source: Commun Biol. 2024 Jan 10;7:79. doi: 10.1038/s42003-023-05747-5 (PMC10781733; doi:10.1038/s42003-023-05747-5)
Supplement: Supplementary file 9 — Supplementary Data 6 [file 42003_2023_5747_MOESM9_ESM.pdf]

---
title: "Seurat标准流程"
author: "Skinsheet&ZQ"
date: "2022/4/19"
output: html_document

R Markdown

This is an R Markdown document. Markdown is a simple formatting syntax for authoring HTML, PDF, and MS Word documents. For more details on using R Markdown see <http://rmarkdown.rstudio.com>.

When you click the **Knit** button a document will be generated that includes both content as well as the output of any embedded R code chunks within the document. You can embed an R code chunk like this:

````
```{r 载入需要的程序包}
library(knitr)
library(xtable)
library(captioner)
library(kableExtra)
library(ggplot2)
library(RColorBrewer)
library(reshape2)
library(Seurat)
library(dplyr)
library(tidyr)
library(data.table)
library(sctransform)
library(limma)

Skinsheet <-readRDS(file="E:/皮片对比文章/Skinsheet_Seurat.rds") #单细胞


saveRDS(Skinsheet,file="F:/R_analysis/Vitiligo_Skinsheet.RDS")

Skinsheet <-readRDS(file="F:/皮片对比文章/Skinsheet_Seurat.rds") #单细胞

Skinsheet_KC <- readRDS(file="E:/皮片对比文章/keratinocyte_seurat.rds")
Skinsheet <- subset(Skinsheet, idents = c( "0","1","2","3","6","7",
"8","10","12","13","15","16",
"17","18","19","24","27","29"))




```


```{r 载入数据}
#查看不同特征之间的关系
plot1 <- FeatureScatter(Skinsheet,feature1="nCount_RNA",
                        feature2="percent.mt")+NoLegend()
plot2 <- FeatureScatter(Skinsheet,feature1="nCount_RNA",
                        feature2="nFeature_RNA")+NoLegend()
CombinePlots(plots=list(plot1,plot2))

ggsave("Skinsheet_compare_0.3_FeatureScatter.pdf",path="F:/皮片对比文章/0.3",width = 9,height= 7,
device="pdf")

```


```{r 载入数据}
#过滤数据：根据样本的特点调整参数
Skinsheet <- subset(Skinsheet,subset=nFeature_RNA>200 &
                 nFeature_RNA<10000 &
                 percent.mt<10)
````
````

````r
Skinsheet <- subset(Skinsheet,subset=nFeature_RNA>200 &
                  nFeature_RNA<10000 &
                  percent.mt<10)

VlnPlot(Skinsheet,
        features = c("nFeature_RNA",
                     "nCount_RNA",
                     "percent.mt"),
        ncol = 3)

```


```{r 载入数据}
#通过使用SCTransform进行数据标准化，实测比Seruat包旧算法好用
Skinsheet <- SCTransform(Skinsheet, vars.to.regress = "percent.mt", verbose = FALSE)
#使用该函数，等于同时进行了NormalizeData, ScaleData, 和 FindVariableFeatures三个命令

#线性降维sctransform
Skinsheet <- RunPCA(Skinsheet,verbose = FALSE)

# 基于PCA空间中的欧式距离计算nearest neighbor graph，优化任意两个细胞间的距离权重;
Skinsheet <- FindNeighbors(Skinsheet,reduction="harmony", dims = 1:30) # 前30个PC

#接着优化模型，resolution参数决定下游聚类分析得到的分群数，对于3k左右的细胞，设为0.4-1.2能得到较好的结果（官方说明）；如果数据量增大，该参数也应该适当增大;
Skinsheet <- FindClusters(Skinsheet,reduction="harmony", resolution = 0.3) # 分辨率是0.5


DimPlot(Skinsheet, reduction = "umap", label = TRUE, pt.size = 1) + NoLegend()


DimPlot(Skinsheet, reduction = "umap", split.by="seurat_clusters", ncol=5, label = TRUE, pt.size = 1) + NoLegend()

saveRDS(Skinsheet, file = "E:/R_analysis/Skinsheet_harmony.rds")

plot <- DimPlot(Skinsheet, reduction = "umap", split.by="orig.ident",label = TRUE,ncol=4, pt.size = 1) + NoLegend()
plot


setwd("D:/白癜风的单细胞+空转文章/Harmony")

png(filename = "Skinsheet_UMAP_by_Sample.png",width =10000,height =1500,res = 300)
print(plot)
dev.off()

library(clustree)

aa <- clustree(Skinsheet@meta.data, prefix = "SCT_snn_res.")


pdf(file = "E:/皮片对比文章/Clustree.pdf", width = 15, height = 10)
print(aa)
dev.off()

plot <- DimPlot(Skinsheet, reduction = "umap", split.by="orig.ident",label = TRUE,ncol=4, pt.size = 1) + NoLegend()
plot

ggsave("Skinsheet_compare_0.3_UMAP_SAMPLE.pdf",path="F:/皮片对比文章/0.3",width = 20,height= 5, device="pdf")




FeaturePlot(Skinsheet, features = c("nFeature_RNA",
                     "nCount_RNA",
                     "percent.mt"), cols = if (blend) {     c("lightgrey", "#ff0000", "#00ff00") }
````

````
else {
    c("lightgrey", "red") })

ggsave("Skinsheet_compare_0.3_FeaturePlot.pdf",path="F:/皮片对比文章/0.3",width = 20,height= 5,
device="pdf")

```


```{r 载入数据}
#展示前12个pca
print(Skinsheet[["pca"]],dims=1:15,nfeatures = 10)

# 绘制PCA散点图;
DimPlot(Skinsheet,reduction = "pca")+NoLegend()

#绘制前15个PCA
DimHeatmap(Skinsheet, dims = 1,
           cells = 500, balanced = TRUE)
DimHeatmap(Skinsheet, dims = 1:15,
           cells = 500, balanced = TRUE)

ggsave("Skinsheet_compare_0.3_PCA15.pdf",path="F:/皮片对比文章/0.3",width = 12,height= 20,
device="pdf")

```



```{r 载入数据}

#确定分群个数2ElbowPlot
aaa <- ElbowPlot(Skinsheet)
aaa
png(filename = "Skinsheet_Elbow.png",width =3000,height =1500,res = 300)
print(aaa)
dev.off()


umapplot <- DimPlot(Skinsheet, reduction = "umap", pt.size=1,by)
??umapplot
```

```{r 载入数据}

# 使用Umap，需要安装:
##reticulate::py_install(packages ="umap-learn")
Skinsheet <- RunUMAP(Skinsheet, reduction="pca", 1:30)
umapplot <- DimPlot(Skinsheet, reduction = "umap", pt.size=1)
umapplot
saveRDS(Skinsheet, file = "E:/皮片对比文章/Skinsheet_Seurat.rds")

Skinsheet <- readRDS("E:/皮片对比文章/Skinsheet_Seurat.rds")

#自定义亚群的名字
new.cluster.ids <- c("Keratinocyte_suprabasal_1",
"Keratinocyte_spinous_1",
"Keratinocyte_basal_KRT15",
"Keratinocyte_granular",
"Keratinocyte_granular",
"Keratinocyte_basal_LAMB3",
"Endothelial_cells",
"Fibroblast_DCN",
"Melanocyte",
"Smooth_Muscle_cells",
"Fibroblast_ZNF90",
"Langerhans_cells",
"Keratinocyte_suprabasal_2",
"T_cells",
"Keratinocyte_proliferation",
"Mast_cells",
````

```
"Neurons"
)

names(new.cluster.ids) <- levels(Skinsheet)

Skinsheet <- RenameIdents(Skinsheet, new.cluster.ids)

# 非必要names(fb@meta.data)[12] ='Cell_type_0.1'#改名字
#修改meta.data里面的亚群名称
Skinsheet@meta.data$seurat_clusters <-factor(Skinsheet@meta.data$seurat_clusters,
label=c("Keratinocyte_suprabasal_1",
"Keratinocyte_spinous_1",
"Keratinocyte_basal_KRT15",
"Keratinocyte_granular",
"Keratinocyte_granular",
"Keratinocyte_basal_LAMB3",
"Endothelial_cells",
"Fibroblast_DCN",
"Melanocyte",
"Smooth_Muscle_cells",
"Fibroblast_ZNF90",
"Langerhans_cells",
"Keratinocyte_suprabasal_2",
"T_cells",
"Keratinocyte_proliferation",
"Mast_cells",
"Neurons"))

Skinsheet@meta.data$SCT_snn_res.0.3 <-factor(Skinsheet@meta.data$SCT_snn_res.0.3,
label=c("Keratinocyte_suprabasal_1",
"Keratinocyte_spinous_1",
"Keratinocyte_basal_KRT15",
"Keratinocyte_granular",
"Keratinocyte_granular",
"Keratinocyte_basal_LAMB3",
"Endothelial_cells",
"Fibroblast_DCN",
"Melanocyte",
"Smooth_Muscle_cells",
"Fibroblast_ZNF90",
"Langerhans_cells",
"Keratinocyte_suprabasal_2",
"T_cells",
"Keratinocyte_proliferation",
"Mast_cells",
"Neurons"))

DimPlot(Skinsheet, reduction = "umap", label = TRUE, pt.size = 1) + NoLegend()

plot <- DimPlot(Skinsheet, reduction = "umap", split.by="orig.ident",ncol=2, label = TRUE, pt.size =
1) + NoLegend()
plot

setwd("E:/皮片对比文章")

png(filename = "Skinsheet_UMAP_harmony_bySample.png",width =4000,height =4000,res = 300)
print(plot)
dev.off()
```

```{r 载入数据}
# 用tsne的方法，并可视化
Skinsheet <- RunTSNE(Skinsheet,dims=1:30)
```

````
tsneplot <- TSNEPlot(Skinsheet,label=TRUE,
                         pt.size=1.5)+NoLegend()
tsneplot

setwd("E:/皮片对比文章")

png(filename = "Skinsheet_tSNE_harmony.png",width =2000,height =2000,res = 300)
print(tsneplot)
dev.off()



```



```{r 载入数据}
#保存运算结果，方便下次用
saveRDS(dim, file = "E:/R_analysis/ESdimALL_MC.rds")
```



```{r 载入数据}
#绘制某些基因的表达
FeaturePlot(Skinsheet,features = c("KRT15"))

# find all markers of cluster 2
cluster3.markers <- FindMarkers(Skinsheet, ident.1 = "Keratinocyte_granular1",
ident.2="Keratinocyte_granular2", min.pct = 0.4)

setwd("E:/皮片对比文章")
write.csv(cluster19.markers,"cluster24_markers.csv")
```



```{r 载入数据}
#计算所有markergenes
# find markers for every cluster compared to all remaining cells,
# # 计算一次差异基因不容易，我们保存在Seurat里。

Skinsheet.markers <- Skinsheet@misc$cluster_markers_all <- Seurat::FindAllMarkers(object =
Skinsheet,
                                              assay = "SCT",
                                              slot = "data",
                                              verbose = TRUE,
                                              only.pos = TRUE,
                                              logfc.threshold = 0.25,
                                              min.pct = 0.25)

Skinsheet.markers <- Skinsheet@misc$cluster_markers_all

Skinsheet.markers$cluster <-factor(Skinsheet.markers$cluster, label=c("Keratinocyte_suprabasal_1",
"Keratinocyte_spinous_1",
"Keratinocyte_basal_KRT15",
"Keratinocyte_granular",
"Keratinocyte_granular",
"Keratinocyte_basal_LAMB3",
"Endothelial_cells",
"Fibroblast_DCN",
"Melanocyte",
"Smooth_Muscle_cells",
"Fibroblast_ZNF90",
"Langerhans_cells",
"Keratinocyte_suprabasal_2",
"T_cells",
"Keratinocyte_proliferation",
"Mast_cells",
"Neurons"))

write.csv(Skinsheet.markers,"Skinsheet_markergenes_0.2.csv", row.names = T)
````

````
setwd("D:/胚胎干细胞向黑素细胞分化文章/Harmony")
write.csv(Skinsheet.markers,"Skinsheet_markergenes0.3.csv", row.names = T)

#保存运算结果，方便下次用
saveRDS(Skinsheet, file = "E:/R_analysis/Skinsheet_harmony.rds")

```


```{r 载入数据}
#小提琴图使用raw count
v <- Seurat::VlnPlot(Skinsheet, features = c("CTSC"),
              slot = "counts",
              log = TRUE)
v

```


```{r 载入数据}
#聚合小提琴图,基于ggplot2和Seurat，好用

features <- c("KRT15","RORA","DSC3","KRT1","KRT10","KRT5","KRT14","KRT2","PHLDA1","FST")

features <- subset(top10,cluster=="Neurons")$gene

a <- VlnPlot(Skinsheet, features, stack = TRUE, sort = TRUE,flip = TRUE) +
        theme(legend.position = "none") + ggtitle("")+xlab(' ')+
   theme(axis.text.x = element_text(size = 12,color = "black",
                                    face = "bold",
                                    angle= 45, vjust=1))+  #x轴字体格式
  theme(axis.text.y = element_text(size = 12,color = "black"))
a

setwd("E:/皮片对比文章")

png(filename = "Skinsheet_0.3_Violinplot_Neurons.png",width =4500,height =3000,res = 300)
print(a)
dev.off()

```


```{r 载入数据}
# cluster 图展示
FeaturePlot(dim,features = c("CD4", "CD8A", "CD8B","HLA-
DRB5","CCL5","CD52","CD2","TNFRSF4","TNFRSF18","ENO1","PKM","GNLY"
                                     ))
```




```{r}

#DoHeatmap为给定的细胞和特征生成一个表达式heatmap。在本例中，绘制每个集群的前20个标记(如果小于20，则绘制所有标记
 Skinsheet.markers <- read.csv(file="D:/白癜风的单细胞+空转文章/Harmony/Skinsheet_markergenes_0.2.csv")

top10 <- Skinsheet.markers%>%
         group_by(cluster) %>%
         top_n(n = 10, wt = avg_log2FC)
p <- DoHeatmap(Skinsheet, assay = "SCT", features = top10$gene) + NoLegend()
p
top10$cluster <-factor(top10$cluster, label=c("Keratinocyte_suprabasal_1",
"Keratinocyte_spinous_1",
"Keratinocyte_basal_KRT15",
"Keratinocyte_granular1",
"Keratinocyte_granular2",
"Keratinocyte_basal_LAMB3",
"Endothelial_cells",
````

````
"Fibroblast_DCN",
"Melanocyte",
"Smooth_Muscle_cells",
"Fibroblast_ZNF90",
"Langerhans_cells",
"Keratinocyte_suprabasal_2",
"T_cells",
"Keratinocyte_proliferation",
"Mast_cells",
"Neurons"))

write.csv(top10,"E:/皮片对比文章/Top10.markers.csv")

setwd("D:/白癜风的单细胞+空转文章/Harmony")

png(filename = "E:/皮片对比文章/Skinsheet_Proportion_Heatmap_0.3.png",width =8000,height =8000,res =
300)
print(p)
dev.off()
```

```{r}
#整理umap数据准备用ggplot2画barplot
umap_t = Skinsheet@reductions$umap@cell.embeddings %>%  #坐标信息
  as.data.frame() %>%
  cbind(cell_type = Skinsheet@meta.data$orig.ident) # 注释后的label信息 ，改为cell_type

umap_s = Skinsheet@meta.data$orig.ident  %>%
   as.data.frame() %>%
  cbind(cell_type = Skinsheet@active.ident) # 注释后的label信息 ，改为cell_type

names(umap_s) <- c("Sample","Cluster")
colnames(umap_s) <- c("Sample","Cluster")

setwd("E:/皮片对比文章/")
write.csv(umap_s, "Skinsheet_Barcode_Sample_Cluster0.3.csv", row.names = T)

setwd("E:/皮片对比文章/")
write.csv(umap_t, "Skinsheet_umap0.3.csv", row.names = F)

```

```{r}

library(ggplot2)
library(RColorBrewer)
library(ggrepel)


umap_t <- umap_t %>%
  mutate(cell_type = recode(cell_type, "Normal1" = "Normal", "Normal2" = "Normal"))


umap <- umap_t #把data改名为umap



allcolour=c("Keratinocyte_suprabasal_1"="#B5A77F",
"Keratinocyte_spinous_1"="#fab1a0",
"Keratinocyte_basal_KRT15"="#6F848F",
"Keratinocyte_granular"= "#A29EA1",
"Keratinocyte_basal_LAMB3"= "#F8E3D0",
"Endothelial_cells"= "#E2CAB2",
"Fibroblast_DCN" = "#6F0B1B",
"Melanocyte"= "#38323B",
"Smooth_Muscle_cells"= "#A17B90",
"Fibroblast_ZNF90"= "#E6495E",
"Langerhans_cells"= "#40DB71",
"Keratinocyte_suprabasal_2" = "#1B6C8F",
````

````
"T_cells"= "#6ab04c",
"Keratinocyte_proliferation" =  "#404EDB",
"Mast_cells" = "#129CA6",
"Neurons" = "#F27436") #设置颜色合集

p <- ggplot(umap,aes(x= UMAP_1 , y = UMAP_2, color = cell_type)) +  #设置参数
  geom_point(size = 1 , alpha =1)    #画UMAP点图
  #scale_color_manual(values = allcolour)  #设置颜色为allcolour颜色合集
p

##---------------------------------
p2 <- p  +
theme(panel.grid.major = element_blank(), #主网格线
        panel.grid.minor = element_blank(), #次网格线
        panel.border = element_blank(), #边框
        axis.title = element_blank(),  #轴标题
        axis.text = element_blank(), # 文本
        axis.ticks = element_blank(),
        panel.background = element_rect(fill = 'white'), #背景色
        plot.background=element_rect(fill="white"))
p2 #展示p2
#-------------------------------------
p3 <- p2 +
  theme(
    legend.title = element_blank(), #去掉legend.title
    legend.key=element_rect(fill='white'), #
    legend.text = element_text(size=10), #设置legend标签的大小
    legend.key.size=unit(0.5,'cm') ) +  # 设置legend标签之间的大小
  guides(color = guide_legend(override.aes = list(size=5))) #设置legend中图例的大小
p3 #展示p3
###-------------------------------------------

cell_type_med <- umap %>%
  group_by(cell_type) %>%
  summarise(
    UMAP_1 = median(UMAP_1),
    UMAP_2 = median(UMAP_2))  #计算每个cluster的median 坐标位置

##-------------------------------------

d <- p3 + geom_label_repel(aes(label=cell_type),

                   size=3,
                   fontface="bold",
                   data = cell_type_med,

                    point.padding=unit(1.5, "lines")) +
           theme(legend.position = "none") #去掉图例

d

setwd("D:/皮片对比文章")
png(filename = "Skinsheet_UMAP.png", width =2000, height =1500,res = 300)
print(d)
dev.off()
```


```{r}
#统计Cluster和Sample对应的数量
f2 <- with(umap_s, xtabs(~Cluster+Sample))
f2

#输出数据
setwd("E:/皮片对比文章")
write.csv(f2, "Skinsheet_combine_cell_number0.3.csv", row.names = T)
```

```{r}
````

```
data3 <- read.csv(file="E:/皮片对比文章/Skinsheet_combine_cell_number0.3_Long.csv")

# data3$Sample <- factor(data3$Sample,levels=c())


barplot <- ggplot(data=data3, aes(x=Sample, y=Freq, fill=Cluster ))+
              geom_bar(position="stack", stat="identity",width=0.8, alpha=0.9) +
scale_fill_manual(values=c("Keratinocyte_suprabasal_1"="#B5A77F",
"Keratinocyte_spinous_1"="#fab1a0",
"Keratinocyte_basal_KRT15"="#6F848F",
"Keratinocyte_granular"= "#A29EA1",
"Keratinocyte_basal_LAMB3"= "#F8E3D0",
"Endothelial_cells"= "#E2CAB2",
"Fibroblast_DCN" = "#6F0B1B",
"Melanocyte"= "#38323B",
"Smooth_Muscle_cells"= "#A17B90",
"Fibroblast_ZNF90"= "#E6495E",
"Langerhans_cells"= "#40DB71",
"Keratinocyte_suprabasal_2" = "#1B6C8F",
"T_cells"= "#6ab04c",
"Keratinocyte_proliferation" =  "#404EDB",
"Mast_cells" = "#129CA6",
"Neurons" = "#F27436"))+
                ylab('')+xlab(' ')+ # xy轴标题
                theme(axis.text.x = element_text(size = 25,color = "black", face = "bold", angle=
45, vjust=1,
                                                 hjust=1)) + #x轴字体格式
                theme(axis.text.y = element_text(size = 25,color = "black")) + #y轴字体格式
                theme(panel.background = element_rect(fill = 'white', colour = 'black')) + #背景格式
                theme(legend.title = element_text(colour="black", size=20, face="bold")) + #图例标题
格式
                theme(legend.text = element_text(colour="black", size=20, face="bold")) + #图例内容
格式
                theme(legend.key.width=unit(1.5,"cm"), legend.key.size = unit(1.5, "cm"))+ #图例的宽
度
                scale_y_continuous(expand = c(0,0.5))+  #y轴留白
                scale_x_discrete(expand = c(0,0.5))+     #X轴留白
                theme(legend.margin=margin(b = -2, unit='cm'))  #图例间隔


barplot
setwd("E:/皮片对比文章")

png(filename = "Skinsheet_Proportion_Barplot_stack.png",width =4000,height =4500,res = 300)
print(barplot)
dev.off()
```


```{r}
keratinocyte <- subset(Skinsheet, idents = c("Keratinocyte_suprabasal_1",
"Keratinocyte_spinous_1",
"Keratinocyte_basal_KRT15",
"Keratinocyte_granular",
"Keratinocyte_basal_LAMB3",
"Keratinocyte_suprabasal_2",
"Keratinocyte_proliferation"
))

saveRDS(keratinocyte,"E:/皮片对比文章/keratinocyte_seurat.rds")
keratinocyte <- readRDS("E:/皮片对比文章/keratinocyte_seurat.rds")

fibroblast <- subset(Skinsheet, idents = c("Fibroblast_DCN","Fibroblast_ZNF90"

))

saveRDS(keratinocyte,"E:/皮片对比文章/fibroblast_seurat.rds")
```

````
SMC <- subset(Skinsheet, idents = c("Smooth_Muscle_cells"

))

saveRDS(keratinocyte,"E:/皮片对比文章/SMC_seurat.rds")


mc <- subset(Skinsheet, idents = c("Melanocyte"
))

saveRDS(mc,"E:/皮片对比文章/Melanocyte_seurat.rds")

```

```{r}


##For Cell

Meta.data <- subset(Skinsheet@meta.data, orig.ident=c("Normal1"))

Meta.data$Barcode <- rownames(Meta.data)

Normal1 <-subset(Meta.data,orig.ident=="Normal1")

Skinsheet_New <- subset(Meta.data,orig.ident=="Skinsheet_New")

Skinsheet_Old <- subset(Meta.data,orig.ident=="Skinsheet_Old")

N_S_N <- rbind(Normal1,Skinsheet_New)
N_S_N_1 <- N_S_N[,c("seurat_clusters","Barcode")]

N_S_O <- rbind(Normal1,Skinsheet_Old)
N_S_O_1 <- N_S_O[,c("seurat_clusters","Barcode")]


N_S_N <- subset(Skinsheet, cells=N_S_N_1$Barcode)
N_S_O <- subset(Skinsheet, cells=N_S_O_1$Barcode)

#Prepare Meta.data File
meta_data <- cbind(rownames(N_S_N@meta.data), N_S_N@meta.data[,'seurat_clusters', drop=F])
write.csv(meta_data, 'E:/皮片对比文章/cellphonedb_meta_N_S_N.csv')

#Prepare count File
write.csv(as.matrix(N_S_N@assays$RNA@data), 'E:/皮片对比文章/cellphonedb_count_N_S_N.csv')

#Prepare Meta.data File
meta_data <- cbind(rownames(N_S_O@meta.data), N_S_O@meta.data[,'seurat_clusters', drop=F])
write.csv(meta_data, 'E:/皮片对比文章/cellphonedb_meta_N_S_O.csv')

#Prepare count File
write.csv(as.matrix(N_S_O@assays$RNA@data), 'E:/皮片对比文章/cellphonedb_count_N_S_O.csv')

```


```{R}
#Cellphone online sbatch file

#!/bin/bash
#SBATCH -p hebhcnormal02
##SBATCH -N 1
#SBATCH -n 32
#SBATCH -J Cellphone_Normal
#SBATCH -o ./output.%j.o
#SBATCH -e ./output.%j.e
#SBATCH --mail-type=ALL
#SBATCH --mail-user=969358560@qq.com
````

````
#SBATCH --exclusive

source ~/miniconda3/etc/profile.d/conda.sh
conda activate Cellphone_env

cellphonedb method statistical_analysis ~/Cellphone/cellphonedb_meta_N_S_N.csv
~/Cellphone/cellphonedb_count_N_S_N.csv  --project-name=Cellphone_Normal_with_Skinsheet_New  --
counts-data=gene_name


cellphonedb method statistical_analysis  ~/Cellphone/cellphonedb_meta_N_S_O.csv
~/Cellphone/cellphonedb_count_N_S_O.csv  --project-name=Cellphone_Normal_with_Skinsheet_Old  --
counts-data=gene_name

```


```{r}

library(Seurat)
library(tidyverse)
library(GSVA)
library(msigdbr)
library(pheatmap)
library(patchwork)
library(limma)

##创建gmt文件转list函数
gmt2list <- function(gmtfile){
  sets <- as.list(read_lines(gmtfile))
  for(i in 1:length(sets)){
    tmp = str_split(sets[[i]], '\t')
    n = length(tmp[[1]])
    names(sets)[i] = tmp[[1]][1]
    sets[[i]] = tmp[[1]][3:n]
    rm(tmp, n)
  }
  return(sets)
}

#读取数据
Skinsheet <- readRDS("~/Skinsheet_Seurat.rds")


##读取基因集数据库
s.sets = gmt2list("./c2.cp.v7.5.1.symbols.gmt")
###------------------------------------------------------
#subset file
KC.list = c(
  "Keratinocyte_suprabasal_1",
"Keratinocyte_spinous_1",
"Keratinocyte_basal_KRT15",
"Keratinocyte_granular",
"Keratinocyte_granular",
"Keratinocyte_basal_LAMB3",
"Endothelial_cells",
"Fibroblast_DCN",
"Melanocyte",
"Smooth_Muscle_cells",
"Fibroblast_ZNF90",
"Langerhans_cells",
"Keratinocyte_suprabasal_2",
"T_cells",
"Keratinocyte_proliferation",
"Mast_cells",
"Neurons"
)

step <- function(i, KC.list)
{
````

```
  kci=KC.list[[i]]
  xx <-subset(Skinsheet, ident=kci)
  #构建GSVA矩阵完成分析
  expr <- as.matrix(xx@assays$RNA@counts)
  #保存的文件名
  fn1 <- paste0('./skinsheet/', kci ,'.csv')
  fn2 <- paste0('./skinsheet/', kci ,'_GSVA.csv')
  write.csv(expr, file=fn1, row.names = T)
  #输出结果
  es.matrix = gsva(expr, s.sets, kcdf="Poisson",parallel.sz=32)
  write.csv(es.matrix, file=fn2, row.names = T)
}

library(foreach)  #并行循环运行包
foreach(i=1:length(KC.list)) %do% step(i, KC.list)
```

```{r}
#monocle3轨迹分析
##创建CDS对象并预处理数据
library(monocle3)

data <- GetAssayData(Skinsheet_KC, assay = 'RNA', slot = 'counts')

cell_metadata <- Skinsheet_KC@meta.data

gene_annotation <- data.frame(gene_short_name = rownames(data))

rownames(gene_annotation) <- rownames(data)

#创建Monocle对象
cds <- new_cell_data_set(data,
                         cell_metadata = cell_metadata,
                         gene_metadata = gene_annotation)

#Step 1: Normalize and pre-process the data, preprocess_cds函数相当于seurat中
NormalizeData+ScaleData+RunPCA
cds <- preprocess_cds(cds, num_dim = 100)

## Step 2: Remove batch effects with cell alignment
#cds <- align_cds(cds, alignment_group = "batch")

### Step 3: Reduce the dimensions using UMAP
cds <- reduce_dimension(cds, preprocess_method = "PCA")
p1 <- plot_cells(cds, reduction_method="UMAP", color_cells_by="seurat_clusters") +
ggtitle('cds.umap')
p1

#去除批次效应
cds = align_cds(cds, num_dim = 100, alignment_group = "Sample")
cds = reduce_dimension(cds)
plot_cells(cds, color_cells_by="Sample", label_cell_groups=FALSE)

plot_cells(cds, genes=c("DCT", "MITF", "KRT6C", "KRT6A"))

### Step 3: Reduce the dimensions using UMAP
cds <- reduce_dimension(cds, preprocess_method = "PCA")
p1 <- plot_cells(cds, reduction_method="UMAP", color_cells_by="seurat_clusters") +
ggtitle('cds.umap')
p1

##从seurat导入整合过的umap坐标
cds.embed <- cds@int_colData$reducedDims$UMAP
int.embed <- Embeddings(Skinsheet_KC, reduction = "umap")
int.embed <- int.embed[rownames(cds.embed),]
cds@int_colData$reducedDims$UMAP <- int.embed
p2 <- plot_cells (cds, reduction_method="UMAP", color_cells_by="seurat_clusters") +
```

```r
ggtitle('int.umap')
p2


## Monocle3聚类分区
cds <- cluster_cells(cds)
p1 <- plot_cells(cds, show_trajectory_graph = FALSE) + ggtitle("label by clusterID")
p2 <- plot_cells(cds, color_cells_by = "partition", show_trajectory_graph = FALSE) +
  ggtitle("label by partitionID")
p = wrap_plots(p1, p2)
p


## 识别轨迹
cds <- learn_graph(cds)

allcoluor = c("#9ED54C","#f0932b","#fab1a0","#e17055")

p = plot_cells(cds,
           color_cells_by = "seurat_clusters",

           label_cell_groups=FALSE,
           label_leaves=TRUE,
           label_branch_points=TRUE,
           graph_label_size=5,
           cell_size = 2,
           alpha = 0.4,
           trajectory_graph_color= "grey28",
           trajectory_graph_segment_size = 1.75)
p
ggsave("keratinocyte_monocle3_umap.pdf",path="F:/皮片对比文章/Monocle3",width=16,height = 9,
device="pdf")

##细胞按拟时排序
cds <- order_cells(cds) #存在bug，使用辅助线选择root细胞

aaa<-plot_cells(cds, color_cells_by = "pseudotime", label_cell_groups = FALSE,
           label_leaves = FALSE,  label_branch_points = FALSE,cell_size = 2,
           graph_label_size=5,
           alpha = 0.8,
           trajectory_graph_color= "grey28",
           trajectory_graph_segment_size = 1.75) +
  scale_color_gradient2(mid = '#0067BD', high = '#00D62C')

aaa

# a helper function to identify the root principal points:
get_earliest_principal_node <- function(cds, seurat_clusters="Keratinocyte_basal_KRT15"){
  cell_ids <- which(colData(cds)[, "seurat_clusters"] == seurat_clusters)

  closest_vertex <-
  cds@principal_graph_aux[["UMAP"]]$pr_graph_cell_proj_closest_vertex
  closest_vertex <- as.matrix(closest_vertex[colnames(cds), ])
  root_pr_nodes <-
  igraph::V(principal_graph(cds)[["UMAP"]])$name[as.numeric(names
  (which.max(table(closest_vertex[cell_ids,]))))]

  root_pr_nodes
}

cds <- order_cells(cds, root_pr_nodes=get_earliest_principal_node(cds))

#plot again
aaa<-plot_cells(cds, color_cells_by = "pseudotime", label_cell_groups = FALSE,
           label_leaves = FALSE,  label_branch_points = FALSE,cell_size = 2,
           graph_label_size=5,
           alpha = 0.4,
           trajectory_graph_color= "grey28",
           trajectory_graph_segment_size = 1.75)
```

```
aaa
ggsave("keratinocyte_monocle3_umap_pseudotime.pdf",path="F:/皮片对比文章/Monocle3",width=16,height = 9, device="pdf")

###分支的基因表达
#Subset cells by branch
cds_KC <- choose_cells(cds)


##寻找拟时轨迹差异基因
#graph_test分析最重要的结果是莫兰指数（morans_I），其值在-1至1之间，0代表此基因没有
#空间共表达效应，1代表此基因在空间距离相近的细胞中表达值高度相似。
subset_pr_test_res <- graph_test(cds_KC, neighbor_graph="principal_graph", cores=4)

pr_deg_ids <- row.names(subset(subset_pr_test_res, q_value < 0.05))

write.csv(subset_pr_test_res,file="F:/皮片对比文章/Monocle3/SkinsheetKC_Monocle.csv")

subset_pr_test_res <- data.table::fread("F:/皮片对比文章/Monocle3/SkinsheetKC_Monocle.csv")

#计算主要的差异基因modules
gene_module_df <- find_gene_modules(cds_KC[pr_deg_ids,], resolution=0.001)

write.csv(gene_module_df,file="F:/皮片对比文章/Monocle3/SkinsheetKC_Monocle_module.csv")

gene_module_df <- data.table::fread("E:/皮片对比文章/Monocle3/SkinsheetKC_Monocle_module.csv")

agg_mat <- aggregate_gene_expression(cds_KC, gene_module_df)
module_dendro <- hclust(dist(agg_mat))
gene_module_df$module <- factor(gene_module_df$module,
                                levels = row.names(agg_mat)[module_dendro$order])

#绘制主要的modules图
modules.plot <- plot_cells(cds_KC,
  trajectory_graph_color = "grey28",
  trajectory_graph_segment_size = 1.75,
  graph_label_size=5,
           alpha = 0.8,
  cell_size = 2,
           genes=gene_module_df,
           label_cell_groups=F,
           show_trajectory_graph=FALSE) +
    scale_color_gradient2(mid = '#948D93', high = '#FF2525')

setwd("F:/皮片对比文章/Monocle3/")
png(filename = "SkinsheetKC_modules_Monocle.png",width =5000,height =9000,res = 400)
print(modules.plot)
dev.off()


#绘制modules的heatmap图
cell_group_df <- tibble::tibble(cell=row.names(colData(cds_KC)),
                                cell_group=colData(cds_KC)$seurat_clusters)

agg_mat <- aggregate_gene_expression(cds_KC, gene_module_df, cell_group_df)

row.names(agg_mat) <- stringr::str_c("Module ", row.names(agg_mat))

pheatmap <- pheatmap::pheatmap(agg_mat,
                   scale="column", clustering_method="ward.D2")


setwd("F:/皮片对比文章/Monocle3/")
png(filename = "heatmap_KC_modules.png",width =5000,height =3000,res = 400)
print(pheatmap)
dev.off()


```

````
```{r}
##寻找拟时轨迹差异基因
#graph_test分析最重要的结果是莫兰指数（morans_I），其值在-1至1之间，0代表此基因没有
#空间共表达效应，1代表此基因在空间距离相近的细胞中表达值高度相似。
#挑选top10画图展示

gene_module_df_select <- subset(gene_module_df, module==c('4','15'))

names(gene_module_df_select)[1] <- "gene_short_name"

#取两个数据集合并项交集
ids <- Reduce(intersect, list(gene_module_df_select$gene_short_name,
subset_pr_test_res$gene_short_name))

#将data1在data2没有对应项目的Barcode去除
subset_pr_test_res_select <- subset_pr_test_res[subset_pr_test_res$gene_short_name %in% ids,]




Track_genes_sig <- C("ITGB1","TPM1",
"LAMC2",
"LAMB3",
"VIM",
"SOX10",
"COL12A1",
"LGALS1",
"ITGA6",
"SERPINE1")

aaa <- plot_genes_in_pseudotime(cds_KC[Track_genes_sig,], color_cells_by="pseudotime",
                        min_expr=0.5, ncol = 2)
aaa
setwd("F:/皮片对比文章/Monocle3/")
png(filename = "KC_Monocle3_trajectory_GENES.png",width =4000,height =3000,res = 400)
print(aaa)
dev.off()

save.image(file = "F:/皮片对比文章/Monocle3/Monocle3_KC_final.Rdata")

load(file = "F:/皮片对比文章/Monocle3/Monocle3_KC_final.Rdata")

```


```{R}

##GSEA
library(GSEABase)
library(ReactomePA)
library(tidyverse)
library(data.table)
library(org.Hs.eg.db)
library(clusterProfiler)
library(biomaRt)
library(enrichplot)
library(DOSE)

gene_module_df <- data.table::fread("F:/皮片对比文章/Monocle3/SkinsheetKC_Monocle_module.csv")
gene.list1 <- subset(gene_module_df,module=="4")
gene.list2 <- subset(gene_module_df,module=="15")

gene.list <- rbind(gene.list1, gene.list2)

gene.list <- as.data.table(gene.list$id)
names(gene.list) <- "gene_short_name"
subset_pr_test_res <- data.table::fread("F:/皮片对比文章/Monocle3/SkinsheetKC_Monocle.csv")
#取两个数据集合并项交集
````
````

```
ids <- Reduce(intersect, list(subset_pr_test_res$gene_short_name, gene.list$gene_short_name))

#将data1在data2没有对应项目的Barcode去除
subset_pr_test_res <- subset_pr_test_res[subset_pr_test_res$gene_short_name %in% ids,]


#提取有效信息
genelist_input <- subset_pr_test_res[,c("gene_short_name","morans_I")]

genename <- as.character(genelist_input[,1])

#基因名变为ENTREZID格式
gene_map <- AnnotationDbi::select(org.Hs.eg.db, keys=ids, keytype="SYMBOL", columns=c("ENTREZID"))

#改一下第一列的名字
colnames(gene_map)[1]<-"gene_short_name"

#合并表格
aaa<-inner_join(gene_map,genelist_input,by = "gene_short_name")
aaa<-aaa[,-1]
aaa<-na.omit(aaa)
aaa$morans_I<-sort(aaa$morans_I,decreasing = T)

#准备GSEA样式文件
geneList = aaa[,2]
names(geneList) = as.character(aaa[,1])
geneList

#GSEA分析——GO
data(geneList)
de <- names(geneList)[abs(geneList) > 2]
edo <- enrichDGN(de)


barplot(edo, showCategory=20)

dotplot(edo2, showCategory=30) + ggtitle("dotplot for GSEA")


Go_gseresult <- gseGO(geneList, 'org.Hs.eg.db', keyType = "ENTREZID", ont="all", nPerm = 1000,
minGSSize = 10, maxGSSize = 1000, pvalueCutoff=1)

Go_gseresult_BP <- gseGO(geneList, 'org.Hs.eg.db', keyType = "ENTREZID", ont="BP", nPerm = 1000,
minGSSize = 10, maxGSSize = 1000, pvalueCutoff=1)

##

edox <- setReadable(Go_gseresult, 'org.Hs.eg.db', 'ENTREZID')

edox_BP <- setReadable(Go_gseresult_BP, 'org.Hs.eg.db', 'ENTREZID')

p1 <- cnetplot(edox, edox@result$ONTOLOGY=="BP", foldChange=geneList)
p1
ggsave("KC_LAMB3_MONOCLE3_module4&15_GO_cnetplot.pdf",path="f:/皮片对比文章/Monocle3",width = 12,
height= 9, device="pdf")

## categorySize can be scaled by 'pvalue' or 'geneNum'
p2 <- cnetplot(edox, categorySize="pvalue", foldChange=geneList)
ggsave("KC_LAMB3_MONOCLE3_module4&15_GO_cnetplot_2.pdf",path="f:/皮片对比文章/Monocle3",width = 12,
height= 9, device="pdf")


p3 <- cnetplot(edox, foldChange=geneList, circular = TRUE, colorEdge = TRUE)
ggsave("KC_LAMB3_MONOCLE3_module4&15_GO_cnetplot_3.pdf",path="f:/皮片对比文章/Monocle3",width = 12,
height= 9, device="pdf")

p4 <- heatplot(edox, foldChange=geneList, showCategory=5)
ggsave("KC_LAMB3_MONOCLE3_module4&15_GO_heatmap_1.pdf",path="f:/皮片对比文章/Monocle3",width = 18,
height= 2.5, device="pdf")
```

~~~
##TREE

edox2 <- pairwise_termsim(edox_BP)
p5 <- treeplot(edox2, hclust_method = "average", showCategory=30)
ggsave("KC_LAMB3_MONOCLE3_module4&15_GO_TREEPLOT_1.pdf",path="f:/皮片对比文章/Monocle3",width = 18,
height= 12, device="pdf")
``````


```{r}


gseaplot2(Go_gseresult, geneSetID = 1,title = "Shigellosis", color = "darkgreen")


go_GESA <- Go_gseresult@result

dotplot(Go_gseresult, showCategory=30) + ggtitle("dotplot for ORA")

#GSEA分析——KEGG
KEGG_gseresult <- gseKEGG(geneList, nPerm = 1000, minGSSize = 10, maxGSSize = 1000, pvalueCutoff=1)

#GSEA分析——Reactome
Go_Reactomeresult <- gsePathway(geneList, nPerm = 1000, minGSSize = 10, maxGSSize = 1000,
pvalueCutoff=1)

edox_Reactome <- setReadable(Go_Reactomeresult, 'org.Hs.eg.db', 'ENTREZID')

aa <- c("GPCR ligand binding","Integrin cell surface interactions","Antigen processing:
Ubiquitination & Proteasome degradation","Diseases of glycosylation","Degradation of the
extracellular matrix")

r1 <- heatplot(edox_Reactome, foldChange=geneList, showCategory=aa)
r1
ggsave("KC_MONOCLE3_module4$15_Reactome_heeatplot.pdf",path="f:/皮片对比文章/Monocle3",width = 16,
height= 3, device="pdf")


edox_Reactome_pairwise <- pairwise_termsim(edox_Reactome)
p5 <- treeplot(edox_Reactome_pairwise, hclust_method = "average",showCategory = 30)
ggsave("KC_LAMB3_MONOCLE3_module4&15_Reactome_TREEPLOT_1.pdf",path="f:/皮片对比文章/Monocle3",width
= 18, height= 12, device="pdf")

#保存文件
setwd("f:/皮片对比文章/Monocle3")
write.table (Go_gseresult, file ="KC_MONOCLE3_module4&15_Go_gseresult.csv", sep =",", row.names
=TRUE)

setwd("f:/皮片对比文章/Monocle3")
write.table (Go_Reactomeresult, file ="KC_MONOCLE3_module4&15_Go_Reactomeresult.csv", sep =",",
row.names =TRUE)

write.table (KEGG_gseresult, file ="KC_MONOCLE3_module2_KEGG_gseresult.csv", sep =",", row.names
=TRUE)

#ridgeplot
ridgeplot <- ridgeplot(Go_gseresult,10)
ggsave("KC_MONOCLE3_module2_GO_ridgeplot.pdf",path="F:/皮片对比文章/Monocle3",device="pdf")


#dotplot
dotplot(Go_gseresult)
ggsave("KC_LAMB3_MONOCLE3_module4&15_GO_dotplot.pdf",path="f:/皮片对比文章/Monocle3",device="pdf")

dotplot <- dotplot(Go_Reactomeresult,10)
ggsave("KC_MONOCLE3_module4$15_Reactome_dotplot.pdf",path="f:/皮片对比文章/Monocle3",device="pdf")
~~~

````
```

```{r}

##GSEA
library(Seurat)
library(ReactomePA)
library(tidyverse)
library(data.table)
library(org.Hs.eg.db)
library(clusterProfiler)
library(biomaRt)
library(enrichplot)
library(dplyr)
library(tidyr)

melanocyte <- readRDS("D:/skinsheet_3_Melanocyte.rds")

melanocyte.markers <- FindMarkers(melanocyte,group.by = "orig.ident",ident.1 =
"Skinsheet_New",ident.2 = "Skinsheet_Old",
                                  assay = "RNA",
                                  slot = "data",
                                  verbose = TRUE,
                                  only.pos = TRUE,
                                  logfc.thrmchold = 0.25,
                                  min.pct = 0.25)

#提取有效信息
melanocyte.markers$gene_short_name <- rownames(melanocyte.markers)

#melanocyte.markers.1 <- subset(melanocyte.markers, melanocyte.markers$p_val_adj<0.05)

genelist_input <- melanocyte.markers[,c("gene_short_name","avg_log2FC")]


#基因名变为ENTREZID格式
gene_map <- select(org.Hs.eg.db, keys=rownames(genelist_input), keytype="SYMBOL",
columns=c("ENTREZID"))

#改一下第一列的名字
colnames(gene_map)[1]<-"gene_short_name"

#合并表格
aaa<-inner_join(gene_map,genelist_input,by = "gene_short_name")
aaa<-aaa[,-1]
aaa<-na.omit(aaa)
aaa$avg_log2FC<-sort(aaa$avg_log2FC,decreasing = T)

#准备GSEA样式文件
geneList = aaa[,2]
names(geneList) = as.character(aaa[,1])
geneList

#GSEA分析——GO
Go_gseresult <- gseGO(geneList, 'org.Hs.eg.db', keyType = "ENTREZID", ont="all", nPerm = 1000,
minGSSize = 10, maxGSSize = 1000, pvalueCutoff=1)

#GSEA分析——KEGG
#KEGG_gseresult <- gseKEGG(geneList, nPerm = 1000, minGSSize = 10, maxGSSize = 1000, pvalueCutoff=1)

#GSEA分析——Reactome
Go_Reactomeresult <- gsePathway(geneList, nPerm = 1000, minGSSize = 10, maxGSSize = 1000,
pvalueCutoff=1)
````

```
select_GOenrichment <- function(Go_gseresult,number){
  filtered_data1 <- Go_gseresult@result %>%
    filter(ONTOLOGY %in% c("BP")) %>%
    arrange(p.adjust) %>%
    head(number)

  filtered_data2 <- Go_gseresult@result %>%
    filter(ONTOLOGY %in% c("CC")) %>%
    arrange(p.adjust) %>%
    head(number)

  filtered_data3 <- Go_gseresult@result %>%
    filter(ONTOLOGY %in% c("MF")) %>%
    arrange(p.adjust) %>%
    head(number)
  filtered_data4 <- rbind(filtered_data1, filtered_data2, filtered_data3)

}


data <- select_GOenrichment(Go_gseresult, 10)

write.csv(data,"D:/皮片对比文章/SFF_VS_SFD_GESA_GO.csv")

data1 <- Go_Reactomeresult@result

write.csv(data1,"D:/皮片对比文章/SFF_VS_SFD_GESA_Reactome.csv")

barplot_GOenrichment <- function(data, width, height,setwd,filename){
  # 绘制BP图
  plt_bp <- ggplot(subset(data, ONTOLOGY == "BP"), aes(x = enrichmentScore, y = Description, shape =
ONTOLOGY, size = NES, color = p.adjust)) + scale_shape_manual(values =16) +
    geom_point(alpha = 0.8) +
    labs(title = "BP Plot") +
    scale_color_viridis_c(option = "B", alpha = 0.8, direction = 1) +
    labs(x = "EnrichmentScore", y = "Description") +
    theme(axis.text.y = element_text(size = 16, face = "bold"))+
    theme(axis.text.x = element_text(size = 6)) +
    theme_bw() +  # 将输出图的背景改为白色，保留网格线
    theme(panel.grid.major = element_line(color = "gray", linetype = "dashed"),  # 设置主要网格线的
样式
          panel.grid.minor = element_blank() # 隐藏次要网格线
    )



  # 绘制CC图
  plt_cc <- ggplot(subset(data, ONTOLOGY == "CC"), aes(x = enrichmentScore, y = Description, shape =
ONTOLOGY, size = NES, color = p.adjust)) + scale_shape_manual(values =17) +
    geom_point(alpha = 0.8) +
    labs(title = "CC Plot") +
    scale_color_viridis_c(option = "B", alpha = 0.8, direction = 1)+
    labs(x = "EnrichmentScore", y = "Description") +
    theme(axis.text.y = element_text(size = 16, face = "bold"))+
    theme(axis.text.x = element_text(size = 6)) +
    theme_bw() +  # 将输出图的背景改为白色，保留网格线
    theme(panel.grid.major = element_line(color = "gray", linetype = "dashed"),  # 设置主要网格线的
样式
          panel.grid.minor = element_blank() # 隐藏次要网格线
    )

  # 绘制MF图
  plt_mf <- ggplot(subset(data, ONTOLOGY == "MF"), aes(x = enrichmentScore, y = Description, shape =
ONTOLOGY, size = NES, color = p.adjust)) + scale_shape_manual(values =18) +
    geom_point(alpha = 0.8) +
```

~~~~
    labs(title = "MF Plot") +
    scale_color_viridis_c(option = "B", alpha = 0.8, direction = 1)+
    labs(x = "EnrichmentScore", y = "Description") +
    theme(axis.text.y = element_text(size = 16, face = "bold"))+
    theme(axis.text.x = element_text(size = 6)) +
    theme_bw() +  # 将输出图的背景改为白色，保留网格线
    theme(panel.grid.major = element_line(color = "gray", linetype = "dashed"),  # 设置主要网格线的
样式
          panel.grid.minor = element_blank() # 隐藏次要网格线
    )




  plt <- aplot::plot_list(plt_bp, plt_cc, plt_mf, tag_levels='A')
  plt

  setwd(setwd)
  png(filename = filename, width = width,height = height,res = 400)
  print(plt)
  dev.off()
}




barplot_GOenrichment(data,
                     width= 9000,
                     height = 2000,
                     setwd = "D:/皮片对比文章",
                     filename = "MC_GSEA_GO_enrichment.png")


data1 <- Go_Reactomeresult@result %>%
  arrange(p.adjust) %>%
  head(10)


#REACTOME barplot
plt_reactome <- ggplot(data1, aes(x = enrichmentScore, y = Description, size = NES, color =
p.adjust)) +
  geom_point(alpha = 0.8) +
  scale_shape_manual(values =18)+
  labs(title = "Reactome Plot") +
  scale_color_viridis_c(option = "B", alpha = 0.8, direction = 1) +
  labs(x = "EnrichmentScore", y = "Description") +
  theme(axis.text.y = element_text(size = 16, face = "bold"))+
  theme(axis.text.x = element_text(size = 6)) +
  theme_bw() +  # 将输出图的背景改为白色，保留网格线
  theme(panel.grid.major = element_line(color = "gray", linetype = "dashed"),  # 设置主要网格线的样
式
        panel.grid.minor = element_blank() # 隐藏次要网格线
  )
plt_reactome
ggsave("MC_GSEA_Reactome_enrichment.pdf",path="D:/皮片对比文章",width=10,height = 6, device="pdf")

```


```{r}
library(limma)

#载入文件
melanocyte <- readRDS("D:/皮片对比文章/skinsheet_3_Melanocyte.rds")
info = c("orig.ident","seurat_clusters")
cell.info <- subset(melanocyte@meta.data,select=info)
cell.info$Barcode <- rownames(cell.info)

GSVA.list <- data.table::fread(file="D:/皮片对比文章/MC_cells_GSVA_GO.csv")
~~~~

```
write.csv(GSVA.list, "E:/皮片对比文章/GSVA/Fibroblast_combine_GSVA.csv")

GSVA.list <- read.csv("D:/皮片对比文章/MC_cells_GSVA_GO.csv",row.names=1)

GSVA.list.t <- as.data.frame(t(GSVA.list))
rownames(GSVA.list.t) <- rownames(cell.info)
GSVA.list.t$Barcode <- rownames(GSVA.list.t)

GSVA.LIST.Combine <- cbind(cell.info, GSVA.list.t)

write.csv(GSVA.LIST.Combine,file="E:/皮片对比文章/GSVA/Melanocyte_GSVA_combine.csv")

GSVA.list <- data.table::fread(file="E:/皮片对比文章/GSVA/Melanocyte_GSVA_combine.csv")


#删除不需要的列并转置
GSVA.list <- GSVA.list[,-1]
GSVA.list_t <- as.data.frame(t(GSVA.list[, -c(1:3) ]))
names(GSVA.list_t) <- GSVA.list$Barcode


F1 <- subset(GSVA.list, orig.ident=='Skinsheet_New' )
F1_t <- as.data.frame(t(F1[, -c(1:3) ]))
names(F1_t) <- F1$Barcode

F2 <- subset(GSVA.list, orig.ident=='Skinsheet_Old' )
F2_t <- as.data.frame(t(F2[, -c(1:3) ]))
names(F2_t) <- F2$Barcode

#合并需要分析的样本
sample <- rbind(F1,F2)
sample_t <- as.data.frame(t(sample[, -c(1:3) ]))
names(sample_t) <- sample$Barcode

eset=sample_t

char_columns <- sapply(eset, is.character)              # Identify character columns
data_chars_as_num <- eset                               # Replicate data
data_chars_as_num[ , char_columns] <- as.data.frame(   # Recode characters as numeric
  apply(data_chars_as_num[ , char_columns], 2, as.numeric))
sapply(data_chars_as_num, class)



aa <- sample[,c("orig.ident","Barcode")]

# #提取注释
# L1 <- Normal1[,c("orig.ident","Barcode")]
# R1 <- Normal2[,c("orig.ident","Barcode")]
# D1 <- Skinsheet_Old[,c("orig.ident","Barcode")]
# aa <- rbind(L1, R1, D1)

colnames(aa)=c("Target","FileName") #改名字方便分析

aa$Target <- with(aa, ifelse( grepl('Skinsheet_New',Target) , 'F1', Target ))

aa$Target <- with(aa, ifelse( grepl('Skinsheet_Old',Target) , 'F2', Target ))

#aa$Target <- with(aa, ifelse( grepl('Melanocyte_3',Target) , 'MM', Target ))


#构建矩阵
lev<-unique(aa$Target) #使用unique（）函数保留唯一组名

f <- factor(aa$Target, levels=lev)

design <- model.matrix(~0+f) #样本矩阵

colnames(design) <- lev #更改列名为levels名
```

```
cont.wt <- makeContrasts("F1-F2",
                         levels=design) # compare group set Front vs Second


#Limma分析
fit <- lmFit(data_chars_as_num, design)
fit2 <- contrasts.fit(fit, cont.wt)
fit3 <- eBayes(fit2)
tT <- topTable(fit3, coef=1, sort.by="logFC",number=Inf)

#tT=topTable(fit3, adjust="BH",sort.by="logFC",n=Inf)
#tT = subset(tT, select=c("adj.P.Val","P.Value","logFC"))
#colnames(tT)=c("FDR","P.Value","logFC")

## barplot
tT1 <- data.frame(id = row.names(tT),
                       t = tT$t, log2FC=tT$logFC,
                  adj.P.Val=tT$adj.P.Val,P.Value=tT$P.Value,AveExpr=tT$AveExpr,
                  B=tT$B)
# 去掉注释文件的开头，比如："HALLMARK_"
library(stringr)
library(dplyr)
#tT1$id <- str_replace(tT1 $id , "REACTOME_","")
# 新增一列 根据t阈值分类
tT1 $threshold = factor(ifelse(tT1$t  >-2, ifelse(tT1$t >= 2
,'Up','NoSignifi'),'Down'),levels=c('Up','Down','NoSignifi'))
# 排序
tT1 <- tT1 %>% arrange(t)
# 变成因子类型
tT1$id <- factor(tT1$id,levels = tT1$id)
#输出文件
setwd("D:/皮片对比文章")
write.csv(tT1,"GSVA_MC_NEW_vS_OLD_Limma_1_GO.csv")


library(ggplot2)
library(ggthemes)
# install.packages("ggprism")
library(ggprism)
library(limma)
library(stringr)
library(dplyr)
##选择子集
# 我们使用|logFC| > 0.5，padj < 0.05（矫正后P值）
foldChange = 0.5
padj = 0.05
## 筛选出所有差异基因的结果
setwd("E:/皮片对比文章/GSVA/")
tT_sig <- read.csv("挑选_C2_GSVA_MC_Skinsheet_New_vs_Old.csv", header = T)

#tT_sig$id <- str_replace(tT_sig$id , "REACTOME_","")

#tT_sig <- tT_sig[(tT_sig$adj.P.Val < padj & (tT_sig$logFC>foldChange | tT_sig$logFC < (-
foldChange))),]
#---------------------
## barplot
dat_plot <- data.frame(id = tT_sig$id,
                       t = tT_sig$t)
# 新增一列 根据t阈值分类
dat_plot$threshold = factor(ifelse(dat_plot$t  >-2, ifelse(dat_plot$t >= 2
,'Up','NoSignifi'),'Down'),levels=c('Up','Down','NoSignifi'))
# 排序
dat_plot <- dat_plot %>% arrange(t)
# 变成因子类型
dat_plot$id <- factor(dat_plot$id,levels = dat_plot$id)
# 绘制

p <- ggplot(data = dat_plot,aes(x = id,y = t,fill = threshold)) +
```

```
  geom_col()+
  coord_flip() +
  scale_y_continuous(limits = c(0, max(dat_plot$t)),expand = c(0.001, 0.001)) +
  scale_fill_manual(values = c('Up'= '#4c464e','NoSignifi'='#dfe6e9','Down'='#9ED54C')) +
  geom_hline(yintercept = c(-2,2),color = 'white',size = 1,lty='dashed') +
  xlab('') +
  ylab('t value of GSVA score, SFF versus SFD') + #注意坐标轴旋转了
  guides(fill=F)+ # 不显示图例
  labs(title="Reactome") + #标题
  theme(plot.title = element_text(hjust = 0.5)) + #title格式
  theme(title = element_text(size = 20, family = "myFont", color = "black", face = "bold"))+  #轴标
签
  theme(axis.title.y = element_text(size = 20, family = "myFont", color = "black", face = "bold"))+
#轴标签
  theme(axis.text.x = element_text(size = 20,color = "black", face = "bold")) +
  theme(axis.text.y = element_text(size = 20,color = "black")) + #轴显示字体大小
  theme(panel.background = element_rect(fill = 'white', colour = 'black'))


p

ggsave("挑选_GSVA_MC_SFF_VS_SFD_reactome_barplot.png",device="png", path="D:/皮片对比文章",width =
30, height = 5,limitsize = FALSE)

library(ggplot2)
library(dplyr)
library(patchwork)

#数据过滤前的原始的meta.data
meta.data.raw <- scRNA@meta.data


meta.data.raw$Barcode <- rownames(meta.data.raw)


#数据过滤后的meta.data
minGene =500
maxGene = 20000
maxUMI=15000
pctMT=10
pctHB=1

scRNA <- subset(scRNA,subset = nCount_RNA < maxUMI & nFeature_RNA >minGene &
                  nFeature_RNA < maxGene & percent.mt < pctMT & percent.HB < pctHB)

Barcode.name <- rownames(meta.data)

#合并两个数据标记过滤后的细胞

meta.data.raw$group[!meta.data.raw$Barcode %in% Barcode.name] <-"filter"

meta.data.raw$group[!meta.data.raw$Barcode %in% Barcode.name] <-"raw"



#根据表格作图
draw.1 <- meta.data.raw

#####fig1
plots_list <- lapply(unique(draw.1$orig.ident), function(id) {
  single_data <- subset(draw.1, orig.ident == id)

  p <- ggplot(single_data, aes(x=nCount_RNA, y=nFeature_RNA, color=group)) +
    geom_point(alpha=0.5) +
    geom_density_2d(colour="black", alpha=0.5) +
    labs(title = id,
         x = "Total counts", y = "Number of genes",
         colour = "Exclude cell") +
    scale_x_log10() +
```

````
    scale_y_log10()

  return(p)
})

combined_plot <- wrap_plots(plots_list, ncol = 3)
print(combined_plot)


ggsave("按指点文献绘制质控图1.pdf",path="D:/皮片对比文章/",width = 16,height= 12, device="pdf")


###fig2

plots_list <- lapply(unique(draw.1$orig.ident), function(id) {
  single_data <- subset(draw.1, orig.ident == id)

  p <- ggplot(single_data, aes(x=nCount_RNA, y=nFeature_RNA, color=percent.mt)) +
    scale_color_gradient(low = "yellow", high = "darkred") +
    geom_point(alpha=0.5) +
    geom_density_2d(colour="black", alpha=0.5) +
    labs(title = id,
         x = "Total counts", y = "Number of genes",
         colour = "Percentage\nMitochondrial\ncounts") +
    scale_x_log10() +
    scale_y_log10()

  return(p)
})

combined_plot <- wrap_plots(plots_list, ncol = 3)
print(combined_plot)


ggsave("按指点文献绘制质控图2.pdf",path="D:/皮片对比文章/",width = 16,height= 12, device="pdf")




```

```{r}


library(ggplot2)
library(dplyr)
library(patchwork)

#数据过滤前的原始的meta.data
meta.data.raw <- scRNA@meta.data


meta.data.raw$Barcode <- rownames(meta.data.raw)


#数据过滤后的meta.data
minGene =500
maxGene = 20000
maxUMI=15000
pctMT=10
pctHB=1

scRNA <- subset(scRNA,subset = nCount_RNA < maxUMI & nFeature_RNA >minGene &
                  nFeature_RNA < maxGene & percent.mt < pctMT & percent.HB < pctHB)

Barcode.name <- rownames(meta.data)

#合并两个数据标记过滤后的细胞
````

```
meta.data.raw$group[!meta.data.raw$Barcode %in% Barcode.name] <-"filter"

meta.data.raw$group[!meta.data.raw$Barcode %in% Barcode.name] <-"raw"



#根据表格作图
draw.1 <- meta.data.raw

#####fig1
plots_list <- lapply(unique(draw.1$orig.ident), function(id) {
  single_data <- subset(draw.1, orig.ident == id)

  p <- ggplot(single_data, aes(x=nCount_RNA, y=nFeature_RNA, color=group)) +
    geom_point(alpha=0.5) +
    geom_density_2d(colour="black", alpha=0.5) +
    labs(title = id,
         x = "Total counts", y = "Number of genes",
         colour = "Exclude cell") +
    scale_x_log10() +
    scale_y_log10()

  return(p)
})

combined_plot <- wrap_plots(plots_list, ncol = 3)
print(combined_plot)


ggsave("按指点文献绘制质控图1.pdf",path="D:/皮片对比文章/",width = 16,height= 12, device="pdf")


###fig2

plots_list <- lapply(unique(draw.1$orig.ident), function(id) {
  single_data <- subset(draw.1, orig.ident == id)

  p <- ggplot(single_data, aes(x=nCount_RNA, y=nFeature_RNA, color=percent.mt)) +
    scale_color_gradient(low = "yellow", high = "darkred") +
    geom_point(alpha=0.5) +
    geom_density_2d(colour="black", alpha=0.5) +
    labs(title = id,
         x = "Total counts", y = "Number of genes",
         colour = "Percentage\nMitochondrial\ncounts") +
    scale_x_log10() +
    scale_y_log10()

  return(p)
})

combined_plot <- wrap_plots(plots_list, ncol = 3)
print(combined_plot)


ggsave("按指点文献绘制质控图2.pdf",path="D:/皮片对比文章/",width = 16,height= 12, device="pdf")



```



```{r}


#####
```

```
cellphonedb method statistical_analysis cellphonedb_meta.txt cellphonedb_count.txt --iterations=10 --threads=2 --counts-data=gene_name

#（2）子项目文件夹
cellphonedb method analysis cellphonedb_meta.txt cellphonedb_count.txt --project-name=zxh_mc
# （3）设置输出路径
mkdir custom_folder
cellphonedb method statistical_analysis cellphonedb_meta.txt cellphonedb_count.txt --output-path=./zxh_mc
#（4） 二次抽样
cellphonedb method analysis cellphonedb_meta.txt cellphonedb_count.txt --subsampling --subsampling-log false --subsampling-num-cells 3000




####


library(tidyverse)
library(RColorBrewer)
library(scales)

pvalues=read.table("D:/Vitiligo_Article/Cellphone/Skinsheet/pvalues.txt",header = T,sep = "\t",stringsAsFactors = F)
pvalues=pvalues[,12:dim(pvalues)[2]]
statdf=as.data.frame(colSums(pvalues < 0.05))
colnames(statdf)=c("number")



statdf$indexb=str_replace(rownames(statdf),"^.*\\.","")
statdf$indexa=str_replace(rownames(statdf),"\\..*$","")
statdf$total_number=0

for (i in 1:dim(statdf)[1]) {
  tmp_indexb=statdf[i,"indexb"]
  tmp_indexa=statdf[i,"indexa"]
  if (tmp_indexa == tmp_indexb) {
    statdf[i,"total_number"] = statdf[i,"number"]
  } else {
    statdf[i,"total_number"] = statdf[statdf$indexb==tmp_indexb & statdf$indexa==tmp_indexa,"number"]+
      statdf[statdf$indexa==tmp_indexb & statdf$indexb==tmp_indexa,"number"]
  }
}

rankname=sort(unique(statdf$indexa))
statdf$indexa=factor(statdf$indexa,levels = rankname)
statdf$indexb=factor(statdf$indexb,levels = rankname)

statdf%>%ggplot(aes(x=indexa,y=indexb,fill=total_number))+geom_tile(width=0.8,
            height=0.8,lwd = 2,
            linetype = 4)+
  scale_fill_gradientn(colours = c("#f6f7eb","white","#ff0000"),limits=c(0,180))+
  scale_x_discrete("cluster 1")+
  scale_y_discrete("cluster 2")+
  theme_minimal()+coord_fixed()+
  theme(
    axis.text.x.bottom = element_text(hjust = 1, vjust = NULL, angle = 45),
    panel.grid = element_blank()
  )

ggsave(filename = "D:/Vitiligo_Article/Cellphone/Skinsheet_interaction.num.2.pdf",device = "pdf",width = 24,height = 15,units = c("cm"))

library(tidyverse)
library(RColorBrewer)
```

```r
library(scales)
library(igraph)

pvalues=read.table("D:/Vitiligo_Article/Cellphone/Repigment/pvalues.txt",header = T,sep =
"\t",stringsAsFactors = F)
pvalues=pvalues[,12:dim(pvalues)[2]]
statdf=as.data.frame(colSums(pvalues < 0.05))
colnames(statdf)=c("number")

statdf$indexb=str_replace(rownames(statdf),"^.*\\.","")
statdf$indexa=str_replace(rownames(statdf),"\\..*$","")
rankname=sort(unique(statdf$indexa))

A=c()
B=c()
C=c()
remaining=rankname
for (i in rankname[-6]) {
  remaining=setdiff(remaining,i)
  for (j in remaining) {
    count=statdf[statdf$indexa == i & statdf$indexb == j,"number"]+
      statdf[statdf$indexb == i & statdf$indexa == j,"number"]
    A=append(A,i)
    B=append(B,j)
    C=append(C,count)
  }
}

statdf2=data.frame(indexa=A,indexb=B,number=C)
statdf2=statdf2 %>% rbind(statdf[statdf$indexa==statdf$indexb,c("indexa","indexb","number")])
statdf2=statdf2[statdf2$number > 0,] #过滤掉值为0的观测

#设置节点和连线的颜色
color1=c( "#9ED54C","#29CB9D","#CEA438",
"#40DB71","#1B6C8F","#6ab04c",
            "#404EDB","#FF4040","#00A690",
"#B5A77F",
"#fab1a0",
"#6F848F",
"#00A690",
"#F8E3D0",
"#E2CAB2",
"#FF4040",
"#C8E6D3",
"#A17B90",
"#E6495E",
"#f0932b","#fab1a0","#e17055","#FF9C9C","#FFC6BD",
"#fd79a8","#dfe6e9","#99E5FC","#FF4040","#00A690"
)
names(color1)=rankname
color2=colorRampPalette(brewer.pal(9, "Reds")[3:7])(20) #将颜色分成多少份，取决于互作关系数目的最大
值
names(color2)=1:20 #每一份颜色用对应的数字命名

#做网络图
##下面的四行代码相对固定
net <- graph_from_data_frame(statdf2[,c("indexa","indexb","number")])
edge.start <- igraph::ends(net, es=igraph::E(net), names=FALSE)
group <-  cluster_optimal(net)
coords <- layout_in_circle(net, order = order(membership(group)))

E(net)$width <- E(net)$number / 100 #将数值映射到连线的宽度，有时还需要微调，这里除以2就是这个目的
E(net)$color <- color2[as.character(ifelse(E(net)$number > 20,20,E(net)$number))] #用前面设置好的颜
色赋给连线，颜色深浅对应数值大小
E(net)$label = E(net)$number #连线的标注
E(net)$label.color <- "black" #连线标注的颜色
V(net)$label.color <- "black" #节点标注的颜色
V(net)$color <- color1[names(V(net))] #节点的填充颜色，前面已经设置了；V(net)返回节点信息
```

```
#调整节点位置的线条角度
##如果没有这两行代码，节点位置的圆圈是向右的
loop.angle<-ifelse(coords[igraph::V(net),1]>0,-
atan(coords[igraph::V(net),2]/coords[igraph::V(net),1]),pi-
atan(coords[igraph::V(net),2]/coords[igraph::V(net),1]))
igraph::E(net)$loop.angle[which(edge.start[,2]==edge.start[,1])] <-
loop.angle[edge.start[which(edge.start[,2]==edge.start[,1]),1]]

#pdf("interaction.num.3.pdf",width = 6,height = 6)
plot(net,
     edge.arrow.size = 0, #连线不带箭头
     edge.curved = 0, #连线不弯曲
     vertex.frame.color = "black", #节点外框颜色
     layout = coords,
     vertex.label.cex = 1, #节点标注字体大小
     vertex.size = 10) #节点大小
#dev.off()




rm(list = ls())
getwd()


mypvals <- read.table("D:/Vitiligo_Article/Cellphone/Repigment/pvalues.txt",header = T,sep =
"\t",stringsAsFactors = F)

mymeans <- read.table("D:/Vitiligo_Article/Cellphone/Repigment/means.txt",header = T,sep =
"\t",stringsAsFactors = F)


# 我们重点看  CD14_Mono 和 FCGR3A_Mono  ，以及  Memory_CD4_T和Naive_CD4_T 的通讯情况。

kp = grepl(pattern = "Melanocyte", colnames(mypvals)) & grepl(pattern = "", colnames(mypvals))

table(kp)
pos = (1:ncol(mypvals))[kp]
choose_pvalues <- mypvals[,c(c(1,5,6,8,9),pos  )]
choose_means <- mymeans[,c(c(1,5,6,8,9),pos)]


logi <- apply(choose_pvalues[,5:ncol(choose_pvalues)]<0.05, 1, sum)

# 只保留具有细胞特异性的一些相互作用对
choose_pvalues <- choose_pvalues[logi>=0.5,]

# 去掉空值
logi1 <- choose_pvalues$gene_a != ""
logi2 <- choose_pvalues$gene_b != ""
logi <- logi1 & logi2
choose_pvalues <- choose_pvalues[logi,]

# 同样的条件保留choose_means
choose_means <- choose_means[choose_means$id_cp_interaction %in% choose_pvalues$id_cp_interaction,]

head(choose_means,1)
head(choose_pvalues,1)

dim(mypvals)

dim(choose_means)

dim(choose_pvalues)



# 将choose_pvalues和choose_means数据宽转长
library(tidyverse)
```

```
meansdf <- choose_means %>% reshape2::melt()
meansdf <- data.frame(interacting_pair = paste0(meansdf$gene_a,"_",meansdf$gene_b),
                      CC = meansdf$variable,
                      means = meansdf$value)
pvalsdf <- choose_pvalues %>% reshape2::melt()
pvalsdf <- data.frame(interacting_pair = paste0(pvalsdf$gene_a,"_",pvalsdf$gene_b),
                      CC = pvalsdf$variable,
                      pvals = pvalsdf$value)


# 合并p值和mean文件
pvalsdf$joinlab<- paste0(pvalsdf$interacting_pair,"_",pvalsdf$CC)
meansdf$joinlab<- paste0(meansdf$interacting_pair,"_",meansdf$CC)
pldf <- merge(pvalsdf,meansdf,by = "joinlab")
write.csv(pldf,"D:/Vitiligo_Article/Cellphone/Repigment/pldf_Melanocyte_Repigment.csv")

# dotplot可视化

summary((filter(pldf,means >0))$means)
head(pldf)
pcc =  pldf%>% filter(means >0) %>%
  ggplot(aes(CC.x,interacting_pair.x) )+
  geom_point(aes(color=means,size=-log10(pvals+0.0001)) ) +
  scale_size_continuous(range = c(0,3))+
  scale_color_gradient2(high="#348aa7", mid="#5dd39e", low ="#ffd6e0",midpoint = 10  )+
  theme_bw()+
  # scale_color_manual(values = rainbow(100))+
  theme(axis.text.x = element_text(angle = -45,hjust = 0, vjust = 1))
pcc

ggsave(filename = "D:/Vitiligo_Article/Cellphone/Repigment/MC_interaction_Repigment.pdf",device =
"pdf",width = 75,height = 80,units = c("cm"),limitsize = FALSE)


FB_MC_means<- read.table(file="D:/Vitiligo_Article/Cellphone/cellphone FB VS MC.txt")
FB_MC_means <- FB_MC_means$V1


head(choose_means,1)
head(choose_pvalues,1)

dim(mypvals)

dim(choose_means)

dim(choose_pvalues)

# 将choose_pvalues和choose_means数据宽转长
library(tidyverse)
meansdf <- choose_means %>% reshape2::melt()
meansdf <- data.frame(interacting_pair = paste0(meansdf$gene_a,"_",meansdf$gene_b),
                      CC = meansdf$variable,
                      means = meansdf$value)
pvalsdf <- choose_pvalues %>% reshape2::melt()
pvalsdf <- data.frame(interacting_pair = paste0(pvalsdf$gene_a,"_",pvalsdf$gene_b),
                      CC = pvalsdf$variable,
                      pvals = pvalsdf$value)

meansdf_choose <- meansdf[meansdf$interacting_pair == rep(FB_MC_means,
length(meansdf$interacting_pair)), ]

pvalsdf_choose <- meansdf[pvalsdf$interacting_pair == rep(FB_MC_means,
length(pvalsdf$interacting_pair)), ]




# 合并p值和mean文件
pvalsdf_choose$joinlab<- paste0(pvalsdf_choose$interacting_pair,"_",pvalsdf_choose$CC)
```

```
meansdf_choose$joinlab<- paste0(meansdf_choose$interacting_pair,"_",meansdf_choose$CC)
pldf <- merge(pvalsdf_choose,meansdf_choose,by = "joinlab")


summary((filter(pldf,means >0))$means)
head(pldf)
pcc =  subset_df%>% filter(means >0) %>%
  ggplot(aes(CC.x,interacting_pair.x) )+
  geom_point(aes(color=means,size=-log10(pvals+0.0001)) ) +
  scale_size_continuous(range = c(0,3))+
  scale_color_gradient2(high="#348aa7", mid="#5dd39e", low ="#ffd6e0",midpoint = 10  )+
  theme_bw()+
  # scale_color_manual(values = rainbow(100))+
  theme(axis.text.x = element_text(angle = -45,hjust = 0, vjust = 1))
pcc



ggsave(filename = "D:/Vitiligo_Article/Cellphone/Repigment/MC_FB_interaction_Repigment_1.pdf",device
= "pdf",width = 75,height = 80,units = c("cm"),limitsize = FALSE)
```
