# Supplementary material for: Single-cell sequencing reveals increased LAMB3-positive basal keratinocytes and ZNF90-positive fibroblasts in autologous cultured epithelium
Source: Commun Biol. 2024 Jan 10;7:79. doi: 10.1038/s42003-023-05747-5 (PMC10781733; doi:10.1038/s42003-023-05747-5)
Supplement: Supplementary file 11 — Reporting Summary [file 42003_2023_5747_MOESM11_ESM.pdf]

nature portfolio

Corresponding author(s): Prof. Jinhua Xu, Prof. Qi Zhang

Last updated by author(s): Dec 7, 2023

Reporting Summary

Nature Portfolio wishes to improve the reproducibility of the work that we publish. This form provides structure for consistency and transparency in reporting. For further information on Nature Portfolio policies, see our Editorial Policies and the Editorial Policy Checklist.

Statistics

For all statistical analyses, confirm that the following items are present in the figure legend, table legend, main text, or Methods section.

n/a	Confirmed	
☐	☒	The exact sample size (n) for each experimental group/condition, given as a discrete number and unit of measurement
☐	☒	A statement on whether measurements were taken from distinct samples or whether the same sample was measured repeatedly
☐	☒	The statistical test(s) used AND whether they are one- or two-sided<br>*Only common tests should be described solely by name; describe more complex techniques in the Methods section.*
☐	☒	A description of all covariates tested
☐	☒	A description of any assumptions or corrections, such as tests of normality and adjustment for multiple comparisons
☐	☒	A full description of the statistical parameters including central tendency (e.g. means) or other basic estimates (e.g. regression coefficient) AND variation (e.g. standard deviation) or associated estimates of uncertainty (e.g. confidence intervals)
☐	☒	For null hypothesis testing, the test statistic (e.g. F, t, r) with confidence intervals, effect sizes, degrees of freedom and P value noted<br>*Give P values as exact values whenever suitable.*
☒	☐	For Bayesian analysis, information on the choice of priors and Markov chain Monte Carlo settings
☐	☒	For hierarchical and complex designs, identification of the appropriate level for tests and full reporting of outcomes
☐	☒	Estimates of effect sizes (e.g. Cohen's d, Pearson's r), indicating how they were calculated

Our web collection on statistics for biologists contains articles on many of the points above.

Software and code

Policy information about availability of computer code

Data collection	1. The transcriptome sequencing was performed on the Illumina Novaseq 6000 platform with PE150 strategy, and around 500M data was generated for each sample.<br>2. Zeiss LSM 800 confocal microscope was used to acquire images.<br>3. BD LSRFortessa was used for image processing.
Data analysis	1. Cell Ranger performs data quality statistics on the raw data and compares the reference genomes to the Ensembl database. R software (4.1.3) and python (3.8.4) was used for the bioinformatic analysis.<br>2. For immunocytochemistry analysis, the images were analyzed with Zen software 2.3.<br>3. For flow cytometry, the images were analyzed with Flowjo version10.4.0.<br>4. For qPCR, the statistical results and graphs were analyzed with Prism 10.

For manuscripts utilizing custom algorithms or software that are central to the research but not yet described in published literature, software must be made available to editors and reviewers. We strongly encourage code deposition in a community repository (e.g. GitHub). See the Nature Portfolio guidelines for submitting code & software for further information.

Data

Policy information about availability of data

All manuscripts must include a data availability statement. This statement should provide the following information, where applicable:
- Accession codes, unique identifiers, or web links for publicly available datasets
- A description of any restrictions on data availability
- For clinical datasets or third party data, please ensure that the statement adheres to our policy

The gene sequencing data in this article has been deposited to https://www.ncbi.nlm.nih.gov/ as BioProject: PRJNA802332. All data, codes, and materials in the analysis can be provided to any researcher for the purposes of reproducing or extending the analysis by contact with the correspondence author too.

Human research participants

Policy information about studies involving human research participants and Sex and Gender in Research.

Reporting on sex and gender	Both male and female were considered to be included in the study. Numbers of participants with sex and gender were written in the results. According to the sex- and gender-based analyses performed previously, no difference in the efficacy rate was observed between male and female (see "reference 11").
Population characteristics	Patients with vitiligo, aged 14-85 years, male or female, who completed autologous cultured epithelium grafting and were followed up for at least 6 months.
Recruitment	The subjects were all from the dermatology department of Huashan Hospital who received autologous cultured epithelium grafting. Patients with stable vitiligo (defined as no progression of existing lesions, no appearance of new lesions, and no Koebner phenomenon for at least 12 months), an inadequate response to a variety of medical treatments for vitiligo, and willingness and ability to undergo treatment with ACEG under the two types of sheets. There are three situations in the process of informed consent: when the subjects come to the hospital for follow-up, they can sign the informed consent form on site. If the subject cannot come to the hospital, the witness shall be present and the subject shall be informed of the test content by telephone. The basic information and clinical data of the subject shall be used to obtain the subject's informed consent. If the subject cannot come to the hospital for follow-up or contact, apply for exemption from informed consent and promise to keep the patient's information strictly confidential, which does not involve commercial interests.
Ethics oversight	This study was approved by the Ethics Committee of Huashan Hospital, Fudan University (KY2020-698, KY2020-1137) and registered with chictr.org.cn (ChiCTR2100051405). All participants provided written informed consent.

Note that full information on the approval of the study protocol must also be provided in the manuscript.

Field-specific reporting

Please select the one below that is the best fit for your research. If you are not sure, read the appropriate sections before making your selection.

☒ Life sciences ☐ Behavioural & social sciences ☐ Ecological, evolutionary & environmental sciences

For a reference copy of the document with all sections, see nature.com/documents/nr-reporting-summary-flat.pdf

Life sciences study design

All studies must disclose on these points even when the disclosure is negative.

Sample size	No sample-size calculations were performed. Sample size was determined to be adequate based on the magnitude and consistency of measurable differences between groups.
Data exclusions	After the quality control, individual cells with high number of reads mapping, that is dead or damaged cells, were excluded from the analyses, according to the count of feature genes, the percentage of mitochondrial transcripts, and the percentage of red blood cells.
Replication	Replicate experiments were successful.
Randomization	Randomization was not relevant to this study because it was not required in the study.
Blinding	Blinding was not relevant to this study because no bias could be made by the subject or the tester in the analyses performed.

Reporting for specific materials, systems and methods

We require information from authors about some types of materials, experimental systems and methods used in many studies. Here, indicate whether each material, system or method listed is relevant to your study. If you are not sure if a list item applies to your research, read the appropriate section before selecting a response.

Materials & experimental systems

n/a	Involved in the study
☐	☒ Antibodies
☒	☐ Eukaryotic cell lines
☒	☐ Palaeontology and archaeology
☒	☐ Animals and other organisms
☐	☒ Clinical data
☒	☐ Dual use research of concern

Methods

n/a	Involved in the study
☒	☐ ChIP-seq
☐	☒ Flow cytometry
☒	☐ MRI-based neuroimaging

Antibodies

Antibodies used: All antibodies used in this study are detailed in "Supplementary Table S1".

Validation: All antibodies are commercially available and were commercially validated.

Clinical data

Policy information about clinical studies
All manuscripts should comply with the ICMJE guidelines for publication of clinical research and a completed CONSORT checklist must be included with all submissions.

Clinical trial registration: chictr.org.cn (ChiCTR2100051405)

Study protocol: Study protocol can be found in "Supplemantary File. S3".

Data collection: Setting: patients with vitiligo treated by autologous cultured epithelium grafting in the dermatology department of Huashan Hospital affiliated to Fudan University were required to fill in the questionnaire item by item. The basic data and personal history of the patient are required to be complete, preoperative treatment methods, curative effects, postoperative adverse reactions and complications.
Collection time: autologous cultured epithelium grafting was completed from November 1, 2015 to June 30, 2019, and the last follow-up time was December 31, 2022.

Outcomes: At the end of 6 months after the operation, the patients were evaluated the repigmentation rates according to the follow-up photos of the patients, and the repigmentation rates was independently evaluated by two dermotologists. Finally, the total effective rate was counted. The evaluation criteria were based on the clinical classification and curative effect criteria of vitiligo of pigmentation group of Dermatology and venereal diseases Professional Committee of Chinese society of integrated traditional and Western Medicine (revised in 2003):
Exellent improvement is all leukoplakia subsided and returned to normal skin color; Good improvement is that the leukoplakia partially subsides or shrinks, and the area of restoring normal skin color accounts for ≥ 50% of the lesion area; Fair improvement is that the leukoplakia partially subsides or shrinks; Poor improvement is that leukoplakia has no pigment regeneration or range expansion.

Flow Cytometry

Plots

Confirm that:

☒ The axis labels state the marker and fluorochrome used (e.g. CD4-FITC).

☒ The axis scales are clearly visible. Include numbers along axes only for bottom left plot of group (a 'group' is an analysis of identical markers).

☒ All plots are contour plots with outliers or pseudocolor plots.

☒ A numerical value for number of cells or percentage (with statistics) is provided.

Methodology

Sample preparation: Cell suspensions were prepared by digesting epidermal sheets with dispase II (STEMCELL) to achieve concentrations exceeding 1 × 10^6/ml.

Instrument: BD LSRFortessa was used to analyze samples.

Software: BD software and FlowJo version 10.4.0. were used to analyzed the data of flow cytometry.

Cell population abundance	Over 10000 cells were analyzed for fluorescent intensity in the defined gate.
Gating strategy	A gate is drawn around the cells. Single cells are determined with the area and the height of the side scatter (SSC). The analysis was carried out in this gate.

☒ Tick this box to confirm that a figure exemplifying the gating strategy is provided in the Supplementary Information.
